# Synthesis of CuO nanoparticles stabilized with gelatin for potential use in food packaging applications

**DOI:** 10.1038/s41598-022-16878-w

**Published:** 2022-07-27

**Authors:** A. A. Gvozdenko, S. A. Siddiqui, A. V. Blinov, A. B. Golik, A. A. Nagdalian, D. G. Maglakelidze, E. N. Statsenko, M. A. Pirogov, A. A. Blinova, M. N. Sizonenko, A. N. Simonov, R. B. Zhukov, R. O. Kolesnikov, S. A. Ibrahim

**Affiliations:** 1 North-Caucasus Federal University, Pushkina str. 1, 355017 Stavropol, Russia; 2 Campus Straubing for Biotechnology and Sustainability, Technical University of Munich, Essigberg 3, 94315 Straubing, Germany; 3 German Institute of Food Technologies (DIL e.V.), Prof.-von-Klitzing-Straße 7, 49610, D Quakenbrück, Germany; 4 Saint Petersburg State Agrarian University, Peterburgskoe Highway 2, 196601 Pushkin, Saint Petersburg, Russia; 5 Stavropol State Agrarian University, Zootekhnichesky Avenue 12, 355017 Stavropol, Russia; 6 Don State Agrarian University, Krivoshlykova Street 24, 346493 Persianovsky, Russia; 7 North Carolina Agricultural and Technical State University, E. Market Street, 1601,, 24711 Greensboro, NC USA

**Keywords:** Nanobiotechnology, Nanoscale materials, Other nanotechnology, Chemical engineering

## Abstract

In the present study, a method for the synthesis of gelatin-stabilized copper oxide nanoparticles was developed. Synthesis was carried out by direct chemical precipitation. Copper sulfate, chloride, and acetate were used as precursors for the copper oxide synthesis. Gelatin was used as a stabilizer. It was found that the formation of monophase copper oxide II only occurred when copper acetate was used as a precursor. Our results showed that particles of the smallest diameter are formed in an aqueous medium (18 ± 6 nm), and those of th largest diameter—in an isobutanol medium (370 ± 131 nm). According to the photon correlation spectroscopy data, copper oxide nanoparticles synthesized in an aqueous medium were highly stable and had a monomodal size distribution with an average hydrodynamic radius of 61 nm. The study of the pH effect on the colloidal stability of copper oxide nanoparticles showed that the sample was stable in the pH range of 6.8 to 11.98. A possible mechanism for the pH influence on the stability of copper oxide nanoparticles is described. The effect of the ionic strength of the solution on the stability of the CuO nanoparticles sol was also studied, and the results showed that Ca^2+^ ions had the greatest effect on the sample stability. IR spectroscopy showed that the interaction of CuO nanoparticles with gelatin occurred through the hydroxyl group. It was found that CuO nanoparticles stabilized with gelatin have a fungicidal activity at concentration equivalent 2.5 · 10^−3^ mol/L and as a material for food nanopackaging can provide an increase in the shelf life of products on the example of strawberries and tomatoes. We investigated the possibility of using methylcellulose films modified with CuO nanoparticles for packaging and storage of hard cheese “Holland”. The distribution of CuO nanoparticles in the methylcellulose film was uniform. We found that methylcellulose films modified with CuO nanoparticles inhibited the growth and development of QMAFAM, coliforms, yeast and mold in experimental cheese sa mples. Our research has shown that during the cheese storage in thermostat at 35 ± 1 °C for 7 days, CuO nanoparticles migrated to the product from the film. Nevertheless, it is worth noting that the maximum change in the concentration of copper in the experimental samples was only 0.12 µg/mg, which is not a toxic concentration. In general, the small value of migration of CuO nanoparticles confirms the high stability of the developed preparation. Our results indicated that the CuO nanoparticles stabilized with gelatin have a high potential for use in food packaging – both as an independent nanofilm and as part of other packaging materials.

## Introduction

Copper (II) oxide (CuO) is known as a p-type semiconductor with a narrow bandgap that ranges from 1.9 to 2.1 eV ^1^. This material has prospective useful physical properties, such as high-temperature superconductivity, electron correlation effects, and spin dynamics^2,3^. CuO nanoparticles have found wide application in various branches of science and technology including electronics^4,5^, agriculture^6–8^, medicine^9,10^, solar energy^11–13^. CuO nanoparticles can be used to remove organic pollutants in wastewater. In particular, model experiments were carried out on the following organic dyes: methyl orange, methyl red, Congo red, methylene blue, Nile blue, Reactive Yellow 160, etc^14–17^. Other studies showed that the efficiency of catalytic materials based on CuO nanoparticles reached more than 90%^15,16^. The use of CuO nanoparticles for the production of gas sensors for CO, H_2_S detection was also reported^18–21^. In^18^, a gas sensor based on CuO nanoparticles obtained by the sol–gel method was presented. It was found that at a concentration of 0.1 ppm ethanol in air, the sensitivity of this sensor was 2.7 Rg/Ra, where “Rg”—is the sensor resistance in the target gas, and “Ra”—is the sensor resistance in dry air. Hou L. et al.^20^ developed a CO sensor based on CuO nanostructures, in which the sensitivity was 3.27 Rg/Ra. In addition, such as hemoglobin synthesis, iron oxidation, cellular respiration and amidation of antioxidant defense peptides^22,23^.However, the use of CuO nanoparticles in food formulations is still limited due to the enhanced toxicity^24–26^. CuO nanoparticles at concentration 1–50 µg/ml have cytotoxical effect on HepG2 cells^27^ and human TT1 cells^28^ in dose-dependent manner. To improve the possibility of their use in the food industry, researchers seek to find optimal approaches to the synthesis and stabilization of CuO nanoparticles in order to reduce toxicity, but at the same time to keep or even improve their useful properties.

Nanoparticles are often used in the food industry to create antibacterial films^29,30^. Today, research is underway to develop antimicrobial packaging materials obtained using various nanoparticles, including CuO^31–33^. Nanopackaging can be applied to a food product by wrapping, dipping, brushing, or spraying to provide a selective barrier against the movement of gases, moisture, and dissolved materials as well as protection against mechanical damage^34,35^. The main developments are aimed at obtaining nanoparticles with subsequent processing of the surface of ready-made packaging materials. However, works considering the modification of polymer coatings by CuO nanoparticles due to immobilization are extremely rare^36^. When nanoparticles are introduced directly into the structure of polymer materials, “closure” occurs—inhibition of the active component in the volume, as a result of which the material loses antimicrobial properties^37,38^. According to many researchers, the activity of nanoparticles depends on the shape and their dispersion^39,40^. At the same time, most of the works are devoted to studies of their bactericidal properties and, to a lesser extent, fungicidal properties are considered. The obtained results of the fungicidal activity of nanoparticles in many studies more often show the absence of a reliable pattern, but Nazarzade and Ghorbani (2019) in their work established the fungicidal activity of CuO nanoparticles^41^. An important aspect in the design of food packages with nanocompositions is the stabilization of nanoparticles. The stability of nanoparticles in the polymer composition of packaging materials is a condition for bactericidal activity and migration of nanoparticles into the product^42,43^ and depends on the method of synthesis. With high stability, migration of CuO nanoparticles into the product will be excluded, which will guarantee the absence of toxicity of the packaging material.

There are a number of methods for preparing CuO nanoparticles: the sol–gel method^44–46^, sonochemical method^47,48^, hydrothermal method^49,50^, reverse micelle method^51^, and the exploding wire method^42,52,53^. In^43^, an electrochemical method for obtaining CuO nanostructures of various shapes and sizes is presented. Sodium nitrate solution was used as an electrolyte, and Cu—as an anode. Synthesis was carried out in an undivided cell in a constant current mode at room temperature. It was found that with a decrease in the current density, monodisperse, homogeneous CuO nanorods or nanoparticles could be obtained, and with an increase—nanocrystals of irregular shape were formed. There are also known works on the preparation of CuO nanoparticles by “green chemistry” methods^54–56^. Apriandanu, D. O. B. and Yulizar, Y. the extract of Tinospora crispa leaves was used to obtain CuO nanoparticles^55^. According to the transmission electron microscopy data, it was found that the obtained nanoparticles had a diameter of 10 to 40 nm and were characterized by a spherical shape.

It is important to note that for use in real systems, nanoparticles must be stabilized in order to prevent aggregation, coagulation, and sedimentation, which lead to particle coarsening and loss of physicochemical properties associated with the nanoscale state^57–60^. For example, Cai Z. et al.^61^, the effect of stabilized and unstabilized Fe nanoparticles on the reductive degradation of nitrobenzene was compared. Carboxymethyl cellulose was used as a stabilizer. It was shown that stabilized Fe nanoparticles decomposed nitrobenzene 3.7 times faster than the unstabilized analogue. The authors noted that this effect was associated with the particle size: stabilized Fe nanoparticles had a diameter of 17 nm, and unstabilized ones—more than 1000 nm. In an unstabilized sample, the particles were combined into clusters, which led to an increase in the particle size and, consequently, to a decrease in the specific surface area of the particles, which accounted for the lower reducibility.

Various surfactants such as the following can be used to stabilize CuO nanoparticles: sodium dodecyl sulfate, cetyltrimethylammonium bromide, alkylhydroxyethyldimethylammonium chloride^48,60,62^, polyvinylpyrrolidone (PVP)^63^, monomers acrylonitrile and methyl methacrylate^64^, polyethylene glycol (PEG)^65,66^, etc. However, there is no mention in the literature about the use of gelatin to stabilize copper oxide nanoparticles. Gelatin is a food ingredient that is a mixture of linear polypeptides with different molecular weights. There are up to 18 amino acids in gelatin, including glutamic and aspartic acids, glycine, proline, hydroxyproline, alanine and arginine. Gelatin contains both negatively charged carboxyl and hydroxyl groups as well as positively charged amino groups. The stabilization of CuO nanoparticles can occur by means of the aforementioned groups^67^. The aim of this work was thus to develop a method for synthesizing CuO nanoparticles stabilized with gelatin, to study their colloidal stability in various dispersion media and to explore the possibility of their use in food packaging applications.

## Materials and methods

### Materials

Copper (II) acetate (GR for analysis, “Mikhailovsky Plant of Chemical Reagents”, Barnaul, Russia), copper (II) sulfate (GR for analysis, LLC “Khimsnab-2000”, Rostov-on-Don, Russia), copper (II) chloride (CP, LLC Formula, St. Petersburg, Russia), gelatin (Grade P-140, LLC TD-holding, Krasnodar, Russia), sodium hydroxide (GR for analysis, LLC Povolzhye, Dzerzhinsk, Russia), ethyl alcohol (CP, Merck, Germany), propyl alcohol (CP, Merck, Germany), butyl alcohol (CP, Merck, Germany), isobutyl alcohol (CP, Merck, Germany), isopropyl alcohol (CP, Acros Organics, Belgium), phosphoric acid (CP, AO LenReaktiv, Russia), acetic acid (CP, AO LenReaktiv, Russia), boric acid (CP, AO LenReaktiv, Russia), sodium chloride (CP, AO LenReaktiv, Russia), calcium chloride (GR for analysis, AO LenReaktiv, Russia), sodium sulfate (GR for analysis, ORT Khimreaktivy, Russia), phenolphthalein (CP, AO LenReaktiv, Russia), trilon B (CP, AO LenReaktiv, Russia), sodium diethyldithiocarbamate (CP, Chemical Line, Rusia), chloroform (CP, AO LenReaktiv, Russia).

### Synthesis of CuO nanoparticles

CuO nanoparticles stabilized with gelatin were obtained by direct chemical precipitation. Copper (II) acetate, copper (II) sulfate, and copper (II) chloride were used as precursors of CuO nanoparticles. Gelatin acted as a stabilizer, sodium hydroxide as a precipitant. Distilled water, ethyl alcohol, propyl alcohol, butyl alcohol, isobutyl alcohol, and isopropyl alcohol were used as the reaction media.

CuO nanoparticles stabilized with gelatin were obtained by the following procedure: 2 g of copper precursor (copper II acetate, copper II sulfate, copper II chloride) and 2 g of gelatin were dissolved in 90 mL of the reaction medium (distilled water, propanol, isopropanol, butanol, or isobutanol). The resulting solution was heated to t = 90 °C with constant stirring, and further, 5 mL of a 10 M NaOH solution was added. The sample was mixed for 5 min, cooled to room temperature, stirred at room temperature for 25 min. Then we centrifuged the samples for 5 min at 5000 rpm, decanted the supernatant, and added bidistilled water to the resulting sediment. The procedure was repeated 3 times. As a result, a sol of copper oxide nanoparticles was formed.

### Characterization of synthesized CuO nanoparticles

Micrographs of CuO nanoparticles’ samples and data on the elemental composition were obtained using a scanning electron microscope MIRA3-LMH with a system for determining the elemental composition AZtecEnergy Standard/X-max 20 (standard), Tescan. The samples were dried for the study. Sample preparation was carried out as follows: a double-sided conductive carbon tape was glued to a standard instrument table (12 mm). Then *CuO* powder was applied onto the conductive carbon tape. Then, a carbon coating with a thickness of about 10 nm was deposited.

The parameters of the measurement were as follows:

• Voltage 10 kV.

• Work Distance 4.9 mm.

• In-Beam SE detector.

The phase composition of the CuO samples was investigated by X-ray diffraction analysis on an Empyrean diffractometer (PANalytical, Almeo). The following measuring parameters were used:

• Copper cathode (wavelength 1.54 Å).

• Measurement range 10–90 2θ°.

• Sampling frequency: 0.01 2θ°.

To study functional groups in the obtained samples, IR spectroscopy was used. IR spectra were recorded on an FSM-1201 IR spectrometer with Fourier transform. The measurement range was 400—4400 cm^-1^.

The determination of the average hydrodynamic radius of the particles was carried out by the dynamic light scattering (DLS) method on a Photocor-Complex instrument (Antek-97, Russia). Processing of the results was carried out using the DynaLS software.

The size of copper oxide nanoparticles was determined by electroacoustic spectroscopy on a DT-1202 setting (Dispersion Technology Inc.).

The molecular simulation was carried out in the IQmol molecular editor, the quantum-chemical calculations of the models were carried out using the QChem software with the following parameters: Calculation—Energy, method—M06, Basis—6-31G*, Convergence—4, Force field – Chemical^68^. To simplify calculations, free bonds were hydrogenated.

To determine the effect of the pH on the stability of CuO nanoparticles stabilized with gelatin, a series of buffer solutions with different pH values was prepared (from the pH of 1.81 to 11.98)^69^. For this, a solution of a mixture of phosphoric, acetic, and boric acids with a concentration of each acid of 0.04 M was prepared. Then, the required volume of 0.2 M NaOH solution was added to 100 mL of the acid mixture according to Table 1.

mL of a CuO nanoparticles sol was added to 63 mL of a buffer solution. The resulting solutions were kept for 30 min before measurement.

Three series of solutions were prepared to study the effect of the ionic strength of a solution on the stability of CuO nanoparticles^70^: sodium chloride (NaCl), calcium chloride (CaCl_2_), sodium sulfate (Na_2_SO_4_) solutions. The concentrations of the solutions were 0.1 M, 0.25 M, 0.5 M, 0.75 M, 1 M, and 1.5 M. For the estimation of stability 1 mL of the CuO nanoparticles sol was added to 9 mL of solution. The resulting samples were kept for 30 min before measurement.

The pH was measured using an Expert 001 pH meter-ionomer (Econix-Expert LLC, Russian Federation) using a combined silver-chloride electrode (EVL-1M3.1).

To study the fungicidal activity of CuO nanoparticles stabilized with gelatin, we used a disk diffusion method (Imani and Safaei 2019; Saedi, Shokri, and Rhim, 2020; Siddiqui et al., 2019). For this, we prepared solutions of CuO nanoparticles stabilized with gelatin with the next concentrations: 2.5 · 10^−3^ mol/L, 2.5 · 10^−4^ mol/L, 2.5 · 10^−5^ mol/L, 2.5 · 10^−6^ mol/L, 2.5 · 10^−7^ mol/L and 2.5 · 10^−8^ mol/L. Fungicidal activity was studied in relation to mold cultures of *Geotrichum candidum*, *Penicillium digitatum*, *Mucor racemosus*. The spore suspension of fungi was seeded on the surface of the nutrient agar medium.

**Table 1:** Buffer solutions.

pH	V(*NaOH*), mL	pH	V(*NaOH*), mL	pH	V(*NaOH*), mL
4.10	25.0	7.00	52.5	9.91	77.5
5.02	35.0	7.96	60.0	11.20	85.0
6.09	67.5	8.95	67.5	11.98	100.0

### Investigation of the effect of nanopackaging by CuO nanoparticles stabilized with gelatin on the shelf life of strawberries and tomatoes

Samples of strawberries and tomatoes were purchased from “Soil Respiration” LLC (Stavropol, Russia). The collection, storage and transportation of strawberry and tomato samples were carried out in accordance with the following Russian regulatory legal acts: GOST 34,298–2017 “Fresh tomatoes. Specifications” and GOST 33,953–2016 “Fresh strawberries. Specifications “.

To assess the potential of using CuO nanoparticles stabilized with gelatin as a food nanopackaging material, we conducted an experiment with tomatoes and strawberries.

Strawberries and tomatoes were treated according to the following procedure:

(1) preparation of 8 wt. % aqueous gelatin solution;

(2) stirring for 10 min;

(3) adding 2 wt. % glycerin;

(4) stirring for 10 min;

(5) addition of sol nanoparticles of copper oxide;

(6) stirring for 10 min;

(7) immersion of strawberries or tomatoes in the resulting solution with holding for 3 min.

The obtained strawberry and tomato samples were placed in a thermostat TC-1/80 (Smolensk SKTB SPU, Russia) at a temperature of 35 ± 1 °C for 7 days to carry out the test for the accelerated determination of shelf life^71^. The concentration of nanoparticles in the film was 2.5 · 10^−3^ mol/L.

### Preparation and study of methylcellulose films modified with CuO nanoparticles

**Figure 1:**
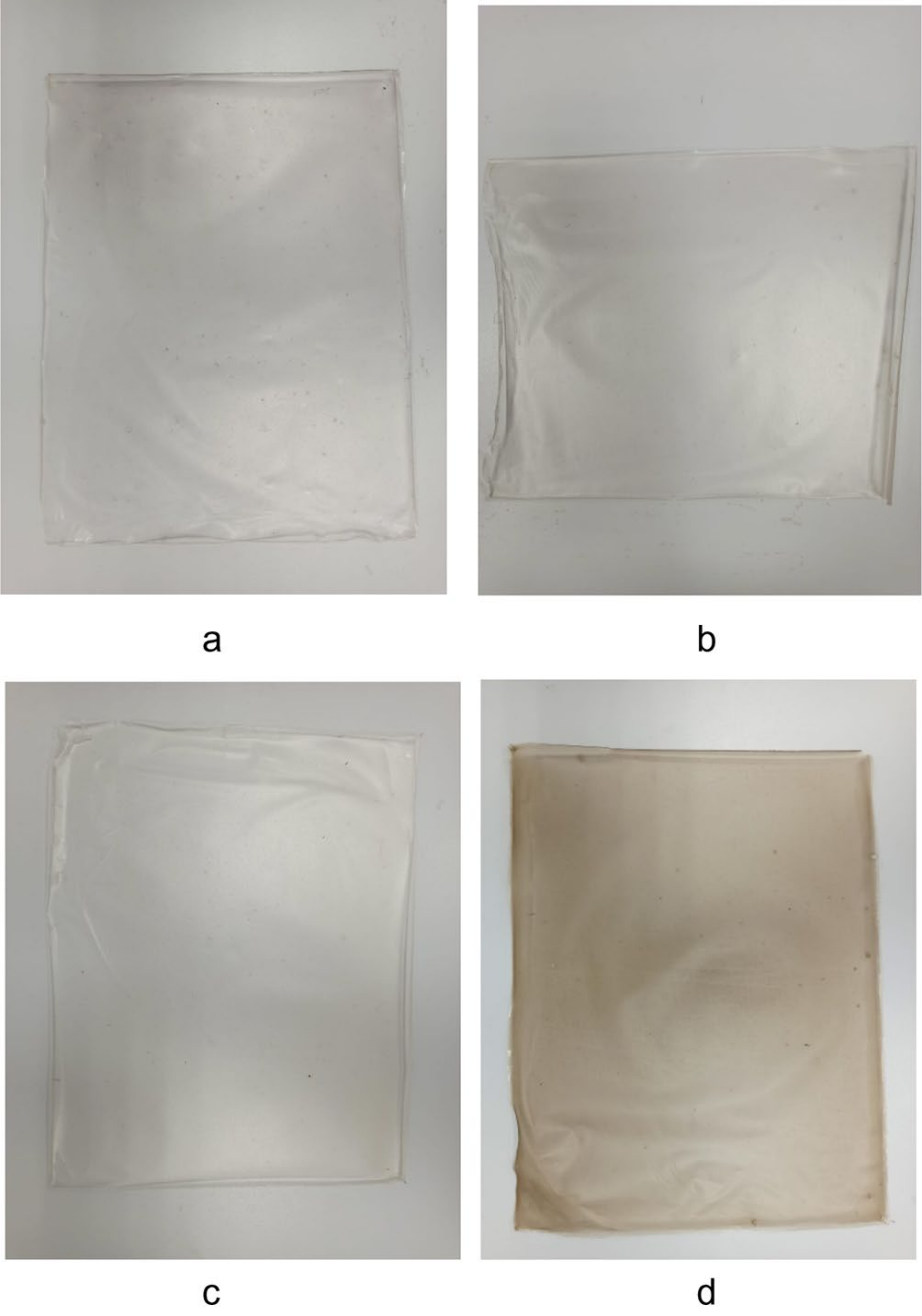
Photos of samples of methylcellulose films modified with CuO nanoparticles: a – control, b – 0.2%, c – 0.4%, d – 0.8%.

For preparation of packaging material modified with CuO nanoparticles we used a methylcellulose film, which is often used in cheese packaging in the Republics of the North Caucasus. The production of methylcellulose films was carried out on the basis of Nutrition Technologies LLC (Stavropol, Russia). We prepared conventional methylcellulose film as a control sample and methylcellulose films modified with 0.2%, 0.4% and 0.8% CuO nanoparticles as experimental samples. Photos of the obtained samples are shown in Fig. 1.

The obtained samples of methylcellulose films were studied by optical microscopy using an Axio Imager 2 (A2) research class microscope (Carl Zeiss Microscopy, Oberkochen, Germany) at various magnifications with image fixation using a specialized AxioCam MRc5 camera and Zen 2 Pro software (Carl Zeiss Microscopy, Oberkochen, Germany) ^72^.

Elemental composition of control and experimental methylcellulose films was studied using a scanning electron microscope MIRA3-LMH with a system for determining the elemental composition AZtecEnergy Standard/X-max 20 (standard), Tescan. The measurement was carried out with parameters described in "Characterization of synthesized CuO nanoparticles" Section.

### Investigation of the effect of methylcellulose films modified with CuO nanoparticles on quality and shelf life of cheese

For the experiment, we purchased at the MilkNet LLC store (Stavropol, Russia) a hard cheese “Holland” packed in polyethylene. The remaining shelf life at the time of purchase was 42 days. To study the initial parameters of the cheese on the day of the start of the experiment we made a slice weighing 10 ± 0.2 g. Slices weighing 10 ± 0.2 g were also prepared to form 4 experimental groups corresponding to the number of experimental methylcellulose films.

**Figure 2:**
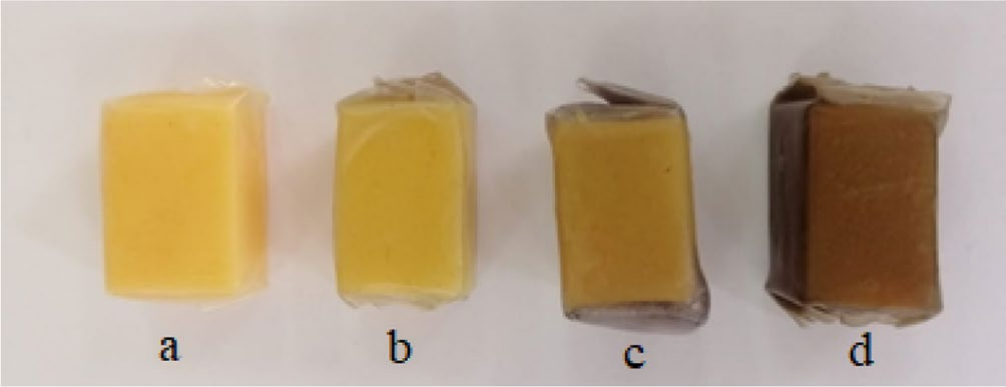
Experimental samples of hard cheese “Holland” packed in methylcellulose films modified with CuO nanoparticles: (**a**) control, (**b**) 0.2%, **c** 0.4%, (**d**) 0.8%.

Photos of the prepared samples of the four experimental groups are shown in Fig. 2.

To conduct an accelerated experiment, cheese samples were stored in thermostat TC-1/80 (Smolensk SKTB SPU, Russia) at a temperature of 35 ± 1 °C for 7 days.

The study of the titratable acidity of cheese samples was carried out by the indicator method according to Russian State Standard GOST 54,669–2011”Milk and milk processing products. Methods for determining titratable acidity”^73^. The method is based on the neutralization of free acids, acid salts and free acid groups contained in the product with a solution of sodium hydroxide in the presence of a phenolphthalein indicator.

The study of copper content in cheese samples packed in methylcellulose films modified with CuO nanoparticles was carried out by colorimetric method according to Russian State Standard GOST 26,931–86 “Raw materials and food products. Methods for determining copper”^74^. The cheese samples were incinerated and 5 ml of hydrochloric acid was added to the ash. 1 ml of copper solution was diluted with 100 ml of distilled water. 1 ml of the resulting solution was transferred to a separation funnel with 10 ml of citric acid and trilon B solution and 1 ml of phenolphthalein. Solution was neutralized with an aqueous solution of ammonia and brought to 100 ml of distilled water. Then 2 ml of sodium diethyldithiocarbamate solution and 15 ml of chloroform were added to the resulting mixture, shaken and left until the phases were separated. The substratum was poured into a measuring flask. 10 cm of solvent was added to the separation funnel, shaken, and after phase separation was poured into a measuring flask. The optical density of the obtained solutions was measured using an SF-56 optical spectrophotometer (OKB Spektr, Moscow, Russia).

For analysis of QMAFAM and coliforms during the storage of cheese we used 2 dilution: 1:100 and 1:1000 according to Russian State Standard GOST 32,901–2014 “Milk and milk products. Methods of microbiological analysis”^75^. From each dilution, 0.1 ml of suspension was sown into Petri dishes on a dense nutrient medium: ENDO and nutrient agar. The Inoculations were incubated in a thermostat for 24 h at 30 ± 1 °C. The number of grown coliforms was calculated and the results were expressed as log(CFU/g). QMAFAM was characterized by CFU/g. To determine the amount of yeast and mold during storage, after 1,3,5 and 7 days of storage in a thermostat, cheese samples were taken and placed in sterile bags with 90 ml of phosphate buffer (pH 7.2). Cheese samples were homogenized for 1 min in a homogenizer at 1000 rpm and a series of successive dilutions from 10^−1^ to 10^−9^ were prepared. To determine the amount of yeast and mold, 100 ml of the obtained solutions were carefully distributed on the surface of Saburo agar with glucose and chloramphenicol. Further, the samples were incubated at 24 °C for 5 days, the number of grown colonies of microorganisms was calculated and the results were expressed as log(CFU/g).

## Results and discussion

### Characterization of CuO nanoparticles

**Figure 3:**
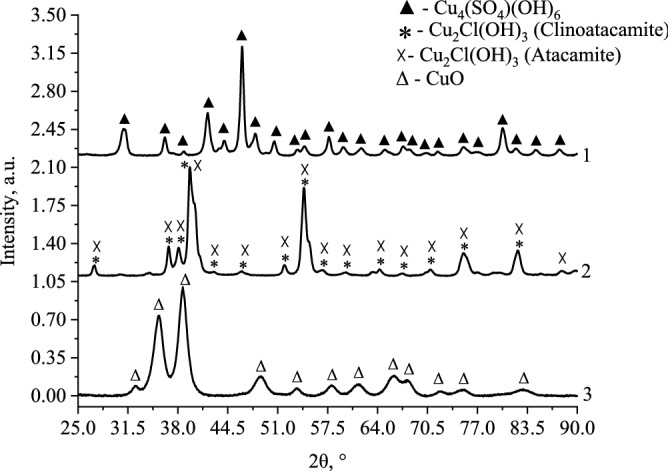
Diffraction patterns of samples, synthesized using copper (II) sulfate (1), copper (II) chloride (2), and copper (II) acetate (3).

At the first stage of research, CuO nanoparticles were obtained using various precursors, namely, copper (II) sulfate, copper (II) acetate, and copper (II) chloride. The synthesis was carried out in an aqueous medium. The obtained samples were investigated by powder diffractometry (XRD), the diffraction patterns are shown in Fig. 3.

Analysis of the diagrams in Fig. 3 showed that as a result of synthesis, various compounds are formed: when copper (II) acetate was used as a precursor, monophase CuO was formed (monoclinic crystal lattice, space group—C2/c)^76,77^. When copper (II) chloride was used as a precursor, copper (II) hydroxide chloride (Cu_2_Cl(OH)_3_) of two different modifications—atacamite (orthorhombic crystal lattice, space group—Pnam) and clinoatakamite (monoclinic crystal lattice, space group—P21/n) was obtained^78^. The content of atacamite in the sample was 50%, and that of clinoatakamite was 50%. When copper (II) sulfate was used, monophase brochantite (Cu_4_(OH)_6_SO_4_) was obtained (monoclinic crystal lattice, space group—P21/a)^79^. Further studies were carried out with only copper (II) acetate because it allows obtaining monophase CuO.

**Figure 4:**
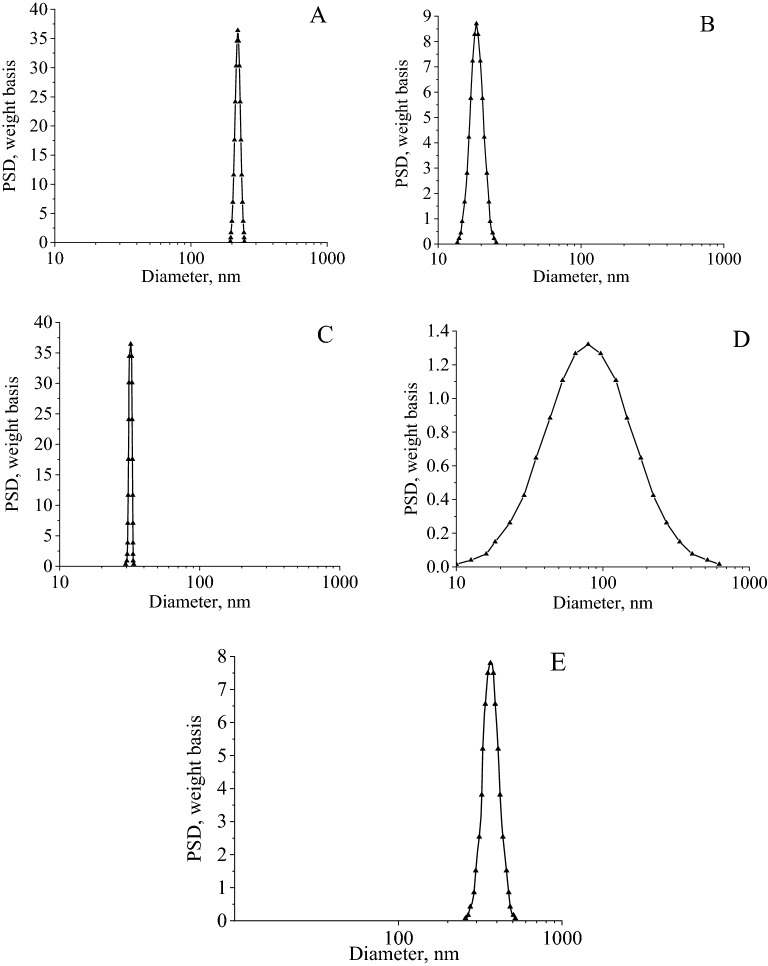
Size of CuO nanoparticles, synthesized in ethanol (**A**); in water (**B**); in isopropanol (**C**); in butanol (**D**); in isobutanol (**E**).

At the next stage of research, a series of CuO samples was synthesized in different media: distilled ethyl alcohol, water, isopropyl alcohol, butyl alcohol, isobutyl alcohol. The obtained samples were investigated by electroacoustic spectroscopy. In order to exclude the process of gelatin desolvation, synthesis in alcoholic media was carried out using water-alcohol solutions with alcohol content 85%.The data obtained are shown in Fig. 4.

The analysis of the obtained histograms showed that in all samples, the particles are characterized by a monomodal size distribution. It has been established that particles of the smallest diameter are obtained in an aqueous medium (18 ± 6 nm), and the largest diameter—in the isobutanol medium (370 ± 131 nm). CuO fractions with particles size of 211 ± 26 and 317 ± 21 nm were formed In ethanol and isopropanol respectively.

**Figure 5:**
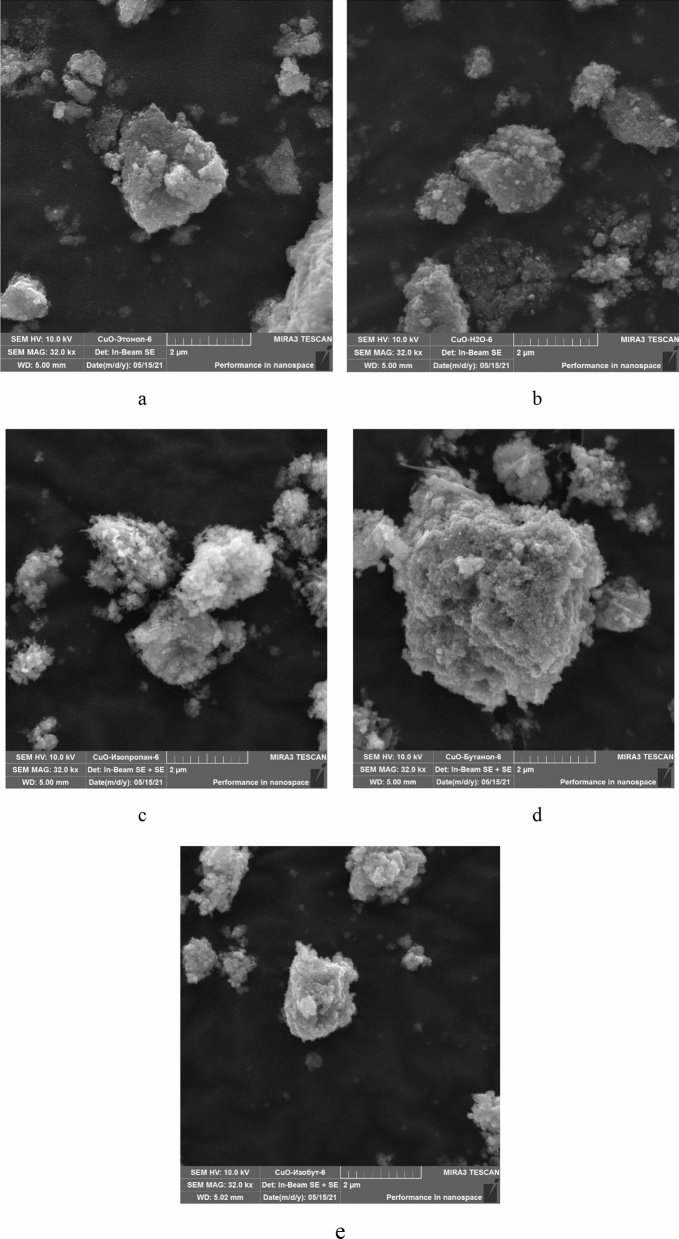
SEM image of the CuO sample obtained in: (**a**) – ethanol medium, (**b**) – aquatic medium; (**c**) – isopropanol medium; (**d**) – butanol medium; (**e**) – isobutanol medium.

At the next stage, CuO nanoparticles were studied using scanning electron microscopy. For research, the samples were dried. The data obtained are presented in Fig. 5.

SEM results demonstrated that all samples consist of aggregates of nanoparticles of 1.5 to 3 μm in diameter. It has been established that in the samples obtained in an aqueous medium, nanoparticles have a diameter of from 63 to 140 nm, in butanol—from 28 to 89 nm, in isobutanol—from 43 to 114 nm, in isopropanol—from 41 to 121 nm, In ethanol—from 35 to 142 nm.

### Quantum-chemical simulation of CuO nanoparticles’ stabilization with gelatin

At the next stage of research, the process of CuO nanoparticles' stabilization with gelatin was considered. Quantum-chemical simulation of gelatin parts containing 3 consecutively related amino acid molecules was carried out before interacting with the CuO molecule and after. Amino acids that have in their composition imino (NH) and hydroxyl (OH) groups, as well as carboxyl (COOH) and amino groups (NH_2_), which do not participate in the formation of peptide bonds, namely arginine (ARG), asparagine (ASN), aspartic acid (ASP), glutamine (GLN), glutamic acid (GLU), hydroxylysine (HYL), hydroxyproline (HYP), lysine (LYS), serine (SER) and threonine (THR) were considered.

As part of quantum-chemical calculations, the total energy of the molecular complex (E) was determined, as well as the energy of the highest occupied molecular orbital (HOMO) and the lower unoccupied molecular orbital (LUMO)^80^. The change in the total energy of the system (ΔE) was calculated using the Eq. 1:

1 $$\Delta E = E_{1} - E_{2} ,$$

where $E_{1}$– the energy of the model when interacting; $E_{2}$– the energy of the initial polymer.

Chemical hardness (η) was calculated using the Eq. 2^81,82^:

2 $$\eta = \frac{{E_{LUMO} - E_{HOMO} }}{2},$$

where $E_{LUMO}$– is the energy of the lower unoccupied molecular orbital (LUMO); $E_{HOMO}$ – is the energy of the highest occupied molecular orbital (HOMO).

The results of the quantum-chemical simulation are presented in Table 2 and Supplementary (Fig. S1–S68).

**Table 2:** Results of quantum-chemical simulation of the interaction of gelatin molecule sections with copper oxide.

	Amino acids sequence	*E*, kcal/mol	Δ*E*, kcal/mol	HOMO	LUMO	η
Arginine	Pro-Arg-Gly	− 1138.385	–	–	–	–
	Pro-Arg-Gly-CuO	− 2852.702	1714.317	− 0.078	− 0.013	0.033
	Ala-Arg-Gly	− 1061.086	–	–	–	–
	Ala-Arg-Gly-CuO	− 2701.317	1640.231	− 0.089	− 0.018	0.036
	Val-Arg-Gly	− 1139.655	–	–	–	–
	Val-Arg-Gly-CuO	− 2779.927	1640.272	− 0.110	− 0.013	0.049
Asparagine	Ala-Asn-Gly	− 947.184	–	–	–	–
	Ala-Asn-Gly-CuO	− 2661.418	1714.234	− 0.123	− 0.014	0.055
	Gly-Asn-Val	− 1025.736	–	–	–	–
	Gly-Asn-Val-CuO	− 2739.933	1714.197	− 0.098	− 0.002	0.048
	Leu-Asn-Gly	− 1065.044	–	–	–	–
	Leu-Asn-Gly-CuO	− 2779.271	1714.886	− 0.116	− 0.012	0.052
Aspartic acid	Leu-Asp-Gly	− 1084.902	–	–	–	–
	Leu-Asp-Gly-CuO	− 2798.600	1713.698	− 0.135	− 0.019	0.058
	Ala-Asp-Gly	− 967.050	–	–	–	–
	Ala-Asp-Gly-CuO	− 2680.763	1713.713	− 0.122	− 0.018	0.052
	Pro-Asp-Gly	− 1044.408	–	–	–	–
	Pro-Asp-Gly-CuO	− 2758.125	1713.717	− 0.117	− 0.016	0.055
Glutamine	Pro-Gln-Gly	− 1063.832	–	–	–	–
	Pro-Gln-Gly-CuO	− 2778.067	1714.235	− 0.125	− 0.013	0.056
	Phe-Gln-Gly	− 1217.350	–	–	–	–
	Phe-Gln-Gly-CuO	− 2931.562	1714.212	− 0.113	− 0.009	0.052
	Gly-Gln-Met	− 1463.173	–	–	–	–
	Gly-Gln-Met-CuO	− 3177.364	1714.191	− 0.100	− 0.007	0.047
	Val-Gln-Gly	− 1065.029	–	–	–	–
	Val-Gln-Gly-CuO	− 2779.238	1714.209	− 0.116	− 0.017	0.050
	Ala-Gln-Gly	− 986.477	–	–	–	–
	Ala-Gln-Gly-CuO	− 2700.684	1714.207	− 0.109	− 0.010	0.050
	Leu-Gln-Gly	− 1104.303	–	–	–	–
	Leu-Gln-Gly-CuO	− 2818.488	1714.185	− 0.104	− 0.013	0.046
Glutamic acid	Gly-Glu-Ala	− 1006.345	–	–	–	–
	Gly-Glu-Ala-CuO	− 2720.041	1713.696	− 0.125	− 0.013	0.056
	Val-Glu-Gly	− 1084.902	–	–	–	–
	Val-Glu-Gly-CuO	− 2798.599	1713.697	− 0.117	− 0.011	0.053
Hydroxylysine	Met-Hyl-Gly	− 1503.615	–	–	–	–
	Met-Hyl-Gly-CuO(1)	− 3217.343	1713.728	− 0.074	0.004	0.039
	Met-Hyl-Gly-CuO(2)	− 3217.825	1714.210	− 0.064	− 0.001	0.032
	Ile-Hyl-Gly	− 1144.744	–	–	–	–
	Ile-Hyl-Gly-CuO(1)	− 2858.472	1713.728	− 0.059	− 0.012	0.024
	Ile-Hyl-Gly-CuO(2)	− 2858.962	1714.218	− 0.067	− 0.017	0.025
Hydroxyproline	Leu-Hyp-Gly	− 1048.971	–	–	–	–
	Leu-Hyp-Gly-CuO	− 2762.664	1713.693	− 0.106	− 0.005	0.051
	Pro-Hyp-Gly	− 1008.465	–	–	–	–
	Pro-Hyp-Gly-CuO	− 2722.204	1713.739	− 0.090	0.002	0.046
	Ala-Hyp-Gly	− 931.117	–	–	–	–
	Ala-Hyp-Gly-CuO	− 2644.820	1713.703	− 0.105	− 0.002	0.052
	Phe-Hyp-Gly	− 1161.985	–	–	–	–
	Phe-Hyp-Gly-CuO	− 2875.719	1713.734	− 0.089	− 0.006	0.042
	Val-Hyp-Gly	− 1009.670	–	–	–	–
	Val-Hyp-Gly-CuO	− 2649.361	1639.691	− 0.111	− 0.004	0.054
	Met-Hyp-Gly	− 1407.814	–	–	–	–
	Met-Hyp-Gly-CuO	− 3047.776	1639.962	− 0.215	− 0.009	0.103
Lysine	Ala-Lys-Gly	− 951.718	–	–	–	–
	Ala-Lys-Gly-CuO	− 2665.932	1714.214	− 0.083	− 0.020	0.032
	Pro-Lys-Gly	− 1029.078	–	–	–	–
	Pro-Lys-Gly-CuO	− 2743.304	1714.226	− 0.073	− 0.020	0.027
Serine	Pro-Ser-Gly	− 931.110	–	–	–	–
	Pro-Ser-Gly-CuO	− 2644.841	1713.731	− 0.097	0.000	0.049
	Ala-Ser-Gly	− 853.768	–	–	–	–
	Ala-Ser-Gly-CuO	− 2567.480	1713.712	− 0.113	− 0.001	0.056
	Phe-Ser-Gly	− 1084.622	–	–	–	–
	Phe-Ser-Gly-CuO	− 2798.355	1713.733	− 0.099	− 0.006	0.047
Threonine	Pro-Thr-Gly	− 970.401	–	–	–	–
	Pro-Thr-Gly-CuO	− 2684.083	1713.682	− 0.081	− 0.012	0.035
	Leu-Thr-Gly	− 1010.874	–	–	–	–
	Leu-Thr-Gly-CuO	− 2724.601	1713.727	− 0.083	0.003	0.043
	Ala-Thr-Gly	− 890.050	–	–	–	–
	Ala-Thr-Gly-CuO	− 2605.748	1715.698	− 0.111	− 0.005	0.053

It was found that the energy of molecular systems “CuO-gelatin” is an order of magnitude lower than the energy of individual sections. This fact testifies to the energetically advantageous formation of the chemical bond between the gelatin sections and copper oxide^83^.

**Figure 6:**
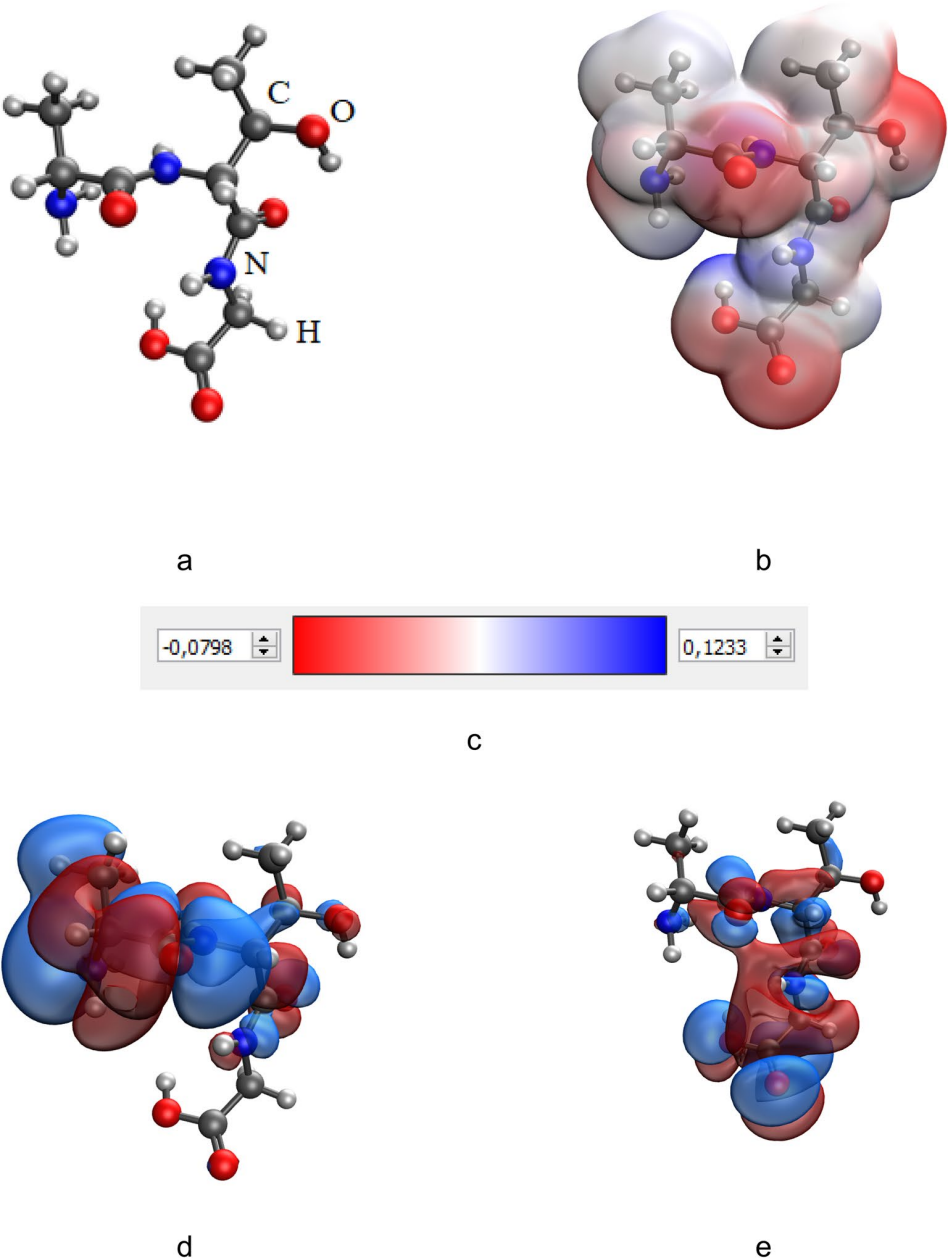
Results of the quantum-chemical simulation of the gelatin segment Ala-Thr-Gly: a model of the molecular complex (**a**), the distribution of electron density (**b**), electronic density distribution gradient (**c**), HOMO (**d**), LUMO (**e**).

**Figure 7:**
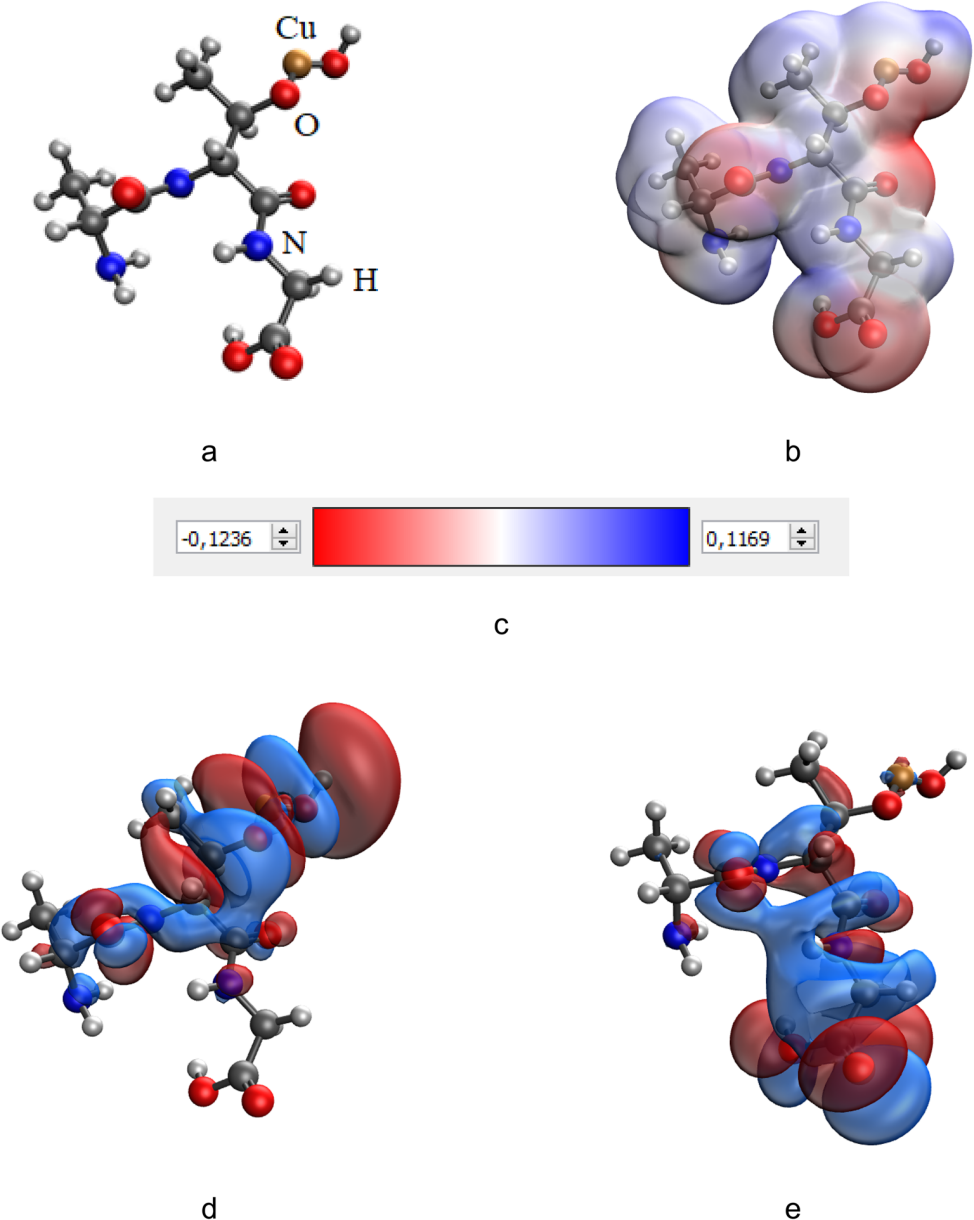
Results of the quantum-chemical simulation of the gelatin segment, bonded with CuO molecule (Ala-Thr-Gly-CuO): a model of the molecular complex (**a**), the distribution of electron density (**b**), electronic density distribution gradient (**c**), HOMO (**d**), LUMO (**e**).

It is important to note that the greatest change in the total energy of the system (ΔE) is observed in the case of the formation of the molecular system “Ala-Thr-Gly-CuO”, where the interaction occurs through the hydroxy group. Models of this system before and after interaction are presented in Figs. 6 and 7.

It has been established that chemical hardness in all considered systems is in the range of 0.024 to 0.103 eV. The minimum value of η observed at the Ile-Hyl-Gly-CuO molecular system, where the interaction occurs through the OH- group, the maximum η value—is at the Met-Hyp-Gly-CuO molecular system, where the interaction also occurs through the OH- group.

**Figure 8:**
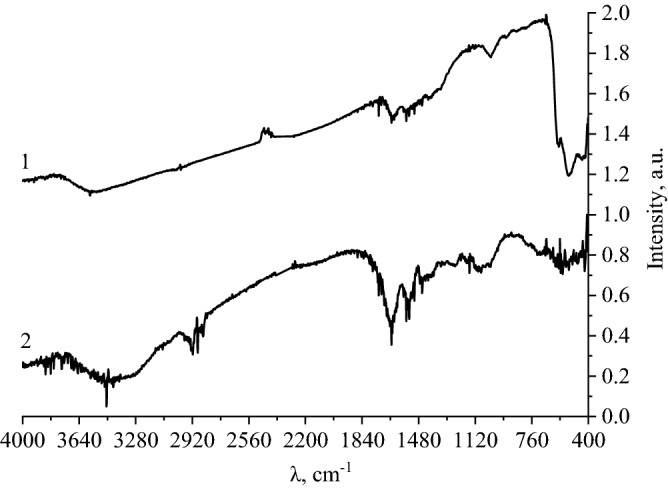
IR-spectra of CuO nanoparticles (1) and gelatin (2) samples.

To confirm the results of the simulation, an IR spectroscopy of CuO nanoparticles' samples was carried out. The obtained IR spectra of gelatin and CuO nanoparticles stabilized by gelatin are presented in Fig. 8.

Analysis of the gelatin and CuO IR spectra showed that in the range of 2800 cm^−1^—3600 cm^−1^ the presence of stretching vibration bands is observed: from 2851 to 2886 cm^−1^—CH_3_-, 2916 and 3424 cm^−1^—CH-, 3200—3500 cm^−1^—OH-, from 3464 to 3497 cm^−1^—NH- ^84^.

In the IR spectrum of gelatin in the range of 1000 to 1800 cm^−1^, bands characteristic of bending vibrations are observed: at 1022 and 1082 cm^−1^—symmetric vibrations of the OH– group, at 1157 cm^−1^—wagging vibrations of the -CH_2_ bond, at 1248 cm^−1^—vibrations of the C = O bond, the region from 1385 to 1471 cm^−1^ corresponds to symmetric vibrations of the -CH_3_ bond, the region from 1506 to 1576 cm^−1^ corresponds to the deformation vibrations of the ionized amino group NH_3_^+^, at 1653 cm^−1^—asymmetric vibrations of the carboxyl group COO-, the region from 1734 to 1773 cm^−1^ corresponds to the vibrations of the -CH_3_ bond. A peptide bond is also found, which is characterized by vibrations at 1630 and 1655 cm^−1^^85,86^.

In the same region from 1000 to 1800 cm^−1^ in the IR spectrum of CuO stabilized with gelatin, bands characteristic of bending vibrations are observed: at 1022 cm^−1^—symmetric vibrations of the OH– group, at 1153 cm^−1^—wagging vibrations of the -CH_2_ bond, the region from 1341 to 1458 cm^−1^ corresponds to symmetric vibrations of the -CH_3_ bond, the region from 1506 to 1559 cm^−1^ corresponds to the deformation vibrations of the ionized amino group NH_3_^+^, at 1653 cm^−1^—asymmetric vibrations of the carboxyl group COO-, at 1734 cm^−1^—deformation vibrations of the -CH_3_ bond. In the region from 400 to 850 cm^−1^, characteristic bands of –CH_2_ and –CH_3_ bonds vibrations are observed in the IR spectrum of gelatin^87^.

In the IR spectrum of CuO stabilized with gelatin, in the region from 400 cm^−1^ to 850 cm^−1^, there are bands characteristic of bending vibrations: the region from 673 to 850 cm^−1^ corresponds to bending vibrations of the -CH_3_ bond. High-intensity bands at 438, 527, and 588 cm^−1^ are due to the presence of Cu – O bonds, which are not observed in the spectrum of gelatin^88,89^.

As a result of the analysis of IR spectra, it was found that in the spectrum of CuO nanoparticles stabilized with gelatin, in the range of 3200 to 3500 cm^−1^, a significant decrease in the intensity of the bands characterizing the stretching vibrations of the hydroxy group is observed. Thus, it can be concluded that the interaction of CuO nanoparticles with gelatin occurs through the hydroxyl group.

### Colloidal stability of CuO nanoparticles

The next stage of research included the study of the stability of the obtained samples. It is important to note that as a result of the synthesis in a media of ethyl alcohol, isopropyl alcohol, butyl alcohol, and isobutyl alcohol, precipitates were formed, but in an aqueous medium—a sol. In this regard, a sample obtained in an aqueous medium was used to study the stability.

**Figure 9:**
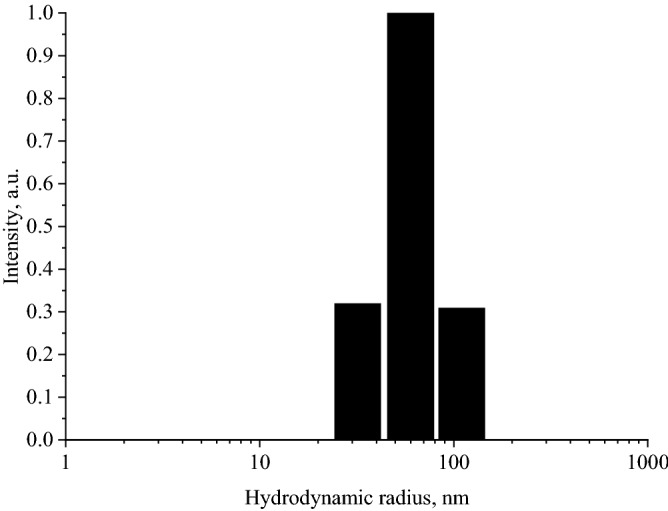
CuO nanoparticles' hydrodynamic radii distribution in the sol diluted 64 times with distilled water.

A study of the pH effect on the colloidal stability of CuO nanoparticles stabilized with gelatin was carried out. The samples were examined by photon correlation spectroscopy. Figure 9 shows a histogram of the distribution of hydrodynamic radii of CuO nanoparticles in the sol diluted 64 times with distilled water.

DLS study showed that the particles in the sample had a monomodal size distribution with an average hydrodynamic radius of 61 nm.

**Figure 10:**
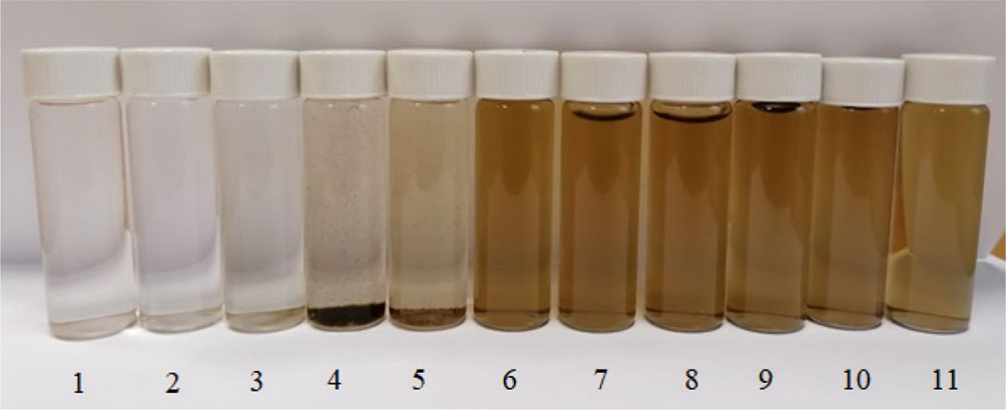
Photograph of a series of samples of CuO nanoparticles sol solutions: (1) at pH 1.81, (2) at pH 2.21, (3) at pH 3.29, (4) at pH 4.56, (5) at pH 5.72, (6) at pH 6.8, (7) at pH 7.96, (8) at pH 9.15, (9) at pH 10.38, (10) at pH 11.58, (11) at pH 11.98.

**Figure 11:**
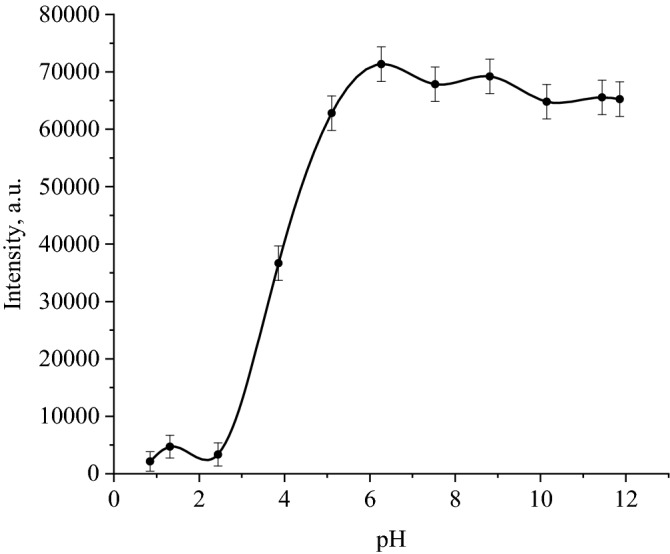
Dependence of the scattering intensity of the obtained CuO sols on the pH of the solution.

Figures 10 and 11 show photographs of the series of samples and the dependence of the scattering intensity of the solutions on the pH, respectively.

Data analysis (Figs. 10 and 11) showed that CuO nanoparticles dissolve in a strongly acidic medium (pH 1.81—3.29), as evidenced by the discoloration of the solution (Fig. 10) and low scattering intensity of the order of ≈ 3500 (Fig. 11 ). In samples with pH 4.56 and 5.72, particle coagulation was observed (Fig. 10). In the pH range of 6.8—11.98, there were no visible changes in the sample (Fig. 10) and a high scattering intensity was observed, which does not change significantly in this pH range and is comparable to the scattering intensity of an initial sample of a CuO nanoparticles sol diluted 64 times with distilled water (Fig. 11).

**Figure 12:**
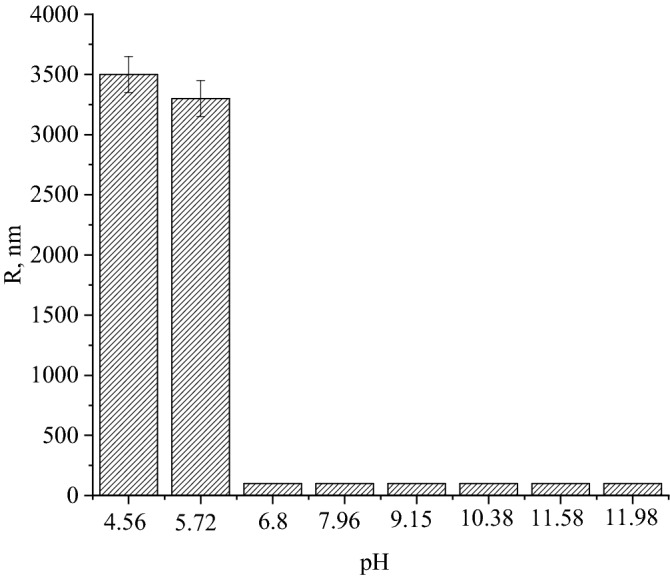
Dependence of the average hydrodynamic radius of particles (R) on the pH.

Figure 12 shows the dependence of the average hydrodynamic radius of particles (R) on the pH. For samples with a pH of 1.81 to 3.29, the average hydrodynamic radius was not determined.

In the samples with pH 4.56 and 5.72, the average hydrodynamic radius of the particles took on the values of 3578 and 3371 nm. Coagulation of particles in these samples was due to the attainment of the gelatin isoelectric point (pI = 4.7)^90^ and, accordingly, the loss of the surface electric charge. For samples with a pH of 6.8 to 11.98, the average hydrodynamic radius was 61 nm, which is comparable to the average hydrodynamic radius of particles in an initial sample of CuO nanoparticles diluted 64 times with distilled water. The data obtained indicate that the sol of CuO nanoparticles was stable in the pH range of 6.8 to 11.98.

**Figure 13:**
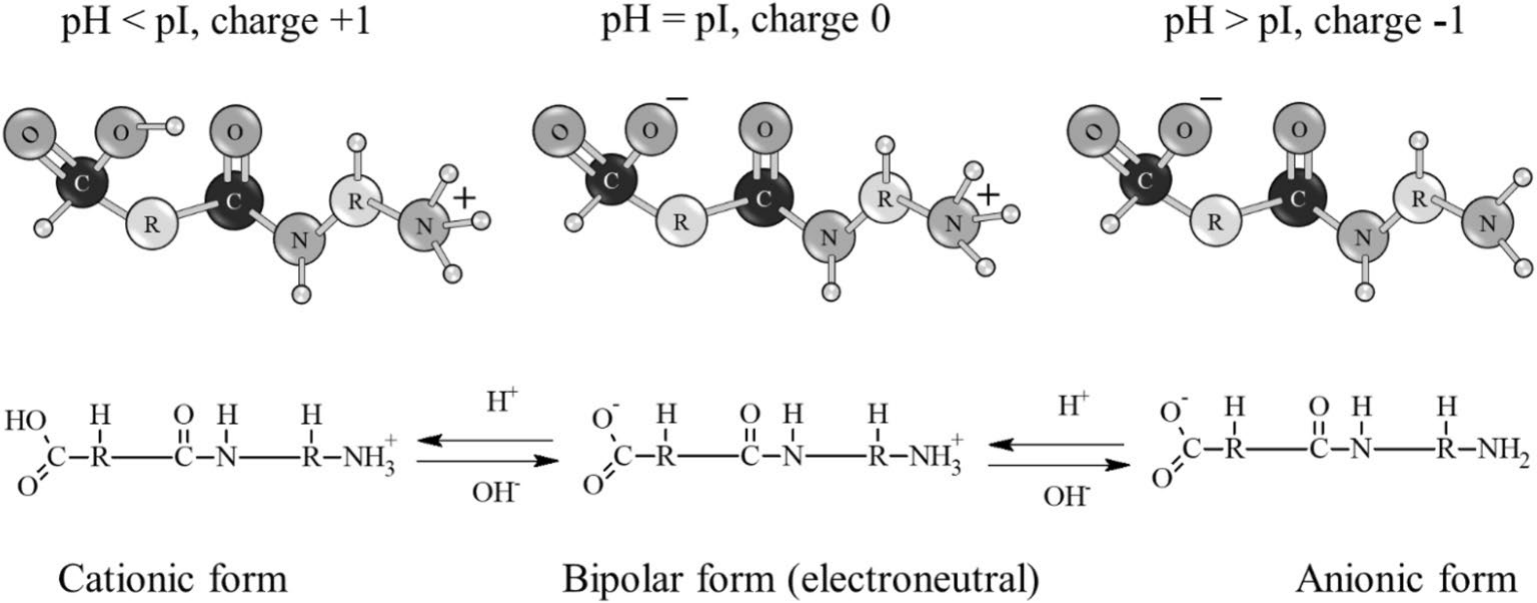
Scheme of protonation and deprotonation of a gelatin molecule.

The change in the size and stability of copper oxide nanoparticles at different pH values of the medium is due to the presence of free amino and carboxyl groups in the gelatin molecule, which allows it to exhibit amphiphilic properties. In an acidic environment, amino groups are activated, and in an alkaline environment, carboxyl groups are activated (Fig. 13).

When amino groups are protonated in an acidic medium, the gelatin molecule acquires a positive charge and imparts this charge to the micelle, but copper oxide is not stable in the acidic pH range, and it dissolves, thereby destroying the entire molecular structure of the copper-gelatin complex. With a decrease in the concentration of hydrogen ions (increase in pH), the rate of the protonation process slows down, and the equilibrium shifts in the opposite direction. As a result, the charge of the amino groups decreases and becomes equal to zero at the isoelectric point.

**Figure 14:**
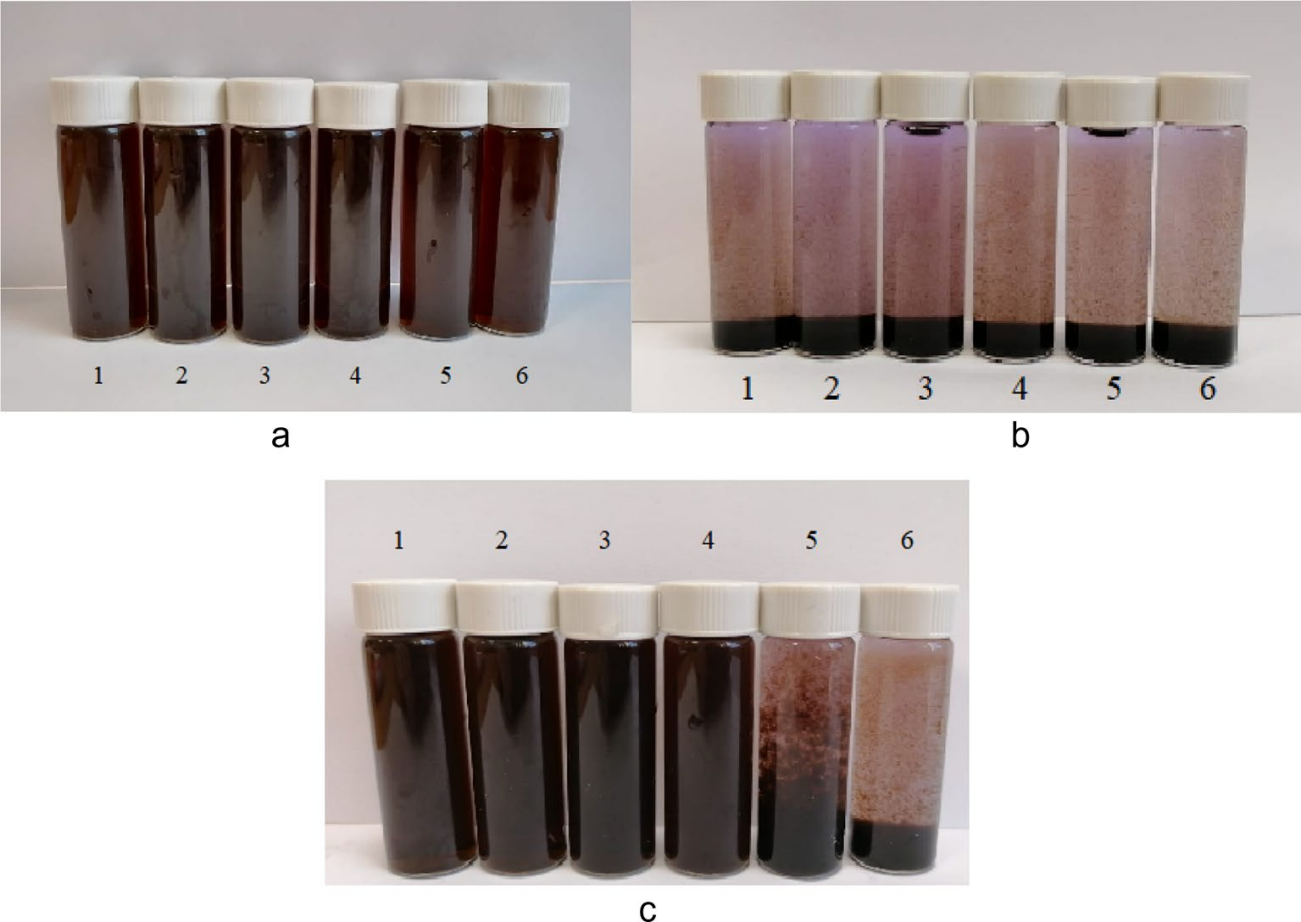
Photograph of CuO nanoparticles' solutions with various electrolytes: (**a)** – *NaCl*, (**b)** – *CaCl*_*2*_, **c** – *Na*_*2*_*SO*_*4*_ 1 – 0,1 M, 2 – 0,25 M, 3 – 0,5 M, 4 – 0,75 M, 5 – 1 M, 6 – 1,5 M.

**Figure 15:**
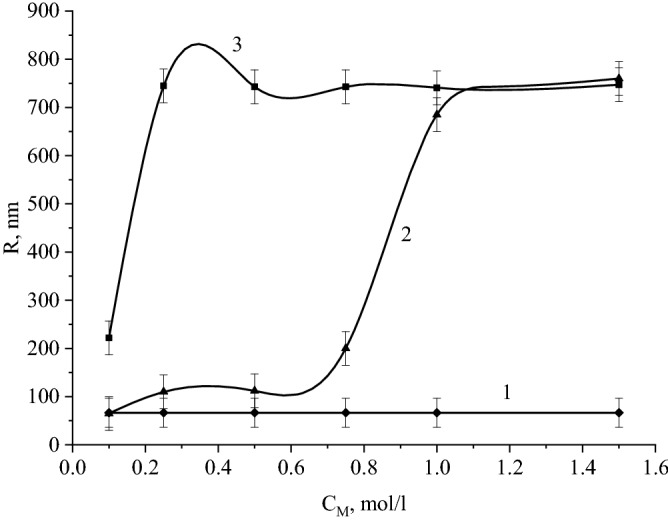
Dependence of the average hydrodynamic radius of copper oxide particles (R) on the concentration of electrolytes: 1 – *NaCl*, 2 – *Na*_*2*_*SO*_*4*_, 3 – *CaCl*_*2*_.

At the next stage, we studied the effect of the ionic strength of the solution on the stability of the CuO nanoparticles' sol. Figures 14 and 15 show photographs of the obtained samples and the dependence of the average hydrodynamic radius of particles (R) on the ionic strength of the solution, respectively.

In solutions with sodium chloride NaCl, no coagulation of CuO nanoparticles occurred, as evidenced by the absence of visible changes in the solution (Fig. 14a) and the absence of changes in the average hydrodynamic radius of the particles. When adding Na_2_SO_4_ with concentrations of 0.1 to 0.75 M in solutions, no changes occurred, in solutions with C_M_(Na_2_SO_4_) = 1 and 1.5 M, coagulation of CuO nanoparticles was observed (Fig. 14 c), and an increase in the average hydrodynamic radius of particles from 61 to 757 nm. By the addition of CaCl_2_, coagulation of particles was observed in all solutions (Fig. 14 b), the average hydrodynamic radius of the particles was ≈ 750 nm.

**Figure 16:**
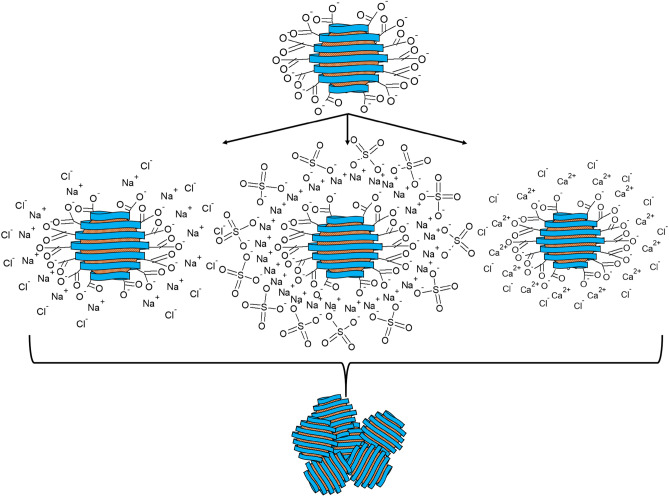
Scheme of CuO nanoparticles coagulation by various electrolytes.

It was found that the Ca^2+^ ions exert the greatest influence on the stability of the samples. The data obtained are in good agreement with the Schulze – Hardy rule^91^, as well as with the works of other authors^92^. A schematic representation of CuO nanoparticles coagulation with various electrolytes is shown in Fig. 16.

### Fungicidal activity of CuO nanoparticles stabilized with gelatin

**Figure 17:**
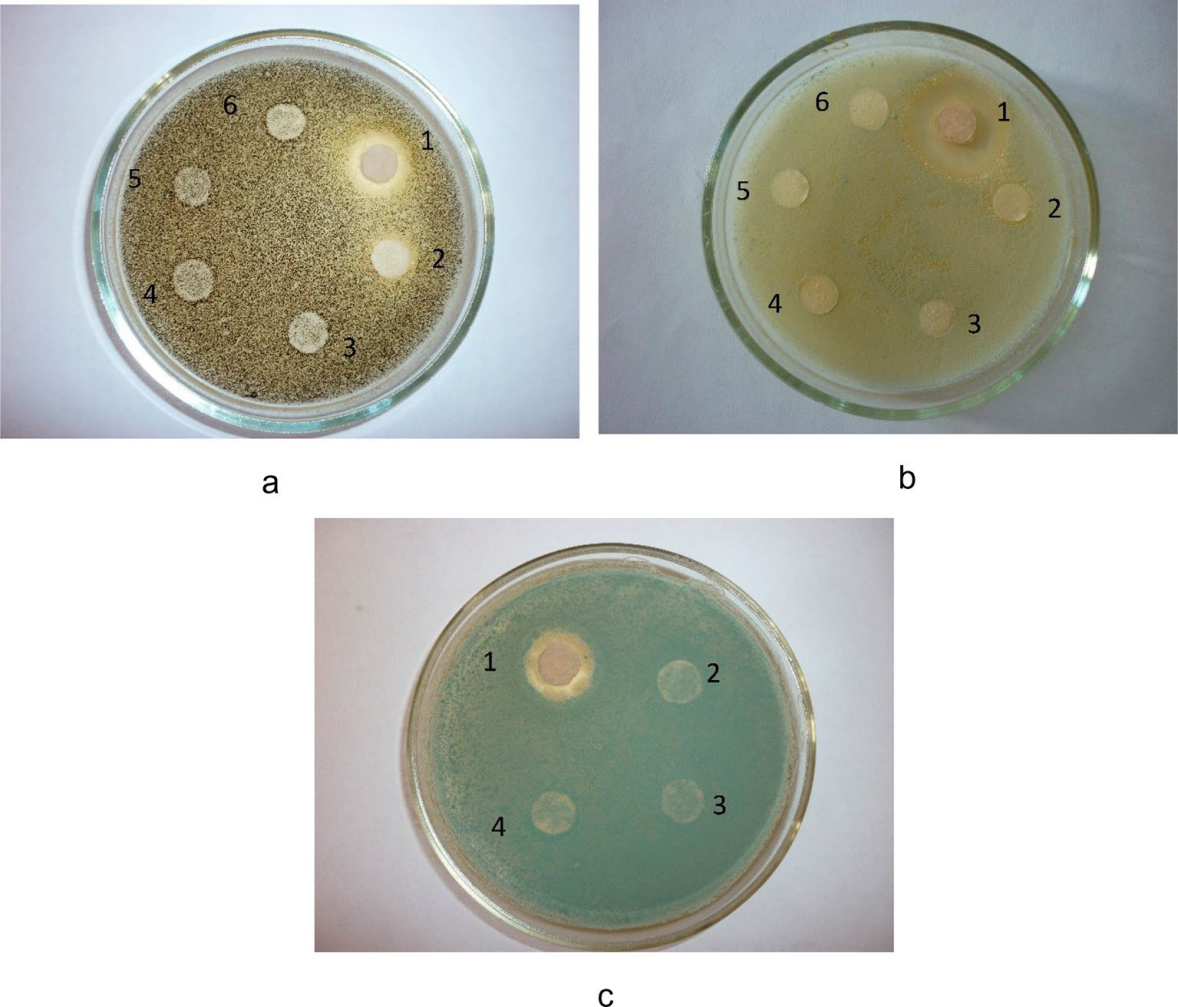
Effect of concentration of CuO nanoparticles stabilized with gelatin on fungicidal activity in relation to mold cultures: (**a)** – *Mucor racemosus*, (**b)** – *Geotrichum candidum*, (**c**) – *Penicillium digitatum*.

At the next stage we studied the fungicidal activity of the developed CuO nanoparticles stabilized with gelatin in relation to *Geotrichum candidum*, *Penicillium digitatum*, *Mucor racemosus* mold cultures. For this research we prepared solutions of CuO nanoparticles stabilized with gelatin with the next concentrations: 2.5 · 10^−3^ (№ 1), 2.5 · 10^−4^ (№ 2), 2.5 · 10^−5^ (№ 3), 2.5 · 10^−6^ (№ 4), 2.5 · 10^−7^ (№ 5), 2.5 · 10^−8^ mol/L (№ 6). The obtained results are showed in Fig. 17.

According to obtained results we found that the solution concentration of 2.5 · 10^−3^ mol/L (№ 1) has the greatest fungicidal activity. In this case, the suppression zone to *Mucor racemosus* is 5 mm, to *Geotrichum candidum* – 15 mm, and to *Penicillium digitatum* – 5 mm. At the solution concentration of 2.5 · 10^−4^ mol/L and lower (№ 2–6), we did not find fungicidal activity to studied mold cultures. Thus, the fungicidal activity of CuO nanoparticles stabilized with gelatin can be reflected at solution concentrations equivalent to 2.5 ·10^–3^ mol/L. Similar results of fungicidal activity of CuO nanoparticles were obtained recently by Consolo et al. (2020), Alagarasan et al. (2021) and Bramhanwade et al. (2015) ^93–95^.

### Effect of nanopackaging by CuO nanoparticles stabilized with gelatin on the shelf life of strawberries and tomatoes

**Figure 18:**
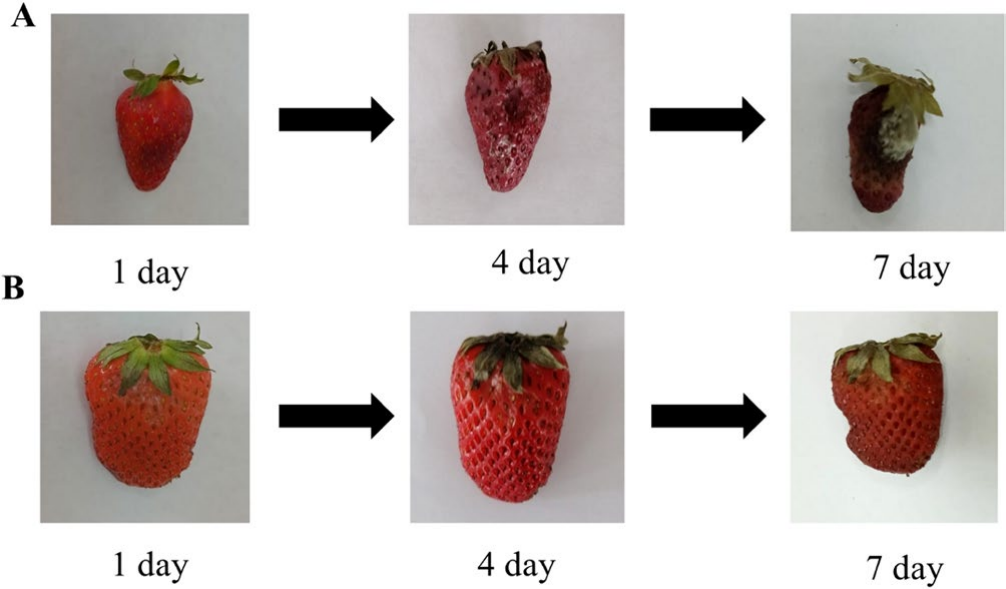
Photo of strawberry samples: (**A**) control sample without nanopackaging, (**B**) experimental sample with nanopackaging.

**Figure 19:**
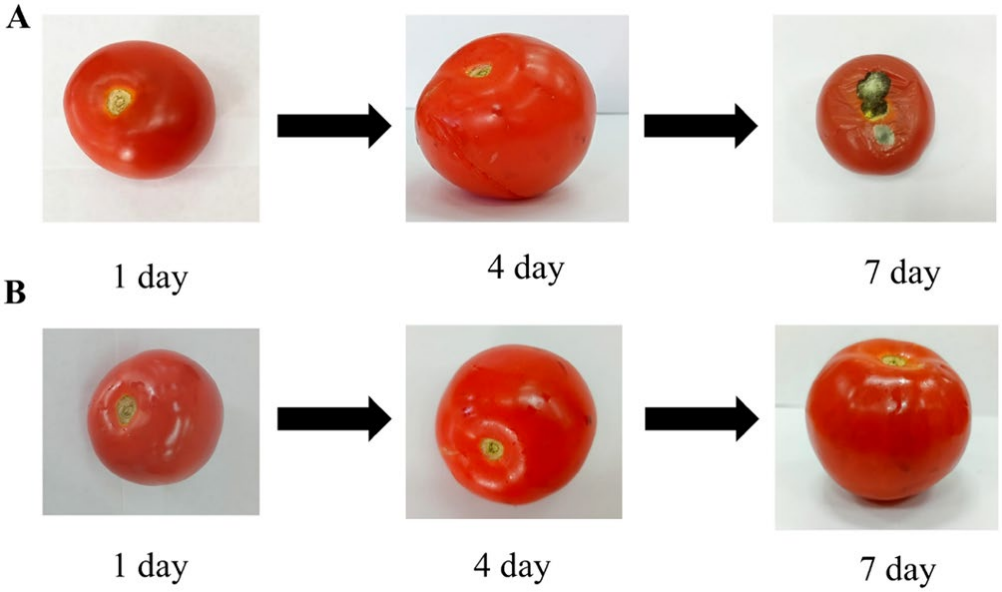
Photo of tomato samples: (**A**) control sample without nanopackaging, (**B**) experimental sample with nanopackaging.

Next, we investigated the effect of nanopackaging by CuO nanoparticles stabilized with gelatin on the shelf life of strawberries and tomatoes. As a result, photos of strawberry and tomato samples are shown in Figs. 18 and 19.

In control samples that were not treated with CuO nanoparticles, strawberry spoilage was observed on the 4th day of the experiment, and tomato spoilage on the 7th day. In the samples that were treated with nanoparticles, there was not spoilage of the product, which is associated with inhibition of the process of reproduction of bacteria and fungi that cause spoilage of strawberries and tomatoes. Our results correspond to the data of other researchers who studied the effect of nanopacking tomatoes and strawberries with Ag, TiO_2_, and Ti-doped CuO nanoparticles^71,96,97^.

Thus, we established that CuO nanoparticles stabilized with gelatin with a solution concentration equivalent to 2.5 ·10^−3^ mol/L can be used as a material for food nanopackaging, providing a bactericidal and fungicidal effect and increasing the shelf life of the product.

### Preparation and study of methylcellulose films modified with CuO nanoparticles

**Figure 20:**
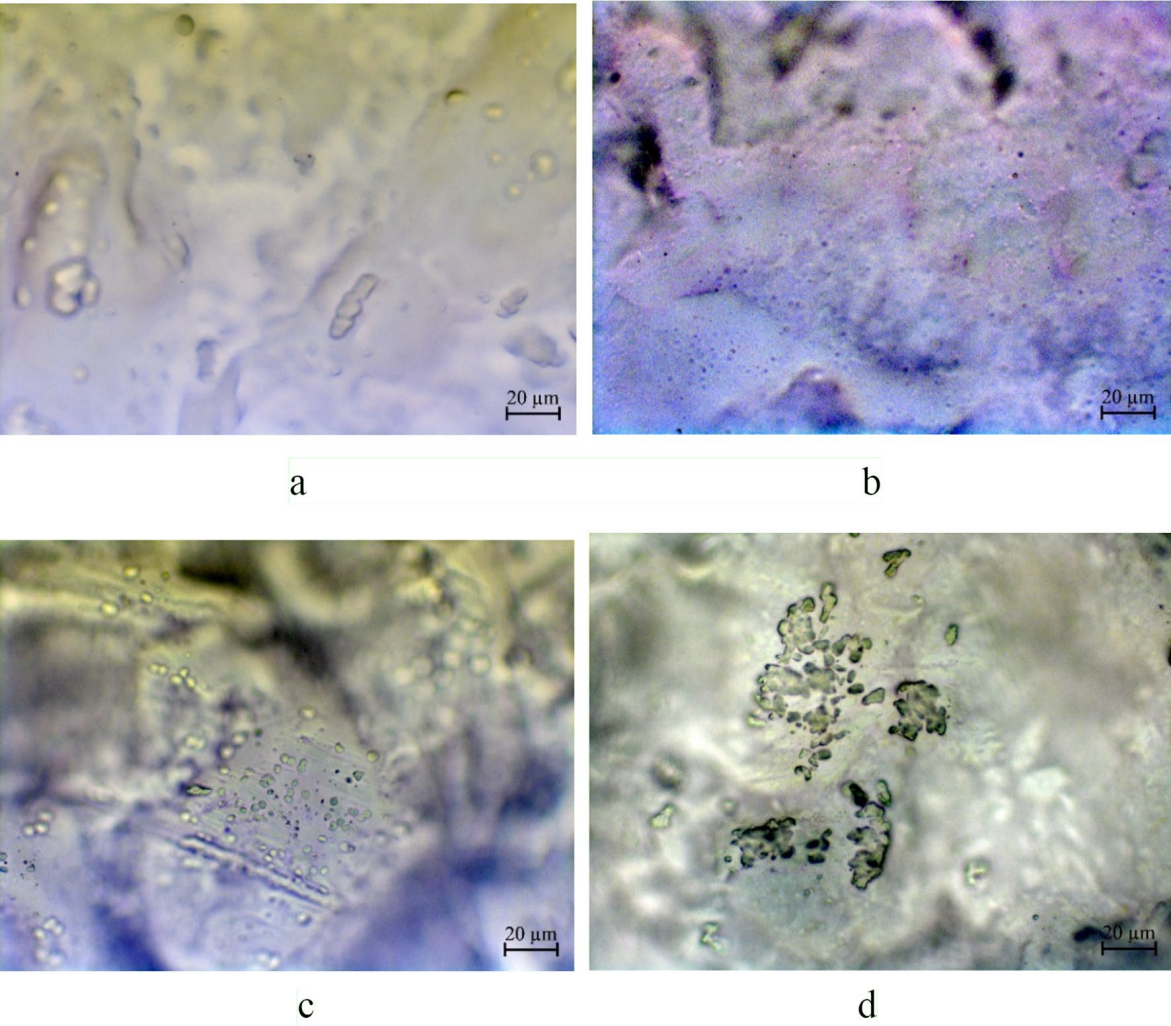
Micrographs of samples of methylcellulose films modified with CuO nanoparticles: (**a**) control, (**b**) 0,2%, (**c**) 0,4%, (**d**) 0.8%.

For the experiment we prepared conventional methylcellulose film as a control sample and methylcellulose films modified with 0.2%, 0.4% and 0.8% CuO nanoparticles as experimental samples. The use of these concentrations is associated with the results of a study of the fungicidal activity of CuO nanoparticles ( “Fungicidal activity of CuO nanoparticles stabilized with gelatin” Section). The concentration of nanoparticles in the solution that we used to prepare a methylcellulose film with 0.8% CuO nanoparticles was 2.5 · 10^−3^ mol/LSamples of methylcellulose films modified with CuO nanoparticles were studied by optical microscopy. The resulting micrographs are presented in Fig. 20.

Analysis of the obtained microphotographs showed that samples of methylcellulose films modified with CuO nanoparticles have a homogeneous structure. Figure 20b shows uniformly distributed particles ranging in size from 1 to 2 µm in film with 0.2% CuO nanoparticles. In a film with 0.4% CuO nanoparticles (Fig. 20c) we clearly obserbed clusters of microparticles which are agglomerates of CuO nanoparticles stabilized with gelatin. Micrograph of a film with 0.8% CuO nanoparticles (Fig. 20d) shows the the highest concentration of microparticles, which causes a more intense staining of the film (Figs. 1 and 2). The formation of aggregates ranging in size from 1 to 2 µm is associated with the aggregation of CuO nanoparticles, which is caused by the drying process of the material.

**Figure 21:**
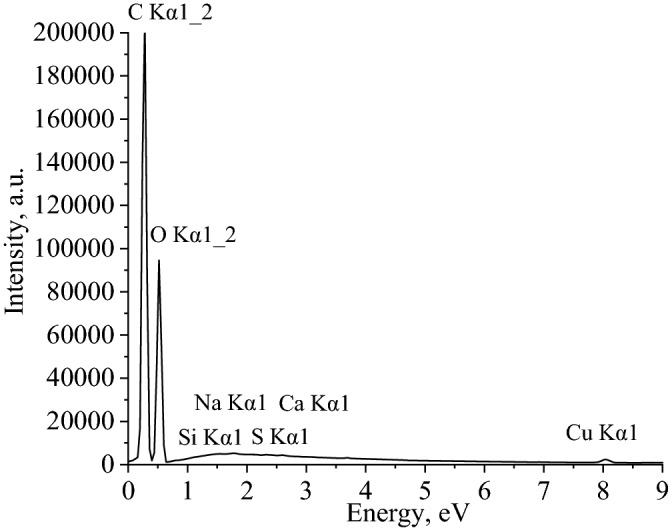
Energy-dispersive spectrum of methylcellulose film modified with 0.8% CuO nanoparticles.

**Figure 22:**
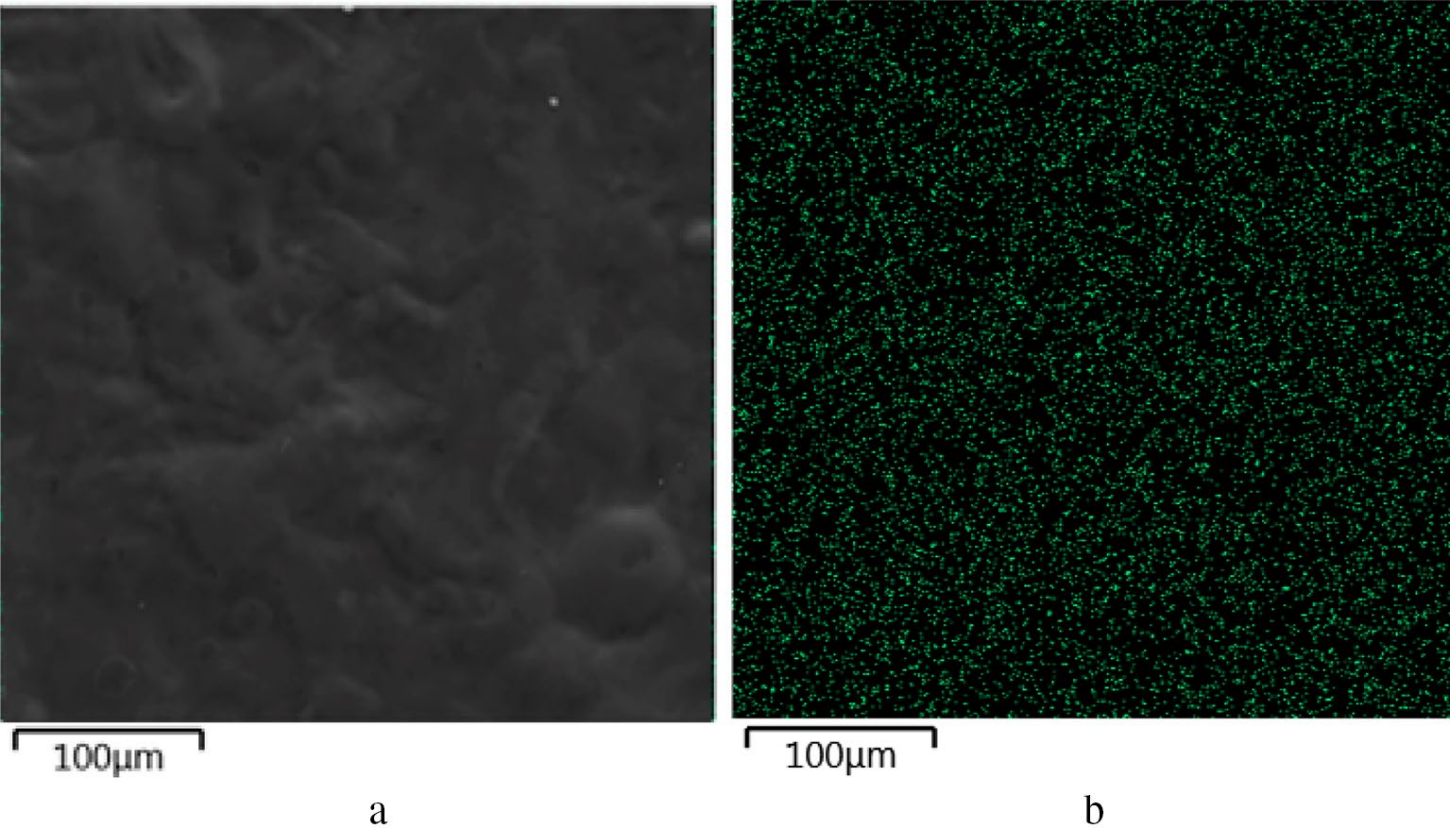
Results of elemental analysis of methylcellulose film modified with 0.8% CuO nanoparticles (**a**) SEM-micrography, (**b**) layer distribution map (Cu).

Next, we studied elemental composition of methylcellulose films using scanning electron microscope MIRA3-LMH with a system for determining the elemental composition AZtecEnergy Standard/X-max 20. The results of the analysis are presented in Figs. 21 and 22, Table 3 and Supplementary (Fig. S69–S71)..

**Table 3:** Decoding of energy-dispersive spectra of methylcellulose films modified with CuO nanoparticles.

Element	Mass fraction of the element, %			
	Control	0.2% CuO	0.4% CuO	0.8% CuO
C	52.88 ± 0.01	52.48 ± 0.01	50.17 ± 0.01	50.09 ± 0.01
O	47.04 ± 0.01	47.27 ± 0.01	49.47 ± 0.01	48.17 ± 0.01
Na	0.03 ± 0.01	0.02 ± 0.01	0.03 ± 0.01	0.03 ± 0.01
Si	0.01 ± 0.01	0.01 ± 0.01	0.01 ± 0.01	0.02 ± 0.01
S	0.02 ± 0.01	0.01 ± 0.01	0.02 ± 0.01	0.02 ± 0.01
Ca	0.03 ± 0.01	0.02 ± 0.01	0.01 ± 0.01	0.04 ± 0.01
Cu	–	0.18 ± 0.01	0.28 ± 0.01	0.54 ± 0.01

Elemental composition analysis showed that methylcellulose films contain insignificant amounts of Na, Si, S and Ca with equivalent content in all samples.Thus, these elements are contained only in the packaging material, but not in the prepared preparation of CuO nanoparticles.

It is important to note that the CuO nanoparticles are evenly distributed in the methylcellulose film samples. The copper concentration corresponds to the calculated values and increases proportionally with an increase in the concentration of CuO nanoparticles in film samples.

### Effect of methylcellulose films modified with CuO nanoparticles on quality and shelf life of cheese

At the next stage, we investigated the possibility of using methylcellulose films modified with *CuO* nanoparticles for cheese packaging. Hard cheese “Holland” was used as the object of the study.

In the experiment we determined titrated acidity, copper content and microbiological parameters in hard cheese “Holland” during 7 days of storage in a thermostat at 35 ± 1 °C. The results of the experiment are presented in Tables 4 and 5.

**Table 4:** Dynamics of changes in titratable acidity and copper content of cheese samples during storage.

Storage duration, days	Samples			
	Control	0.2% CuO	0.4% CuO	0.8% CuO
**Copper content, µg/mg**				
0 (initial)	0.52 ± 0,03			
1	0.53 ± 0,04	0.56 ± 0.02	0.55 ± 0.02	0.56 ± 0.03
3	0.52 ± 0,03	0.56 ± 0.03	0.57 ± 0.03	0.59 ± 0.04
5	0.53 ± 0,02	0.58 ± 0.02	0.59 ± 0.03	0.61 ± 0.05
7	0.53 ± 0,04	0.57 ± 0.03	0.58 ± 0.02	0.64 ± 0.03
**Titratable acidity, °T**				
0 (initial)	210 ± 5			
1	215 ± 2	216 ± 3	213 ± 4	212 ± 3
3	223 ± 3	222 ± 4	220 ± 3	218 ± 5
5	230 ± 4	231 ± 5	227 ± 2	226 ± 4
7	234 ± 2	235 ± 4	233 ± 5	231 ± 5

**Table 5:** Dynamics of changes in microbiological indexes of cheese samples during storage.

Storage duration, days	Samples			
	Control	0.2% CuO	0.4% CuO	0.8% CuO
**QMAFAM, CFU/g**				
0 (initial)	4.5*10^2^			
1	6.6*10^2^	5.4*10^2^	5.7*10^2^	5.5*10^2^
3	2.3*10^3^	8.7*10^2^	7.2*10^2^	6.1*10^2^
5	6.1*10^3^	2.6*10^2^	2.2*10^2^	9.3*10^2^
7	1.1*10^4^	7.1*10^2^	4.5*10^2^	1.4*10^2^
**Coliforms, log (CFU/g)**				
0 (initial)	0.95 ± 0.01			
1	1.07 ± 0.02	1.08 ± 0.03	0.98 ± 0.02	0.97 ± 0.03
3	1.44 ± 0.02	1.28 ± 0.05	1.11 ± 0.03	0.99 ± 0.04
5	2.41 ± 0.05	1.95 ± 0.06	1.75 ± 0.07	1.31 ± 0.04
7	5.56 ± 0.08	3.23 ± 0.03	2.93 ± 0.04	1.68 ± 0.06
**Yeast and mold, log (CFU/g)**				
0 (initial)	0.12 ± 0.01			
1	0.13 ± 0.02	0.13 ± 0.02	0.12 ± 0.02	0.12 ± 0.01
3	0.67 ± 0.03	0.35 ± 0.04	0.24 ± 0.06	0.16 ± 0.02
5	1.13 ± 0.05	0.72 ± 0.04	0.69 ± 0.03	0.31 ± 0.04
7	1.61 ± 0.07	1.29 ± 0.06	1.08 ± 0.08	0.70 ± 0.04

According to the data obtained, we found that during the processing of cheese samples in methylcellulose films modified with CuO nanoparticles, there was a change in the copper content, which indicates the presence of migration of CuO nanoparticles into the product from the film. Nevertheless, it is worth noting that the maximum change in the concentration of copper in the experimental samples was only 0.12 µg/mg, which is not a toxic concentration^27,28^. Moreover, the migration of CuO nanoparticles can be reduced under adequate conditions of cheese storage in the refrigerator at a temperature of 0–4 °C. However, this is a topic for further research. In general, the small value of migration of CuO nanoparticles confirms the high stability of the developed preparation.

A change in the value of titrated acidity in cheese during storage may indicate oxidative changes in fat, or the activity of lactic acid bacteria. During the experiment, we found that the titrated acidity value was slightly lower in the experimental samples than in the control sample. Nevertheless, according to the results of the experiment, we cannot form a statistically reliable trend due to very close values obtained. Therefore, based on the data, we can only draw one conclusion: CuO nanoparticles stabilized with gelatin are not characterized by sufficiently high antioxidant activity to reliably protect fat from oxidation during storage of experimental samples of cheese.

According to the results presented in Table 5, we found that methylcellulose films modified with CuO nanoparticles reduced the growth and development of QMAFAM, coliforms, yeast and mold in experimental cheese samples, compared with a control cheese sample packed in conventional methylcellulose films. The data obtained correlate with our data on the fungicidal activity of CuO nanoparticles stabilized with gelatin, as well as with the data of other authors who have studied the antibacterial activity of CuO nanoparticles^2,10,31,36,37^.

Thus, the results of our experiment indicate that the CuO nanoparticles stabilized with gelatin have a high potential for use in food packaging – both as an independent nanofilm and as part of other packaging materials.

## Conclusions

In the present work, a technique for the synthesis of copper oxide nanoparticles stabilized with gelatin was developed. Our results showed that using only copper acetate as a precursor allows the obtaining of monophase copper (II) oxide. According to the photon correlation spectroscopy data, copper oxide nanoparticles stabilized with gelatin in the aquatic medium had a monomodal size distribution with an average hydrodynamic radius of 61 nm. The study of the pH effect on the colloidal stability showed that the sample was stable in the pH range of 6.8 to 11.98. Consequenty, a model for the stabilization of CuO nanoparticles with gelatin was proposed. It was supposed that stabilization occurs due to the interaction of CuO nanoparticles with the hydroxyl groups of gelatin. This was confirmed by the IR spectroscopy data. The effect of the ionic strength of the solution on the stability of CuO nanoparticles was also studied, and was found that Ca^2+^ ions had the greatest impact on the samples' colloidal stability.

It was found that CuO nanoparticles stabilized with gelatin have a fungicidal activity at concentration equivalent to 2.5 · 10^−3^ mol/L and as a material for food nanopackaging can provide an increase in the shelf life of products as presented by the example of strawberries and tomatoes. High level of stability of CuO nanoparticles stabilized with gelatin also would support its use in the creation of active food packaging materials.

We investigated the possibility of using methylcellulose films modified with CuO nanoparticles for packaging and storage of hard cheese “Holland”. The distribution of CuO nanoparticles in the methylcellulose film was uniform. We found that methylcellulose films modified with CuO nanoparticles inhibited the growth and development of QMAFAM, coliforms, yeast and mold in experimental cheese samples. Our research has shown that during the cheese storage in thermostat at 35 ± 1 °C for 7 days, CuO nanoparticles migrated to the product from the film. Nevertheless, it is worth noting that the maximum change in the concentration of copper in the experimental samples was only 0.12 µg/mg, which is not a toxic concentration. Moreover, the migration of CuO nanoparticles can be reduced under adequate conditions of cheese storage in the refrigerator at a temperature of 0–4 °C. However, this is a topic for further research. In general, the small value of migration of CuO nanoparticles confirms the high stability of the developed preparation.

Thus, the results of our experiment indicate that the CuO nanoparticles stabilized with gelatin have a high potential for use in food packaging – both as an independent nanofilm and as part of other packaging materials.

## Supplementary Information

Supplementary Information.

## Data Availability

All raw and analyzed data as well as the materials are available in this study. Additional data that support the findings of this study are available from corresponding authors upon request.
