# Supplementary material for: Synthesis of CuO nanoparticles stabilized with gelatin for potential use in food packaging applications
Source: Sci Rep. 2022 Jul 27;12:12843. doi: 10.1038/s41598-022-16878-w (PMC9334594; doi:10.1038/s41598-022-16878-w)
Supplement: Supplementary file 1 — Supplementary Information. [file 41598_2022_16878_MOESM1_ESM.docx]

| 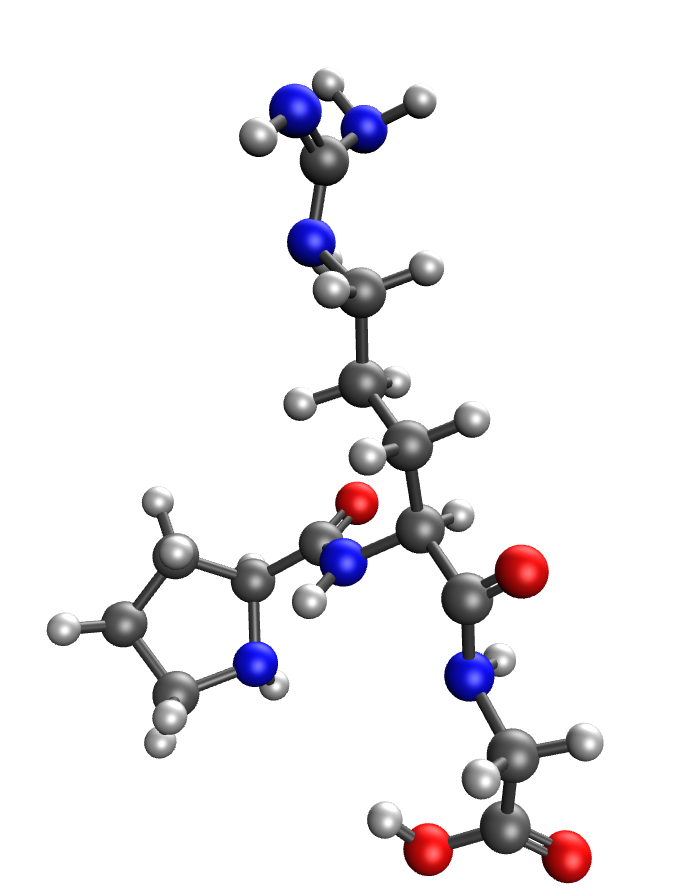 | 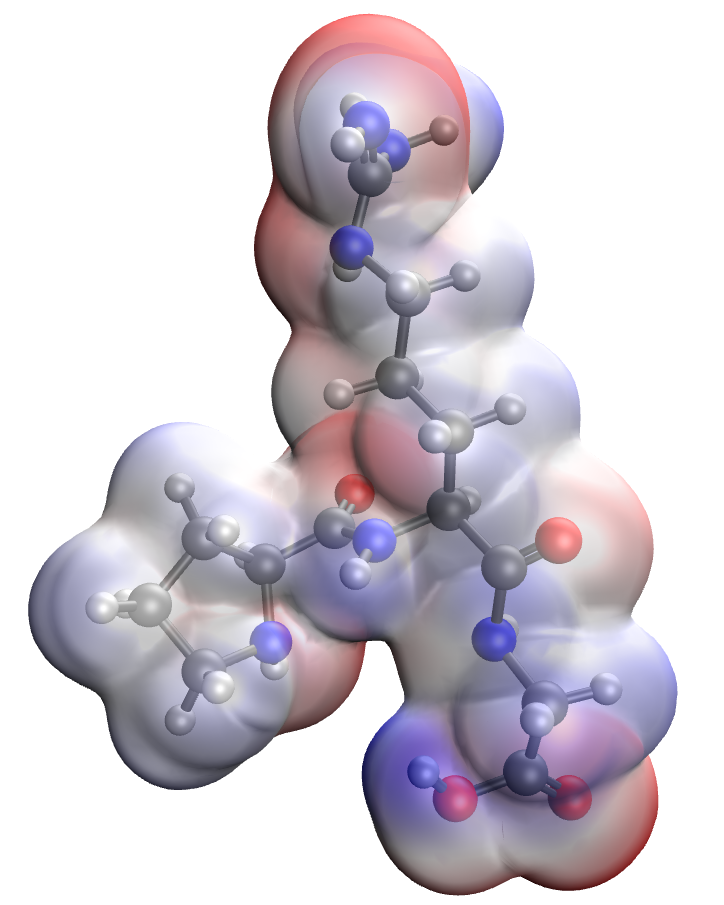 |
| --- | --- |
| a | b |
| 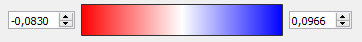 | |
| c | |
| 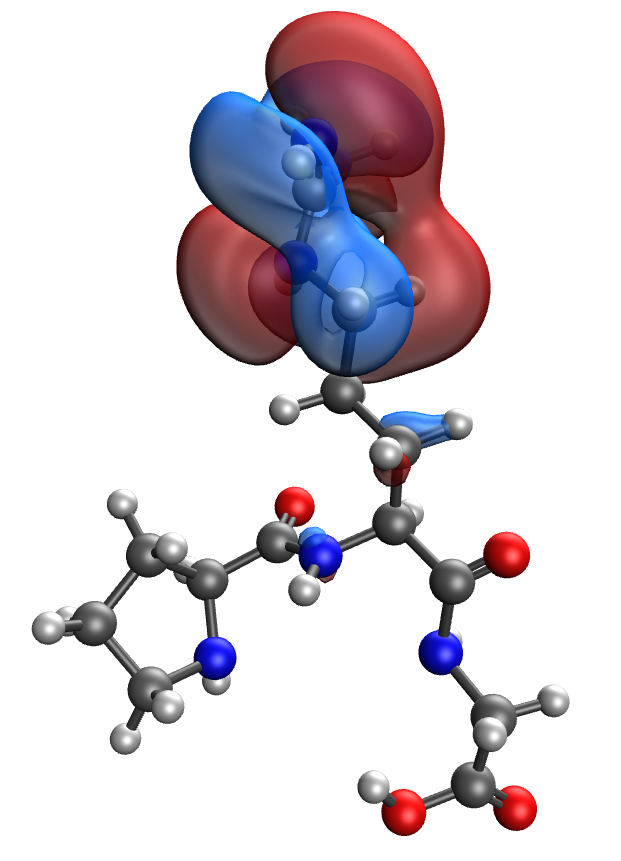 | 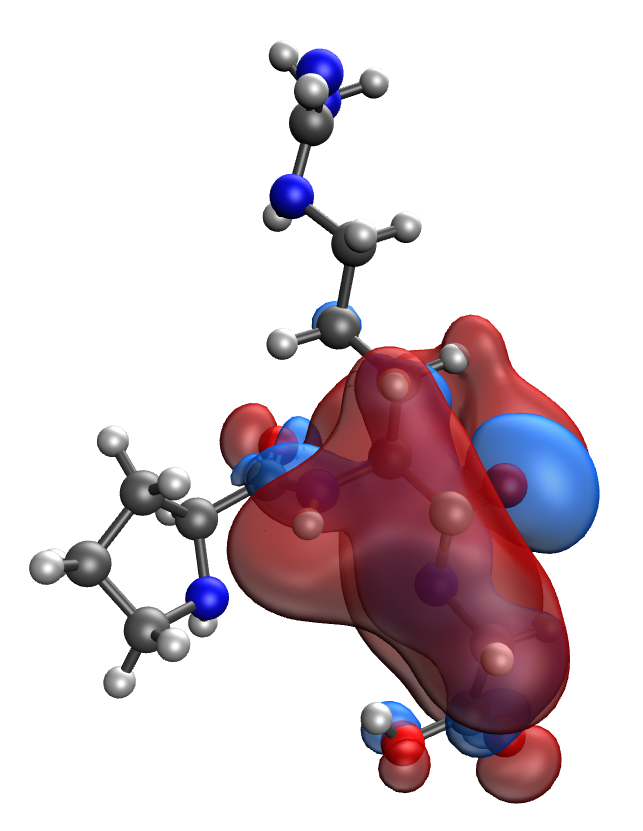 |
| d | e |

Fig. S1. Results of the quantum-chemical simulation of the *Pro-Arg-Gly* gelatin segment: a model of the molecular complex (a), the distribution of electron density (b), electronic density distribution gradient (c), HOMO (d), LUMO (e)

| 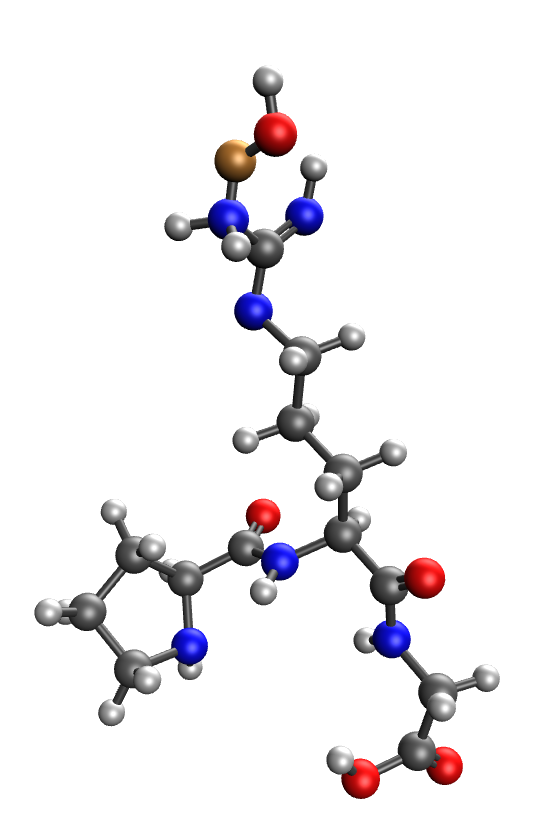 | 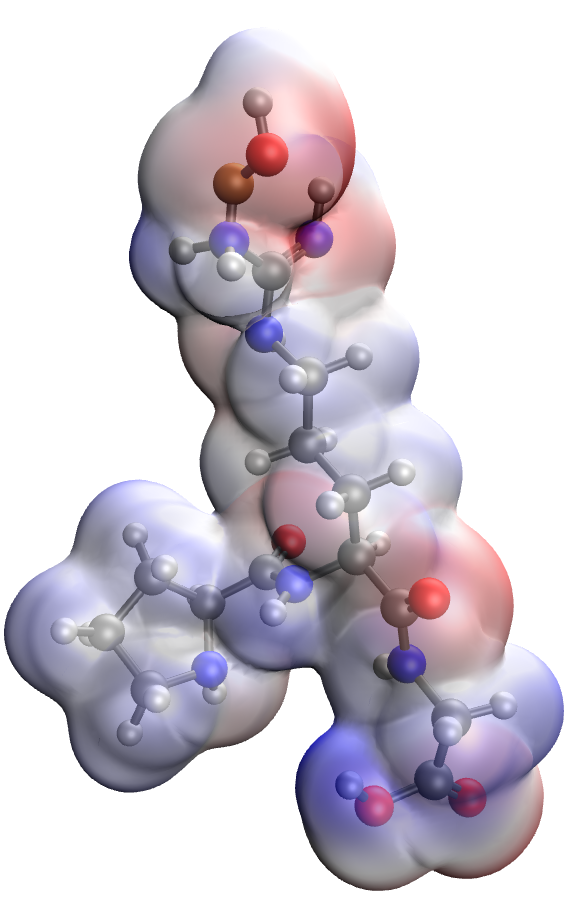 |
| --- | --- |
| a | b |
| 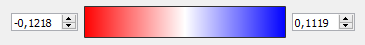 | |
| c | |
| 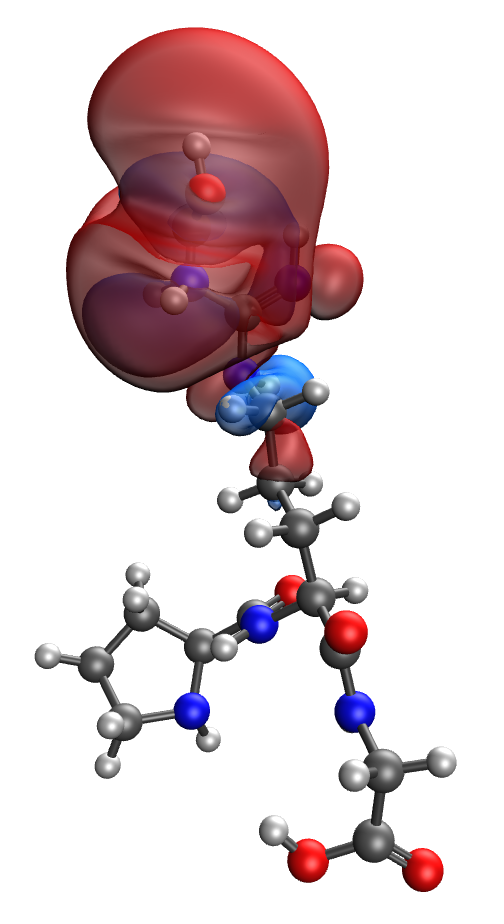 | 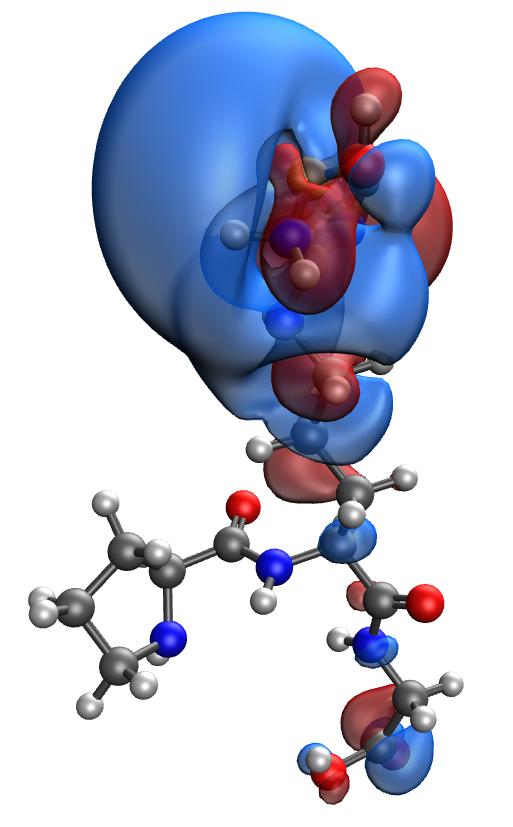 |
| d | e |

Fig. S2. Results of the quantum-chemical simulation of the *Pro-Arg-Gly* gelatin segment, bonded with CuO molecule: a model of the molecular complex (a), the distribution of electron density (b), electronic density distribution gradient (c), HOMO (d), LUMO (e)

| 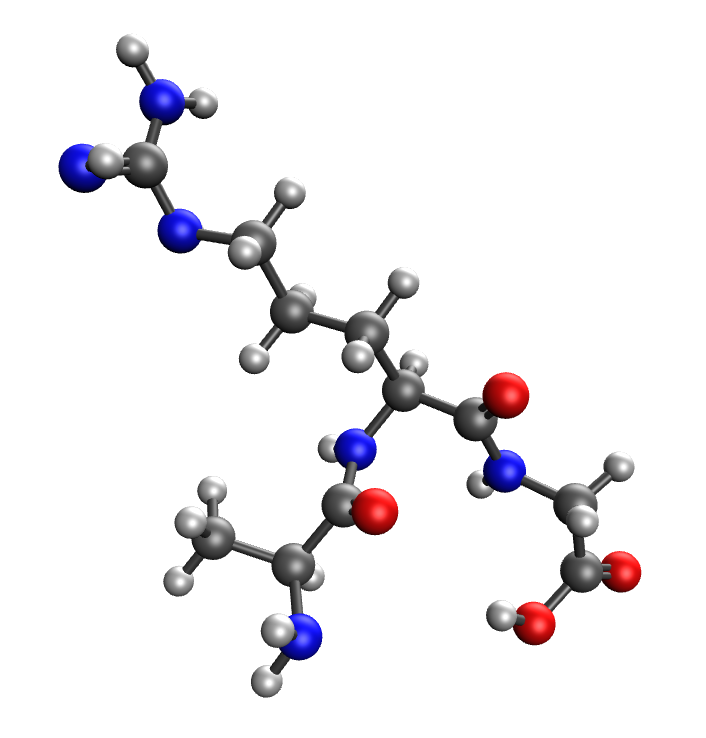 | 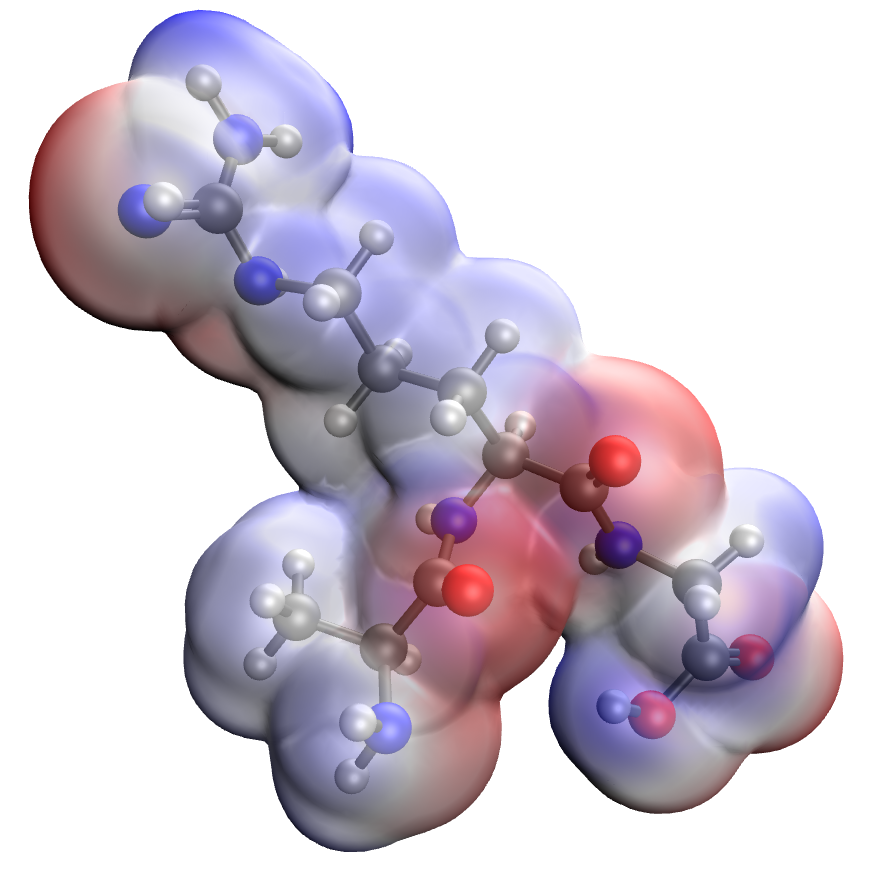 |
| --- | --- |
| a | b |
| 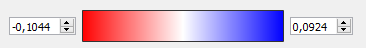 | |
| c | |
| 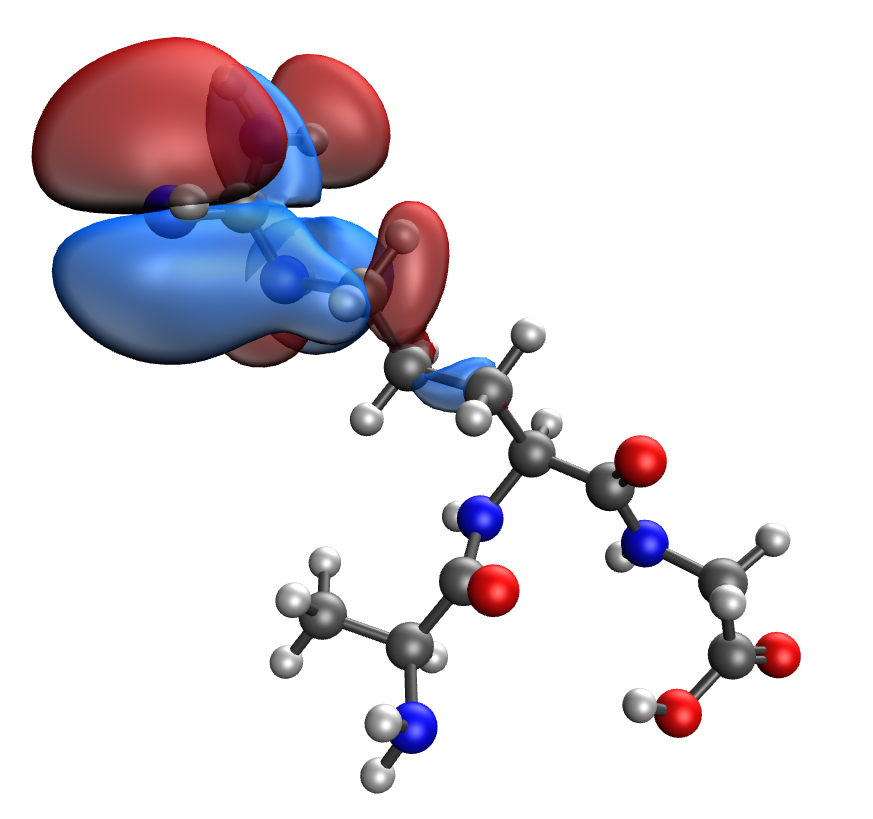 | 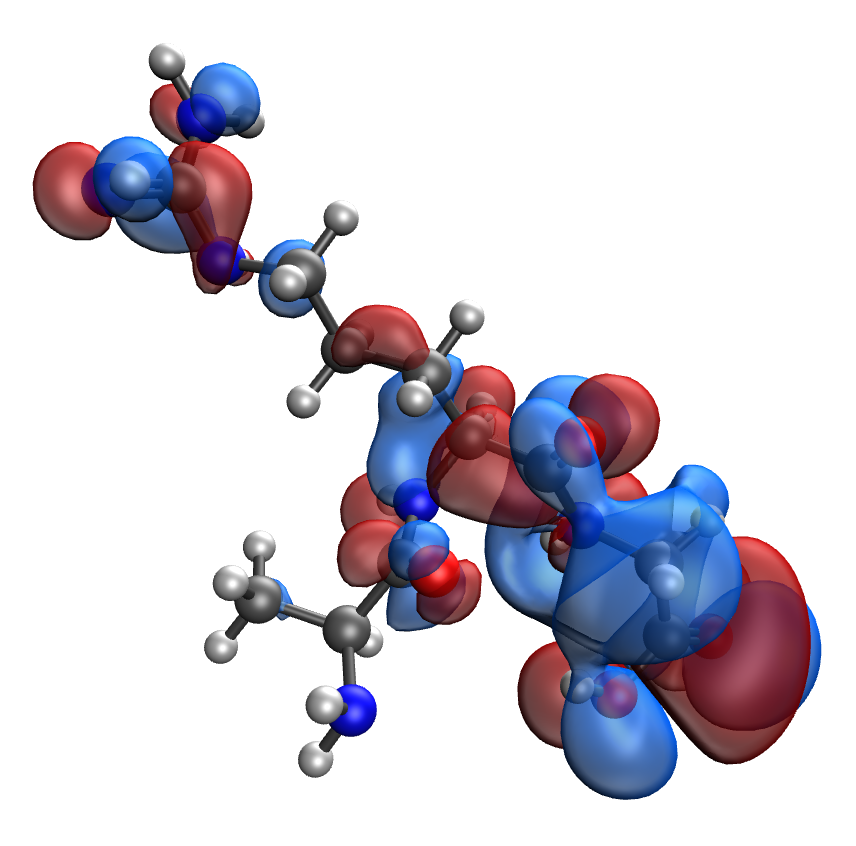 |
| d | e |

Fig. S3. Results of the quantum-chemical simulation of the *Ala-Arg-Gly* gelatin segment: a model of the molecular complex (a), the distribution of electron density (b), electronic density distribution gradient (c), HOMO (d), LUMO (e)

| 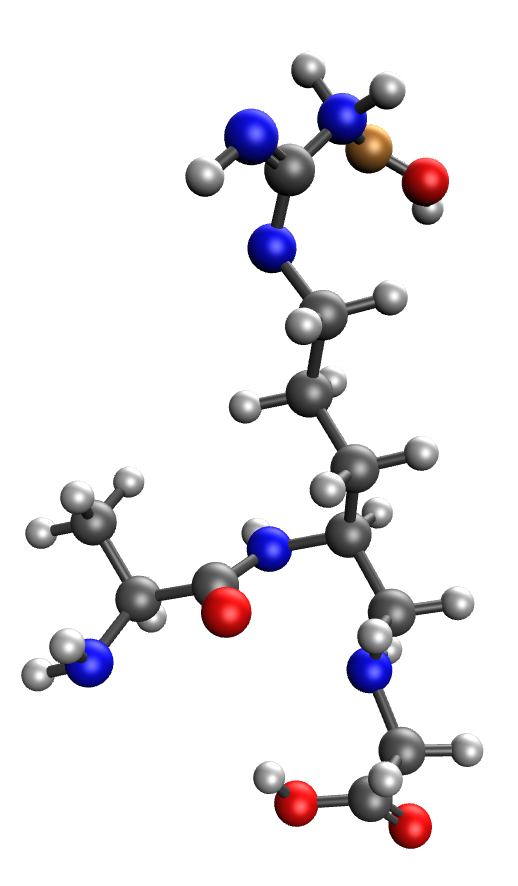 | 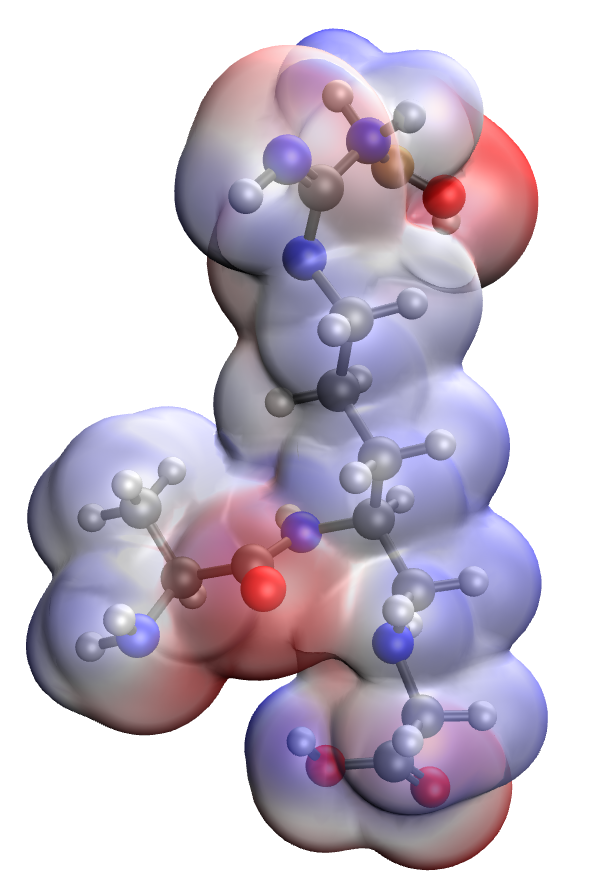 |
| --- | --- |
| a | b |
| 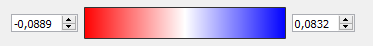 | |
| c | |
| 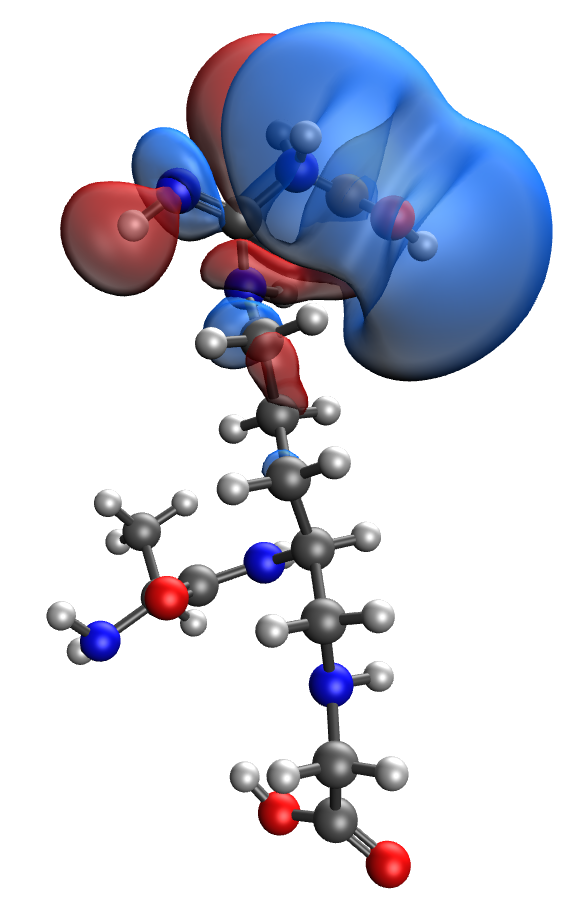 | 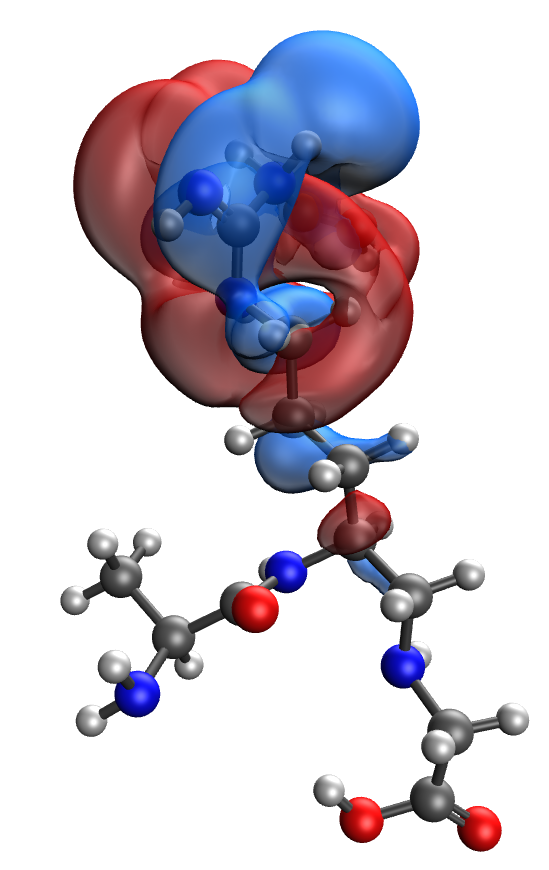 |
| d | e |

Fig. S4. Results of the quantum-chemical simulation of the *Ala-Arg-Gly* gelatin segment, bonded with CuO molecule: a model of the molecular complex (a), the distribution of electron density (b), electronic density distribution gradient (c), HOMO (d), LUMO (e)

| 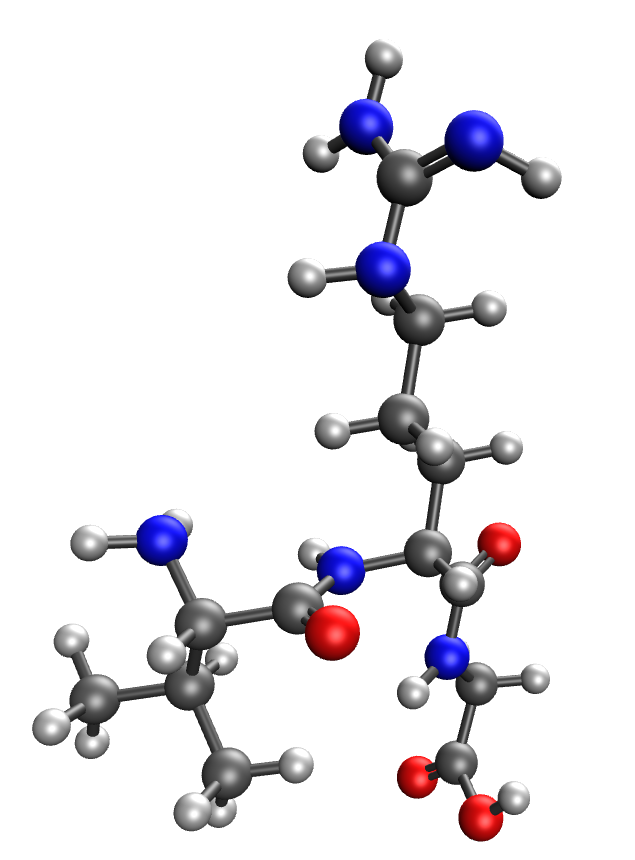 | 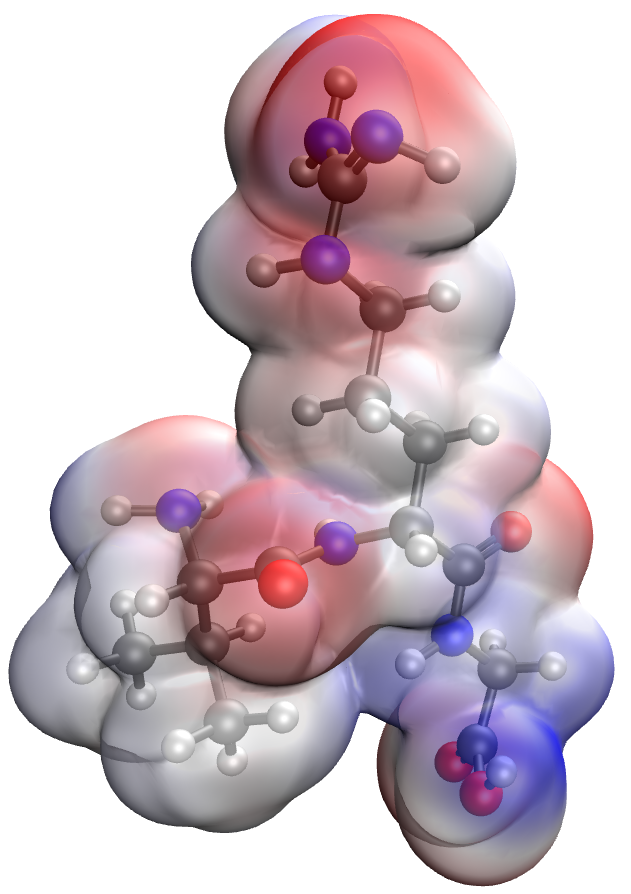 |
| --- | --- |
| a | b |
| 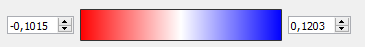 | |
| c | |
| 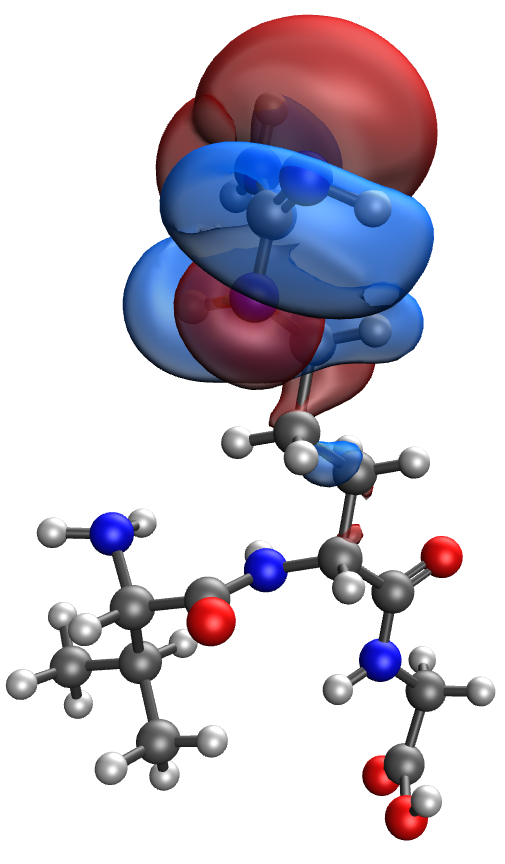 | 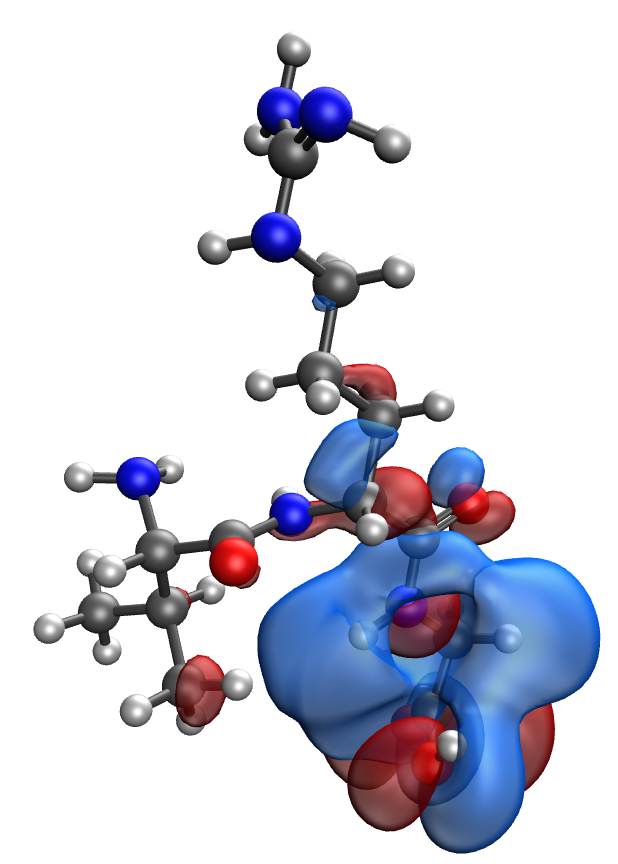 |
| d | e |

Fig. S5. Results of the quantum-chemical simulation of the Val*-Arg-Gly* gelatin segment: a model of the molecular complex (a), the distribution of electron density (b), electronic density distribution gradient (c), HOMO (d), LUMO (e)

| 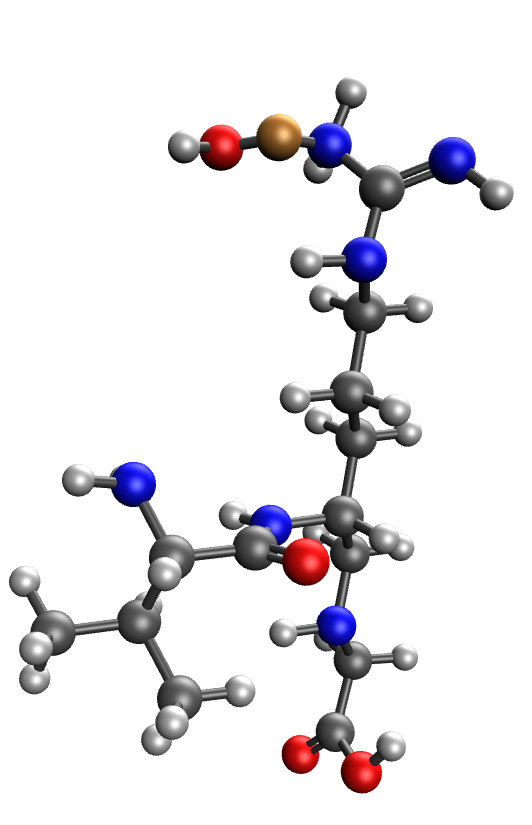 | 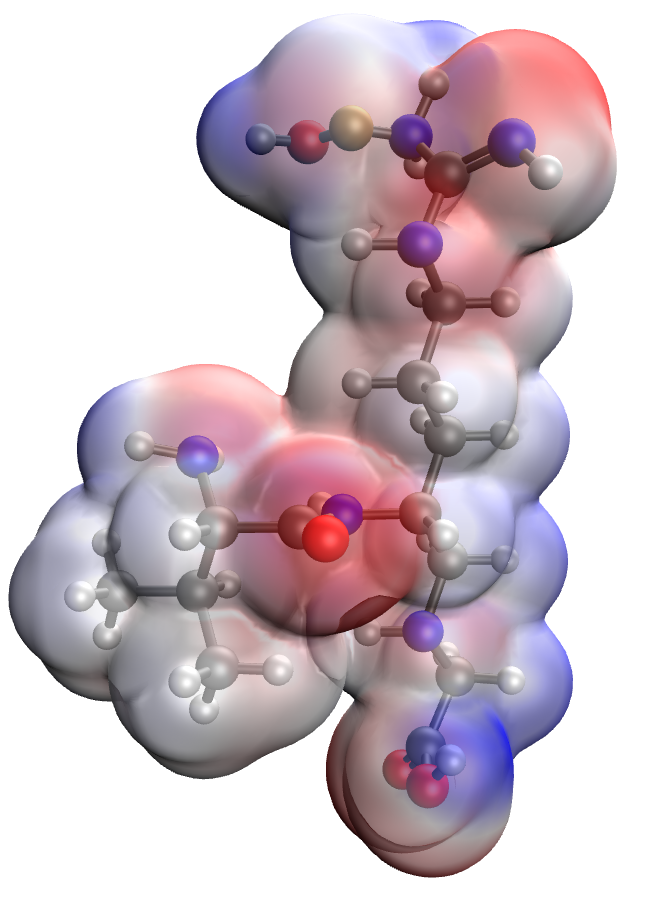 |
| --- | --- |
| a | b |
| 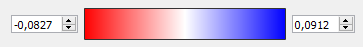 | |
| c | |
| 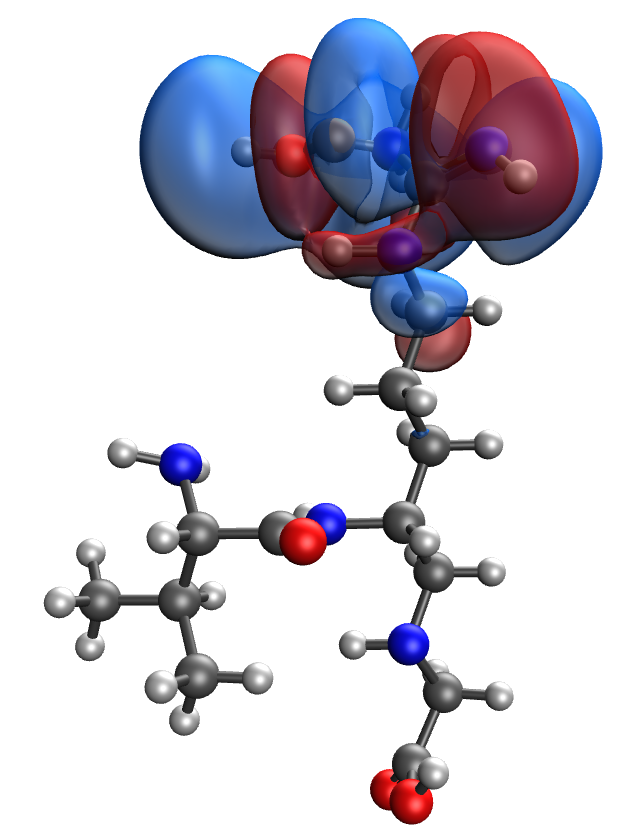 | 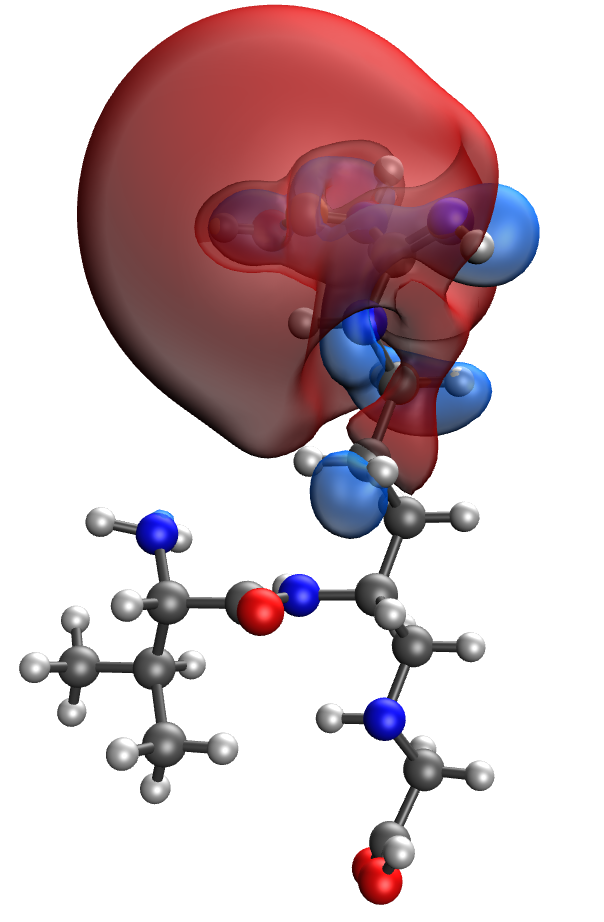 |
| d | e |

Fig. S6. Results of the quantum-chemical simulation of the *Val-Arg-Gly* gelatin segment, bonded with CuO molecule: a model of the molecular complex (a), the distribution of electron density (b), electronic density distribution gradient (c), HOMO (d), LUMO (e)

| 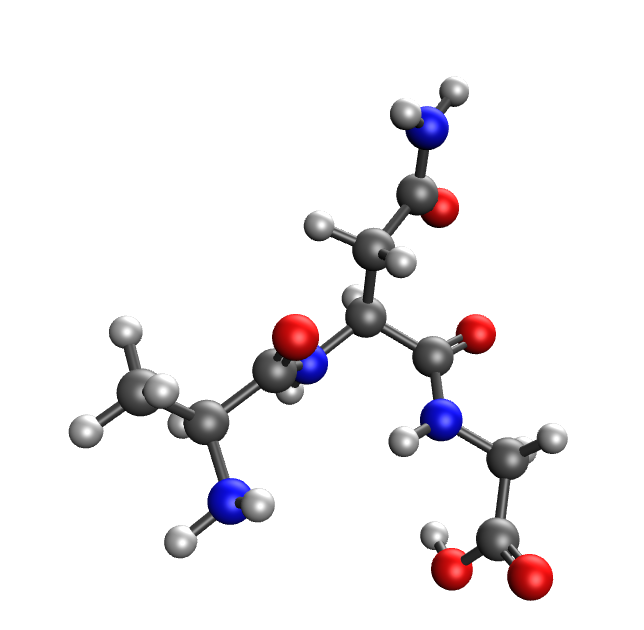 | 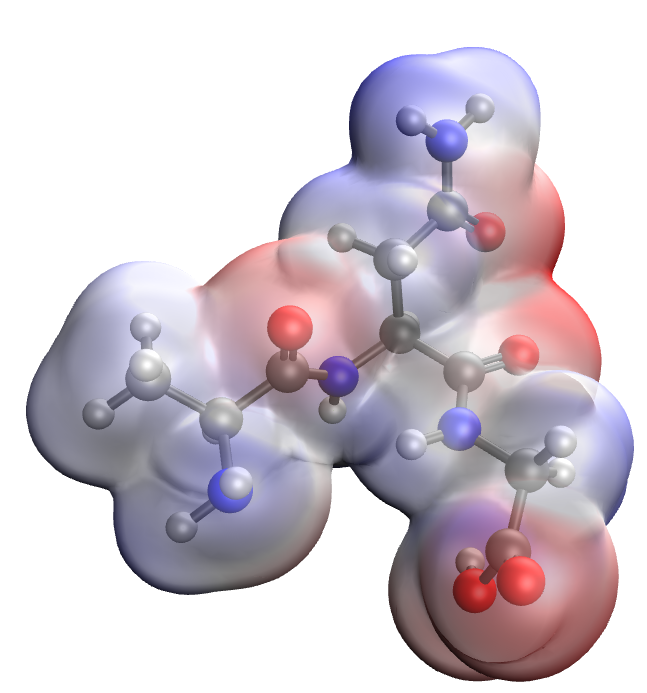 |
| --- | --- |
| a | b |
| 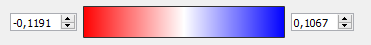 | |
| c | |
| 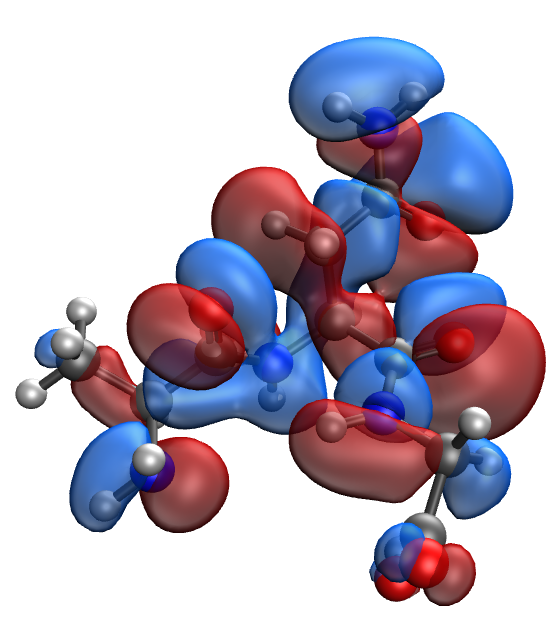 | 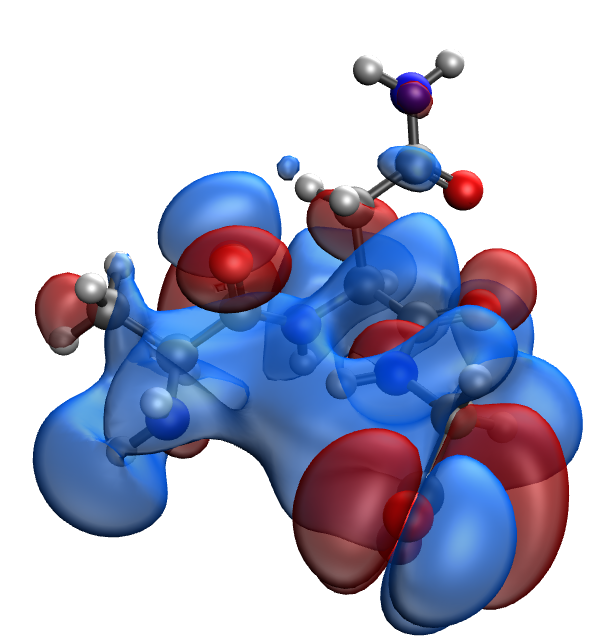 |
| d | e |

Fig. S7. Results of the quantum-chemical simulation of the Ala*-Asn-Gly* gelatin segment: a model of the molecular complex (a), the distribution of electron density (b), electronic density distribution gradient (c), HOMO (d), LUMO (e)

| 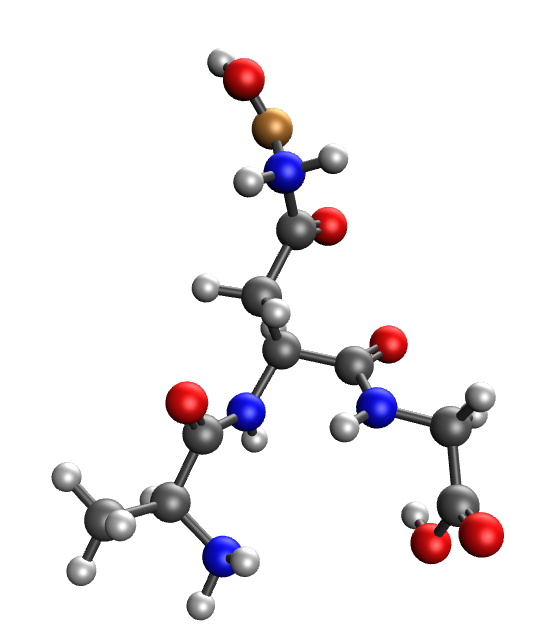 | 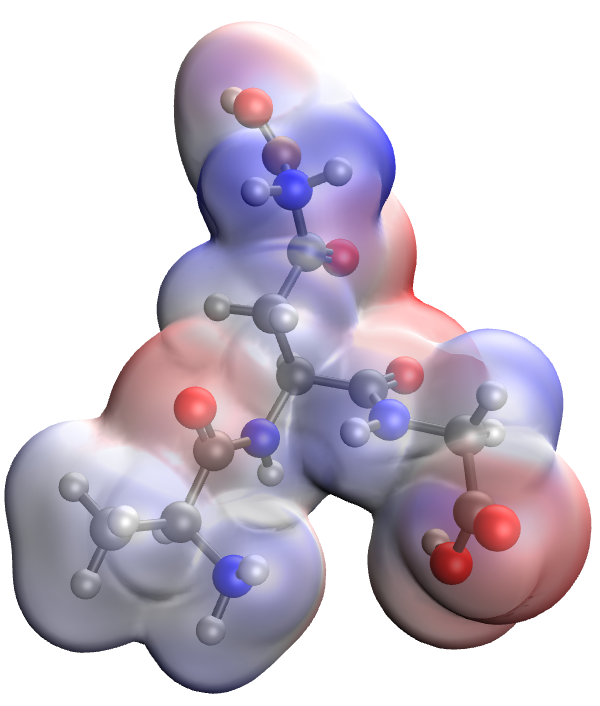 |
| --- | --- |
| a | b |
| 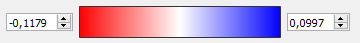 | |
| c | |
| 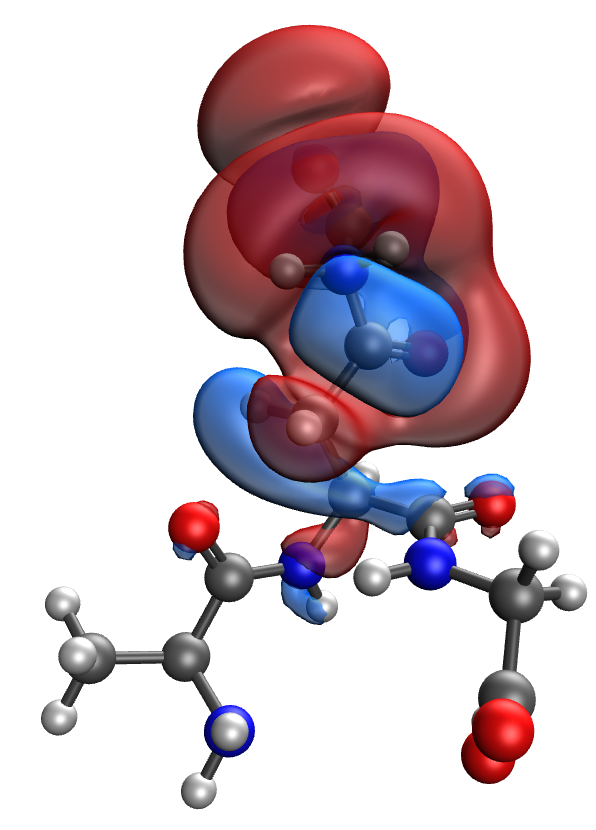 | 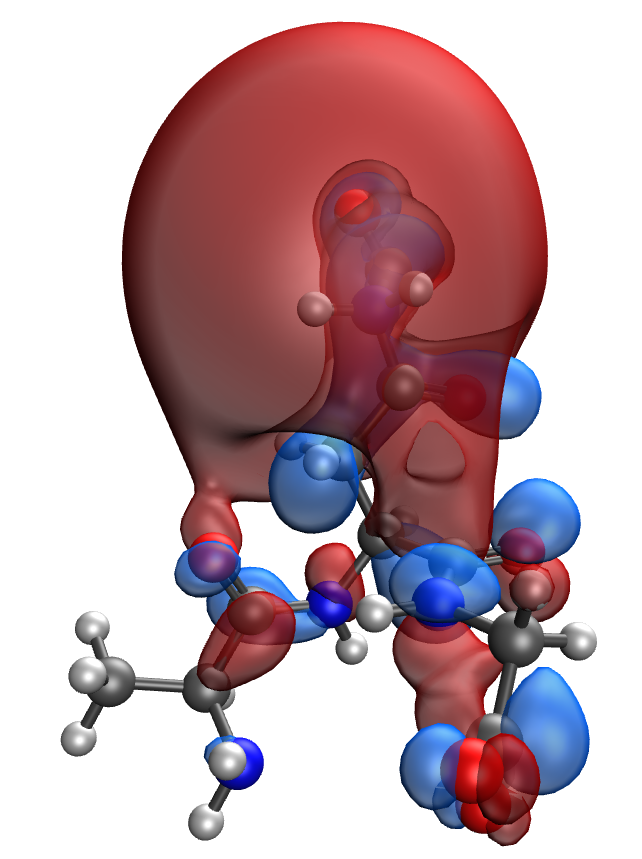 |
| d | e |

Fig. S8. Results of the quantum-chemical simulation of the *Ala-Asn-Gly* gelatin segment, bonded with CuO molecule: a model of the molecular complex (a), the distribution of electron density (b), electronic density distribution gradient (c), HOMO (d), LUMO (e)

| 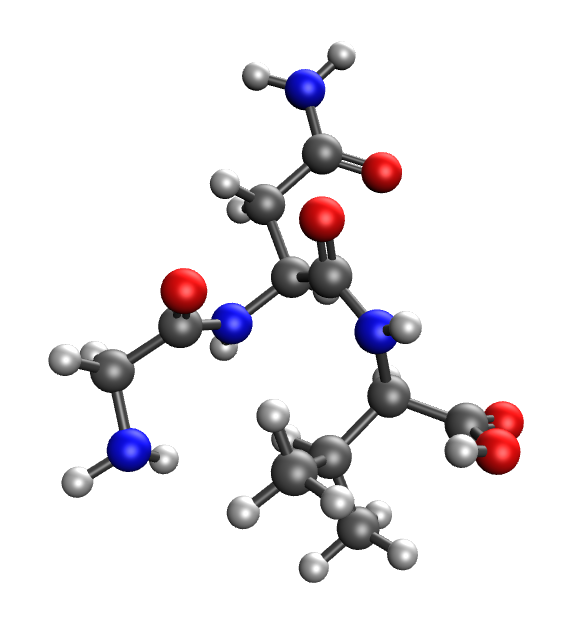 | 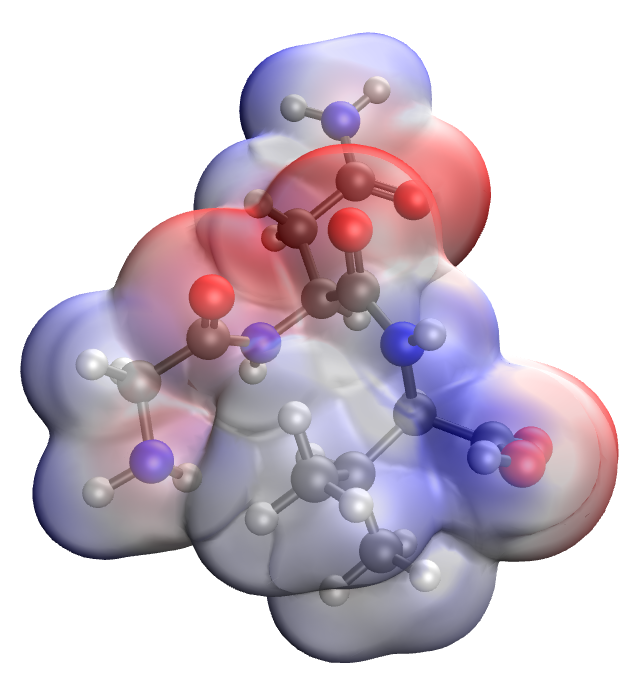 |
| --- | --- |
| a | b |
| 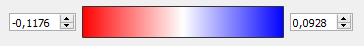 | |
| c | |
| 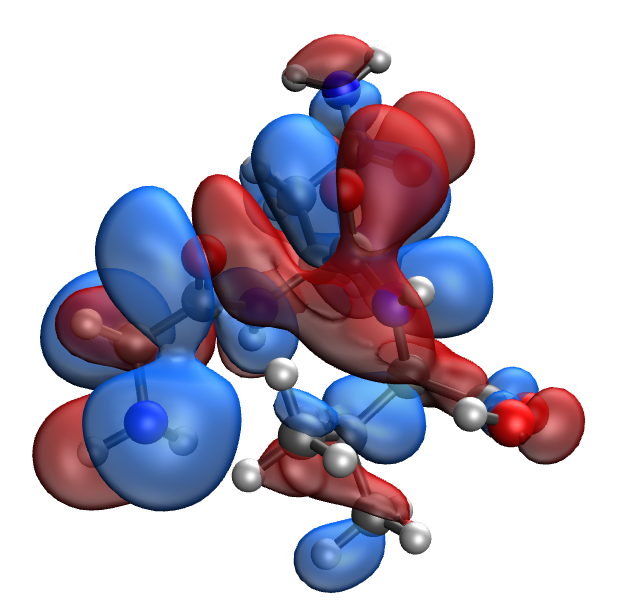 | 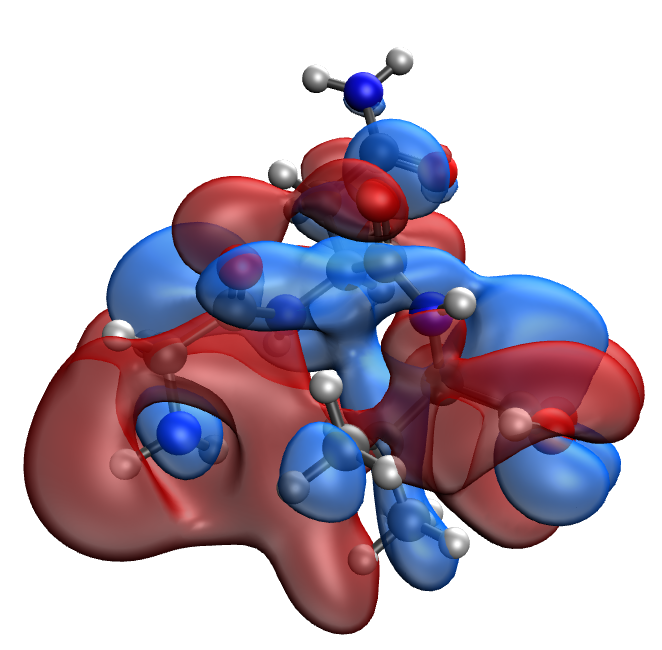 |
| d | e |

Fig. S9. Results of the quantum-chemical simulation of the Gly*-Asn-Val* gelatin segment: a model of the molecular complex (a), the distribution of electron density (b), electronic density distribution gradient (c), HOMO (d), LUMO (e)

| 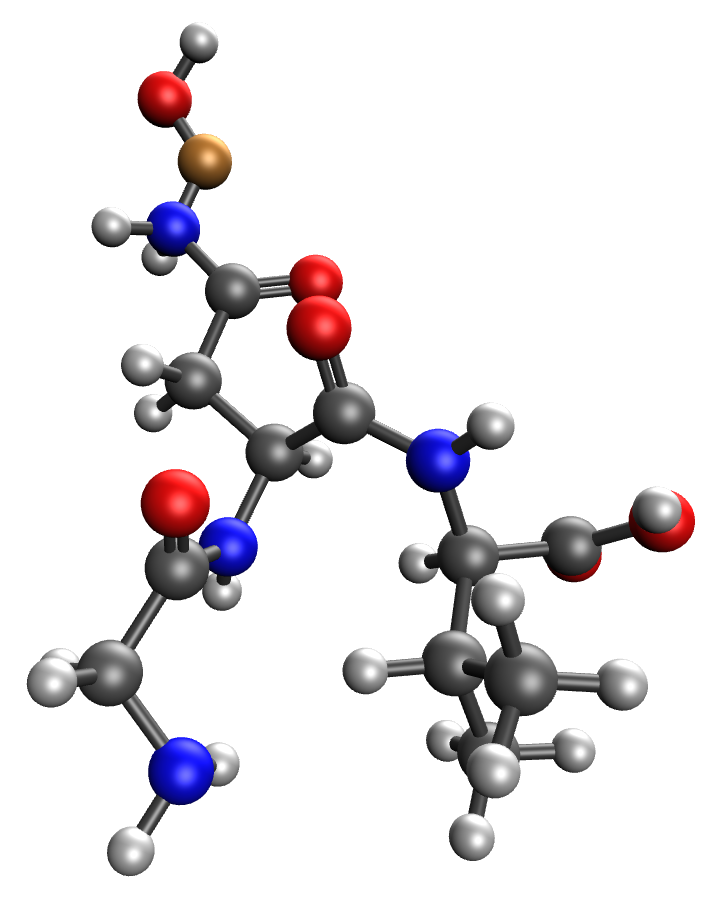 | 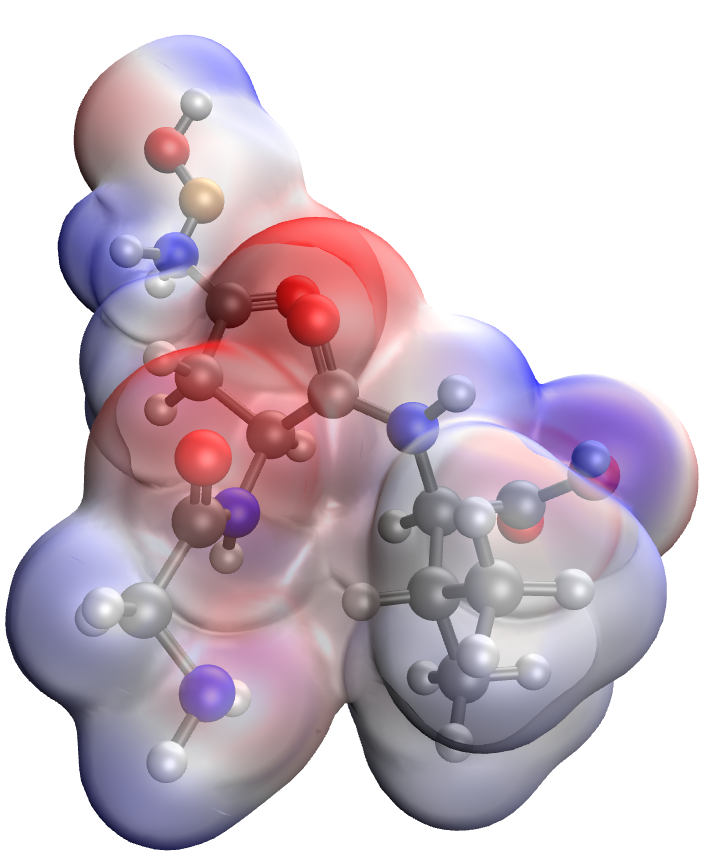 |
| --- | --- |
| a | b |
| 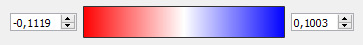 | |
| c | |
| 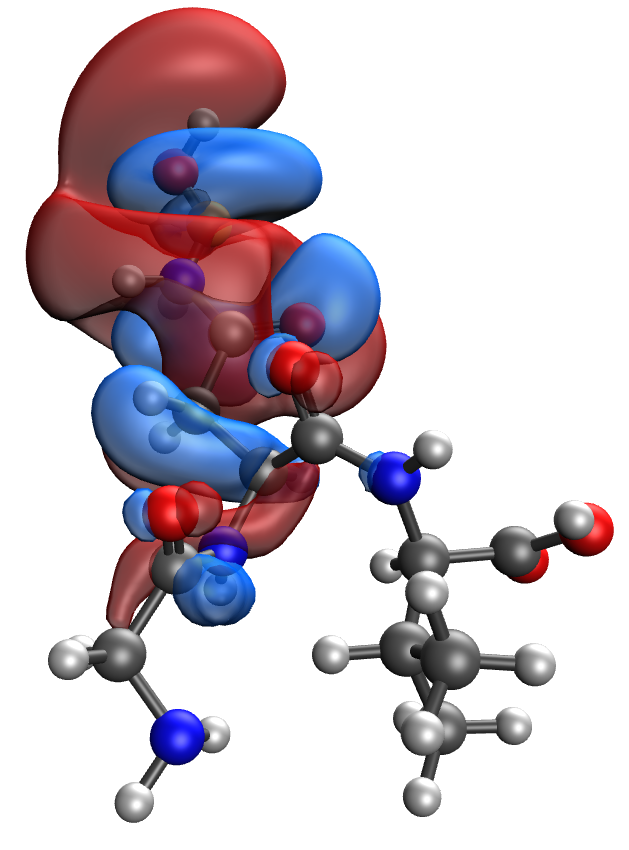 | 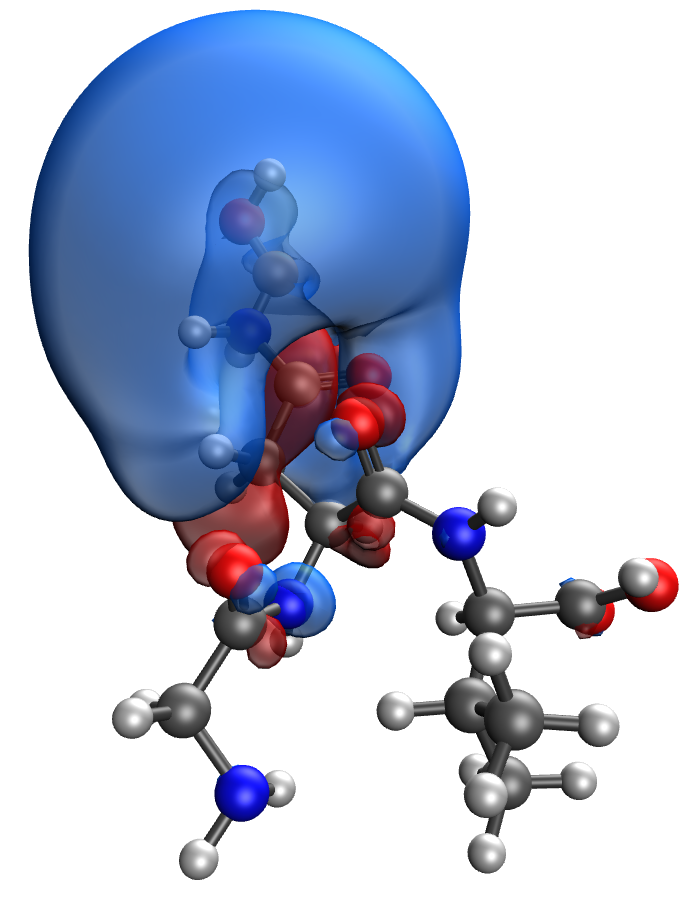 |
| d | e |

Fig. S10. Results of the quantum-chemical simulation of the *Gly-Asn-Val* gelatin segment, bonded with CuO molecule: a model of the molecular complex (a), the distribution of electron density (b), electronic density distribution gradient (c), HOMO (d), LUMO (e)

| 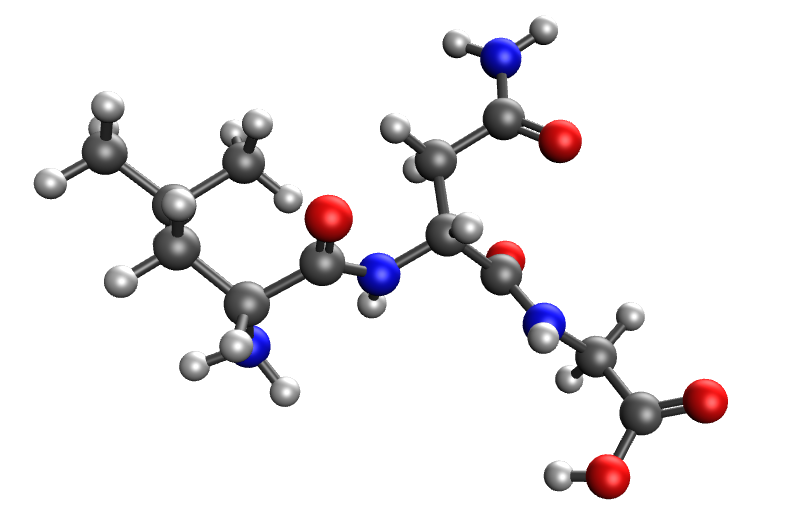 | 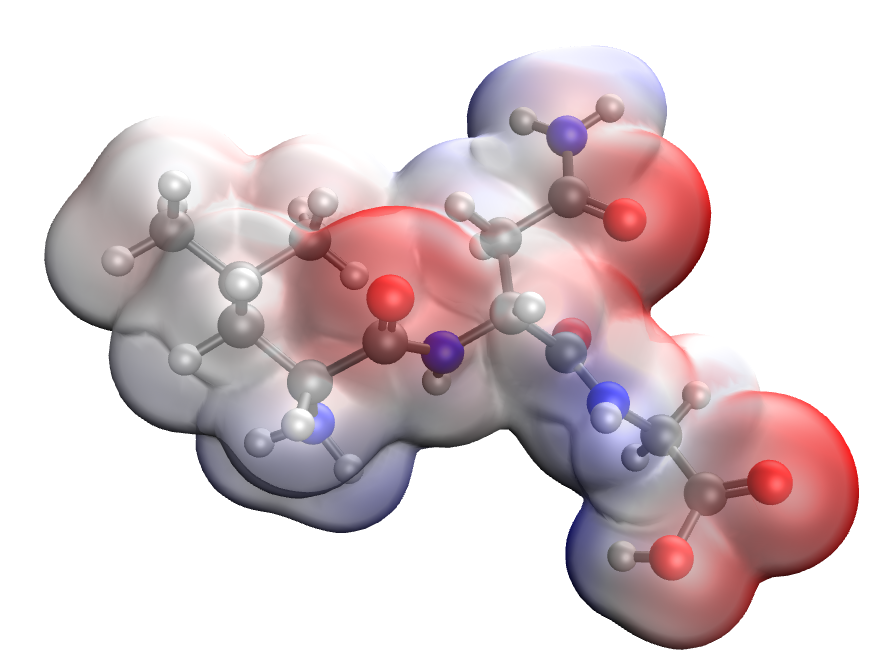 |
| --- | --- |
| a | b |
| 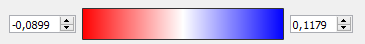 | |
| c | |
| 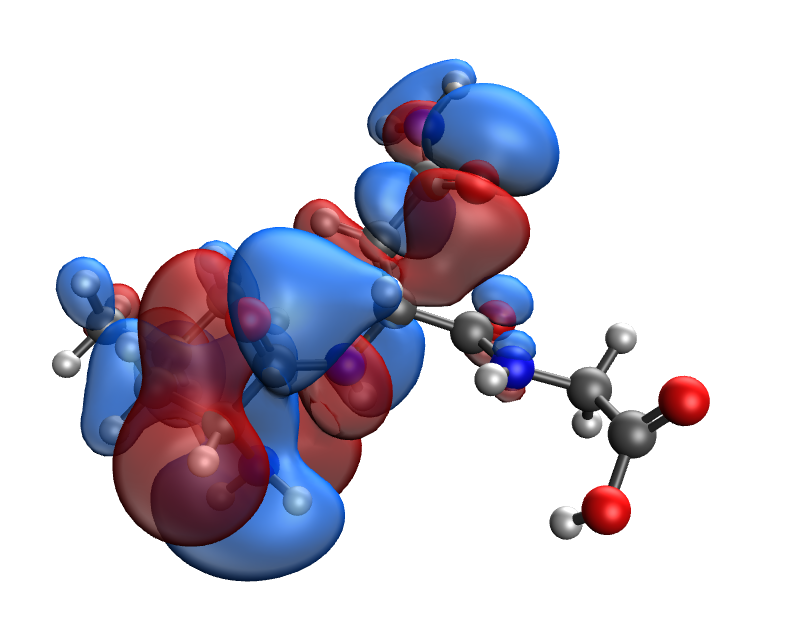 | 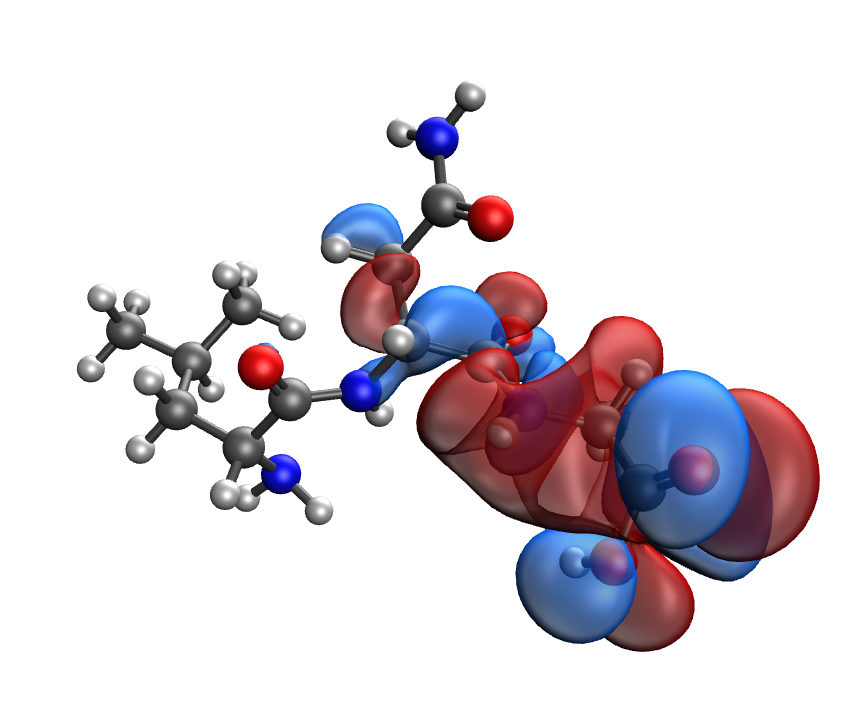 |
| d | e |

Fig. S11. Results of the quantum-chemical simulation of the Leu*-Asn-Gly* gelatin segment: a model of the molecular complex (a), the distribution of electron density (b), electronic density distribution gradient (c), HOMO (d), LUMO (e)

| 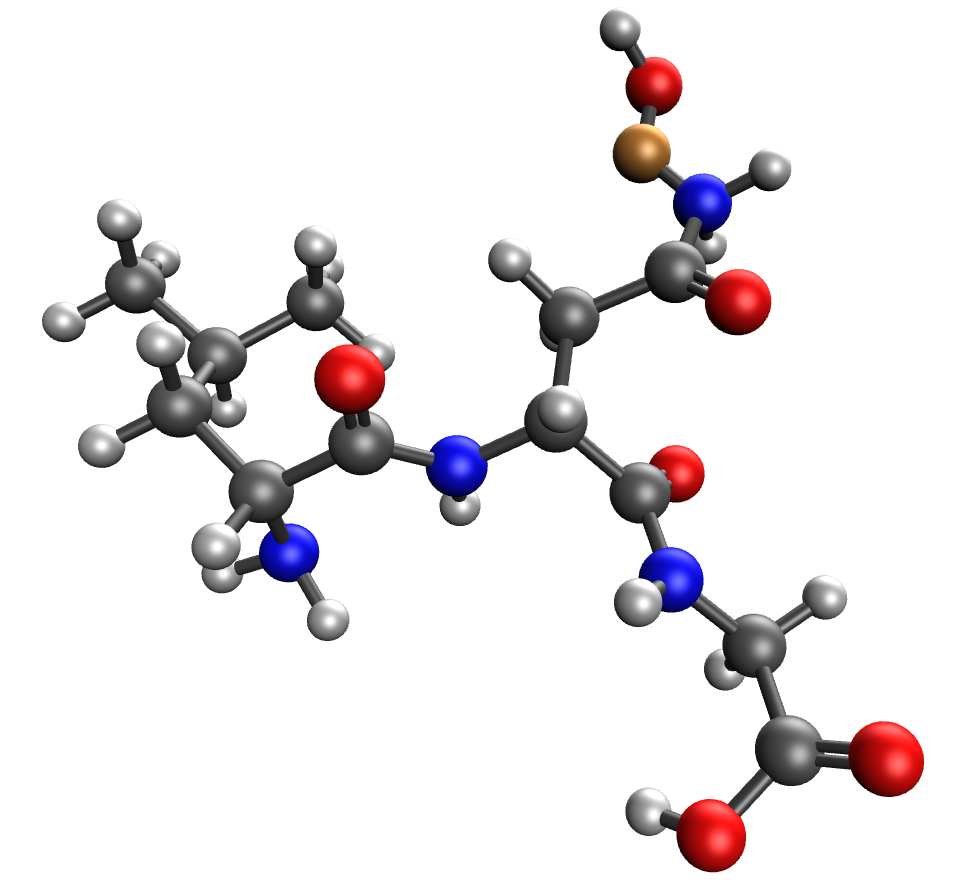 | 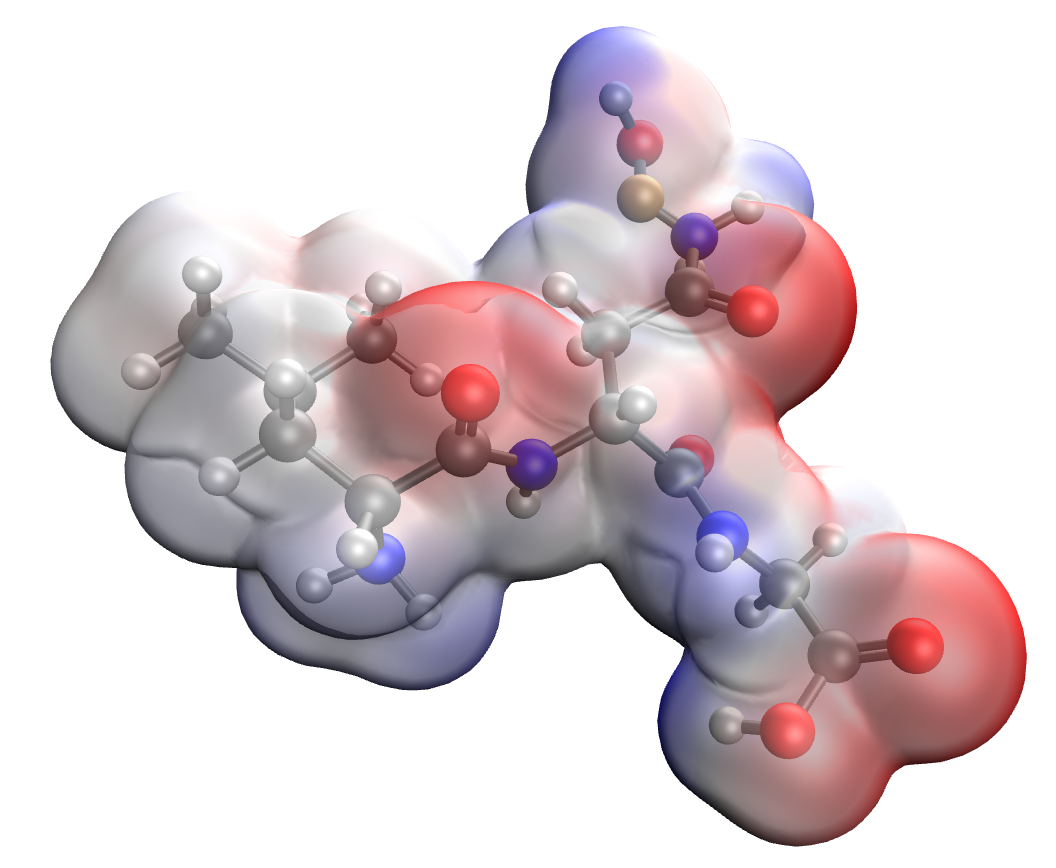 |
| --- | --- |
| a | b |
| 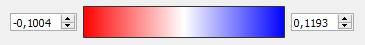 | |
| c | |
| 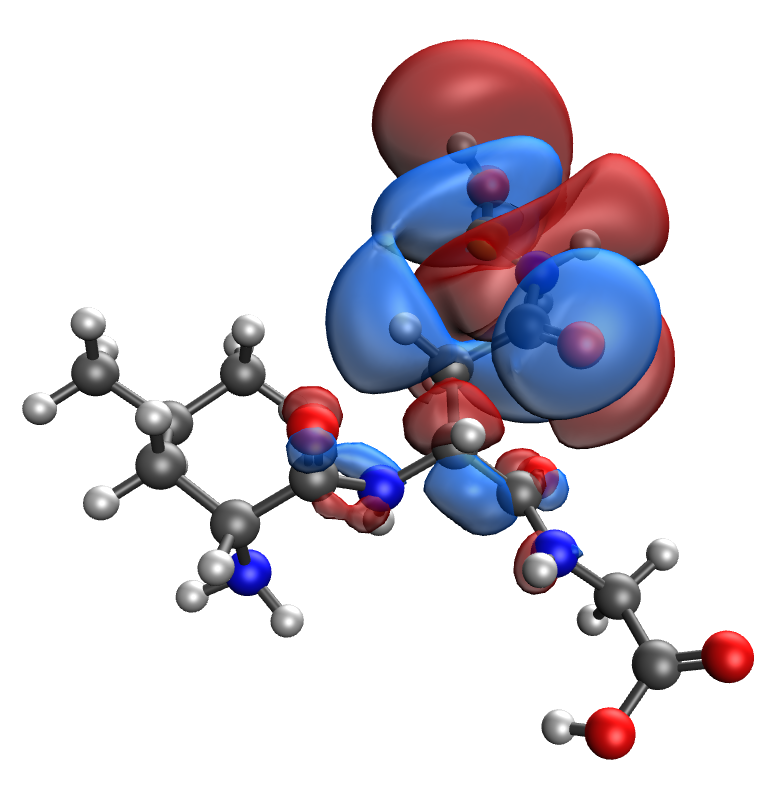 | 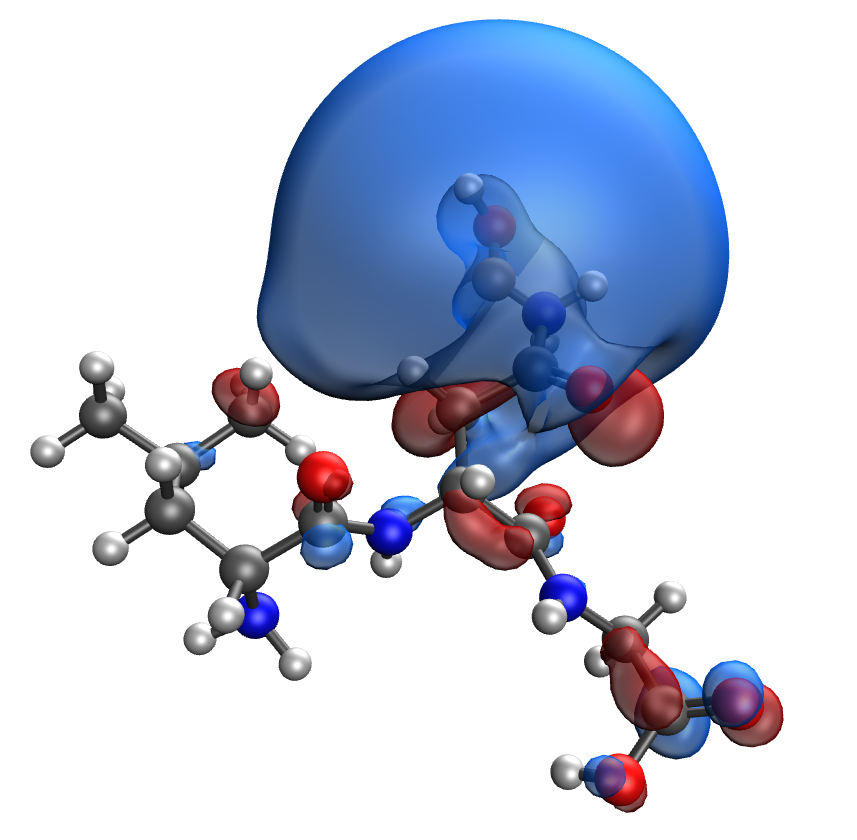 |
| d | e |

Fig. S12. Results of the quantum-chemical simulation of the *Leu-Asn-Gly* gelatin segment, bonded with CuO molecule: a model of the molecular complex (a), the distribution of electron density (b), electronic density distribution gradient (c), HOMO (d), LUMO (e)

| 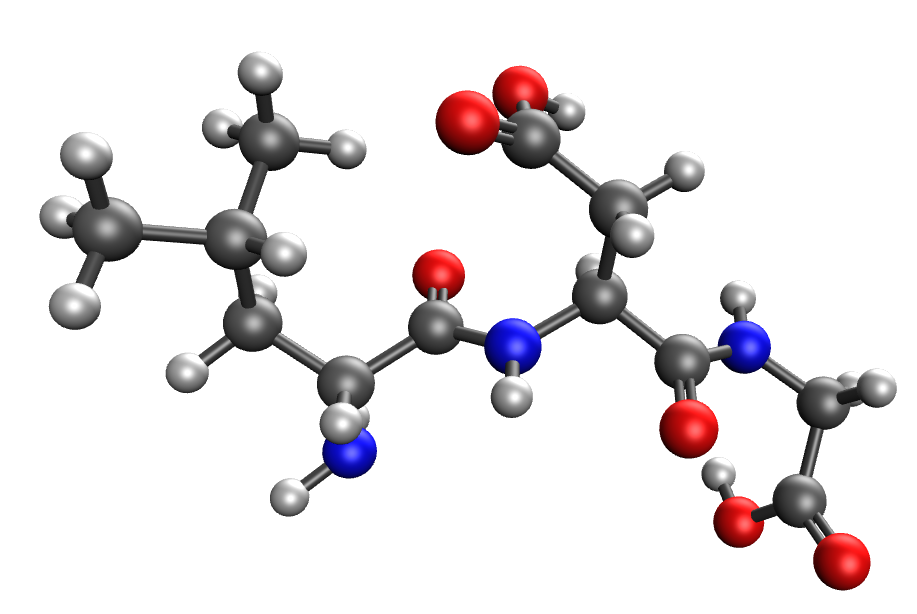 | 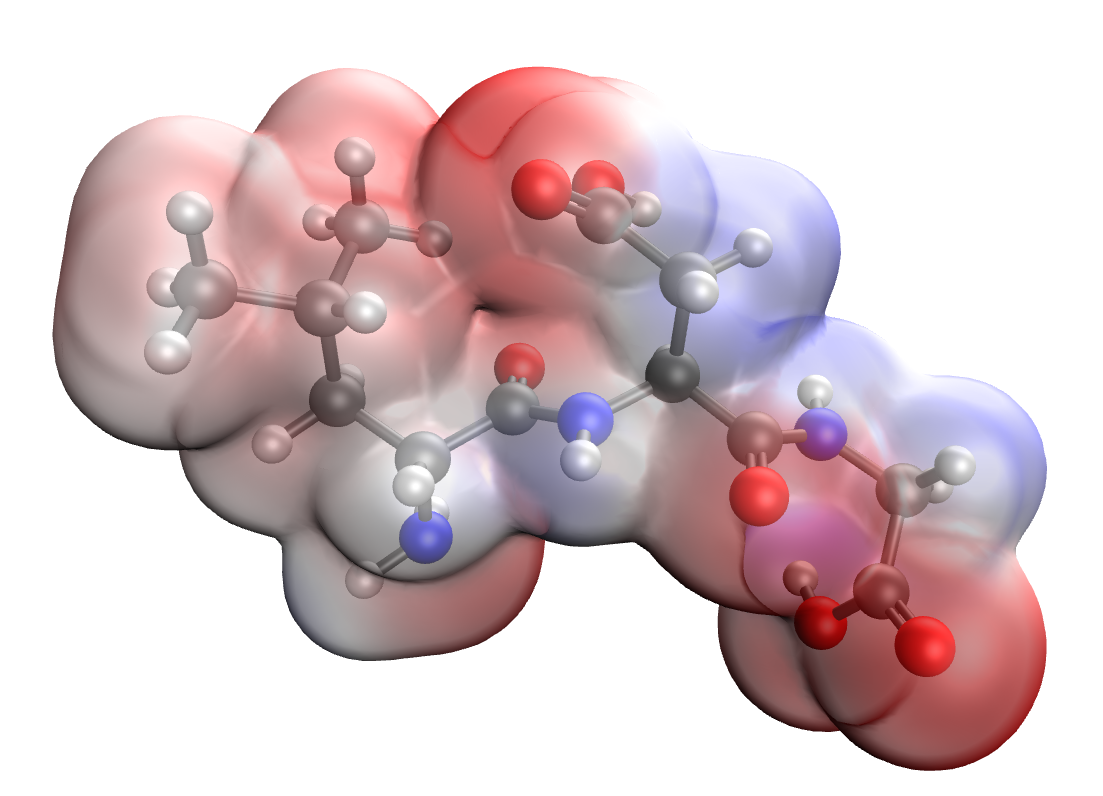 |
| --- | --- |
| a | b |
| 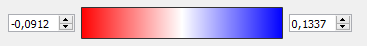 | |
| c | |
| 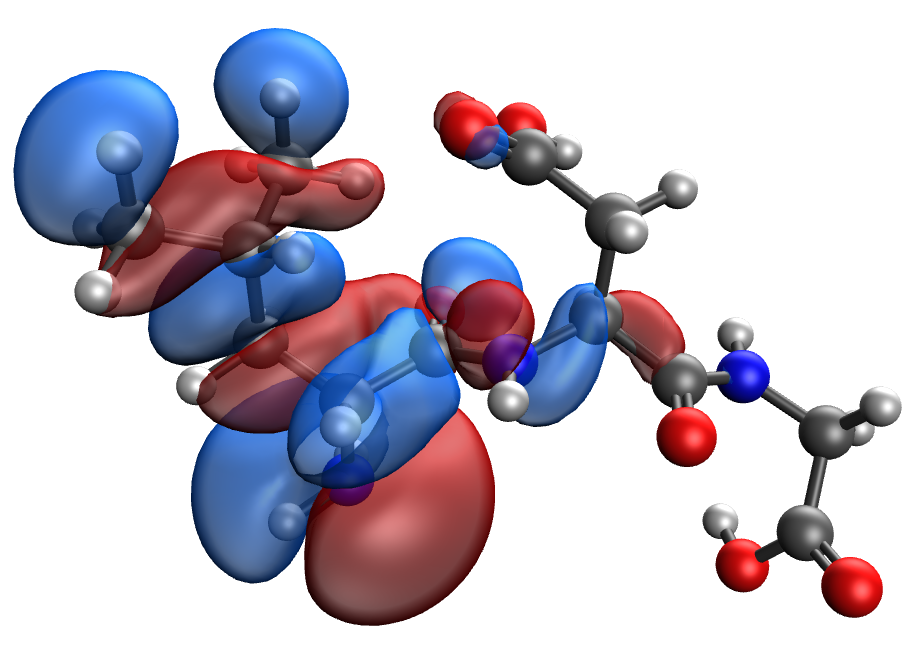 | 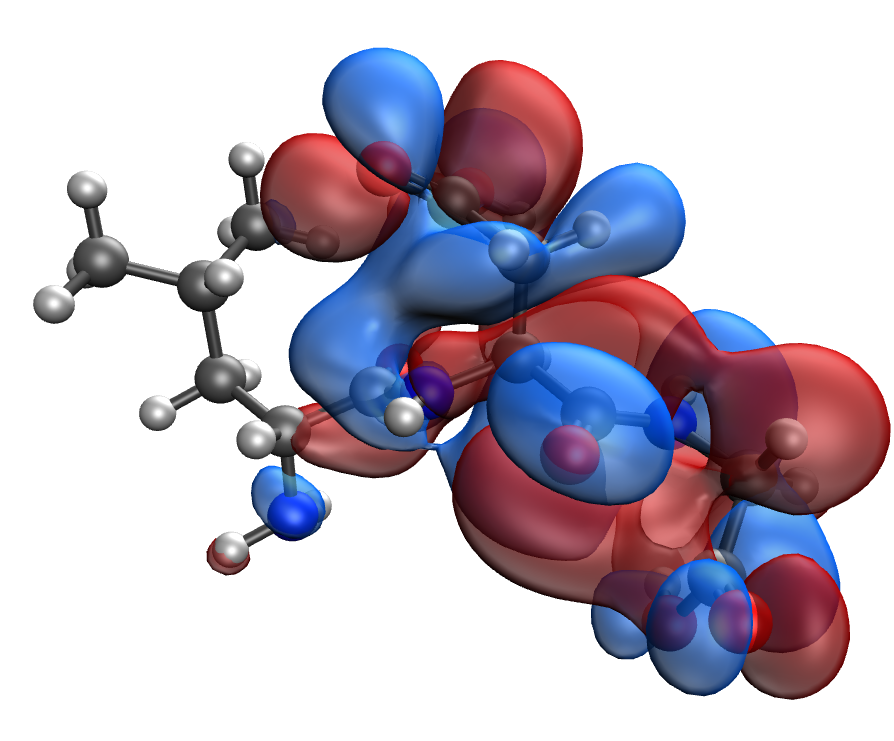 |
| d | e |

Fig. S13. Results of the quantum-chemical simulation of the Leu*-Asp-Gly* gelatin segment: a model of the molecular complex (a), the distribution of electron density (b), electronic density distribution gradient (c), HOMO (d), LUMO (e)

| 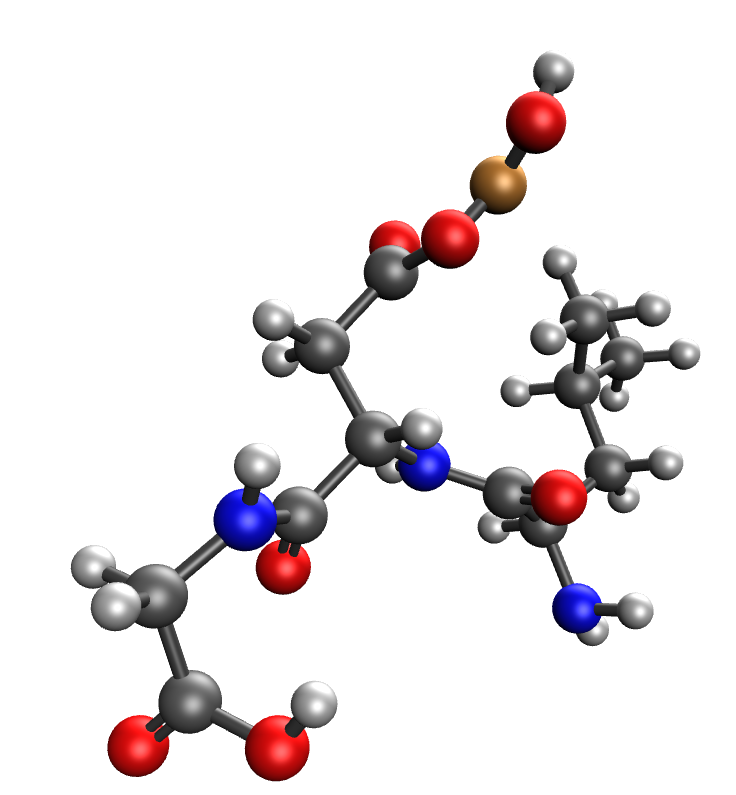 | 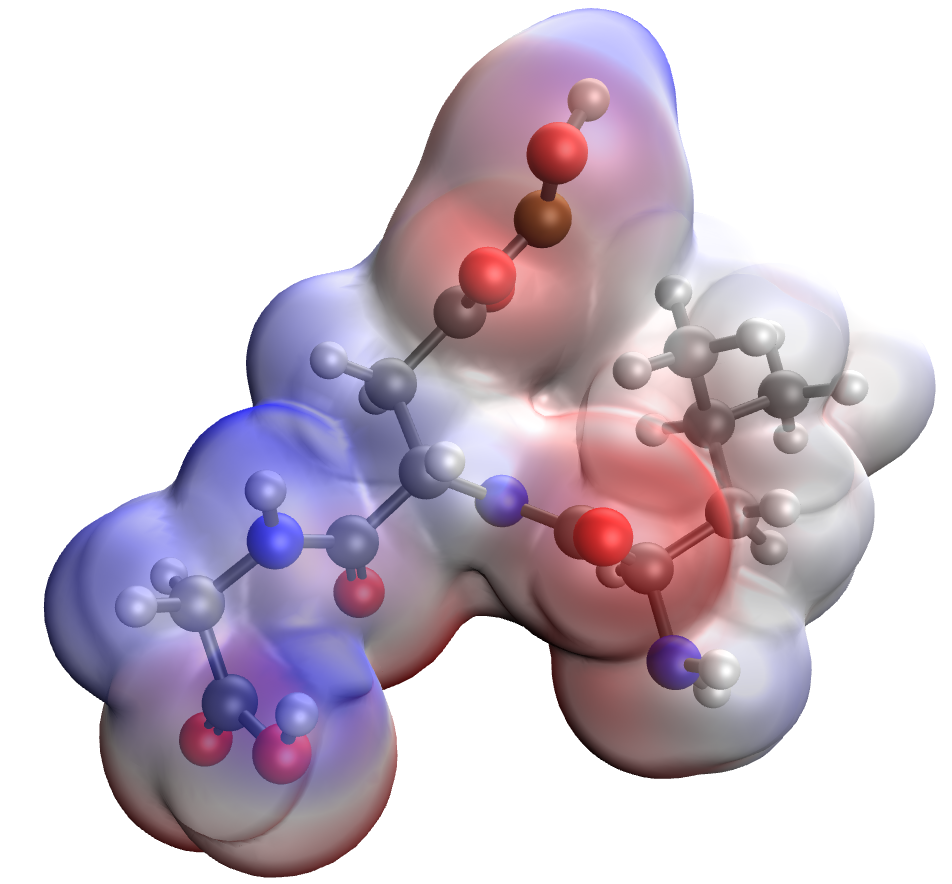 |
| --- | --- |
| a | b |
| 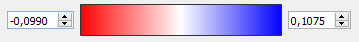 | |
| c | |
| 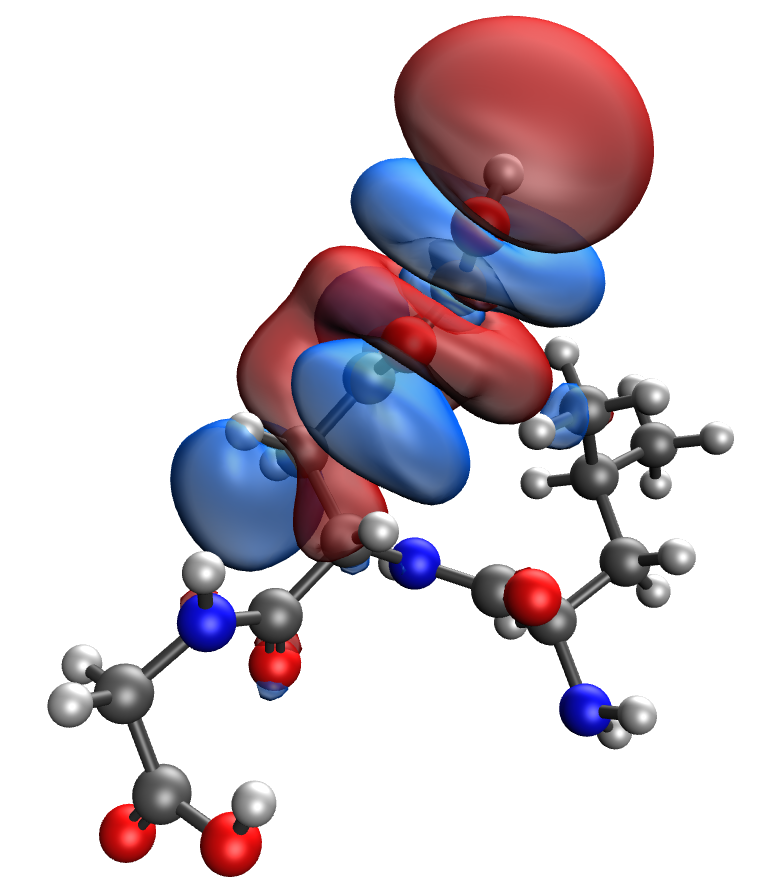 | 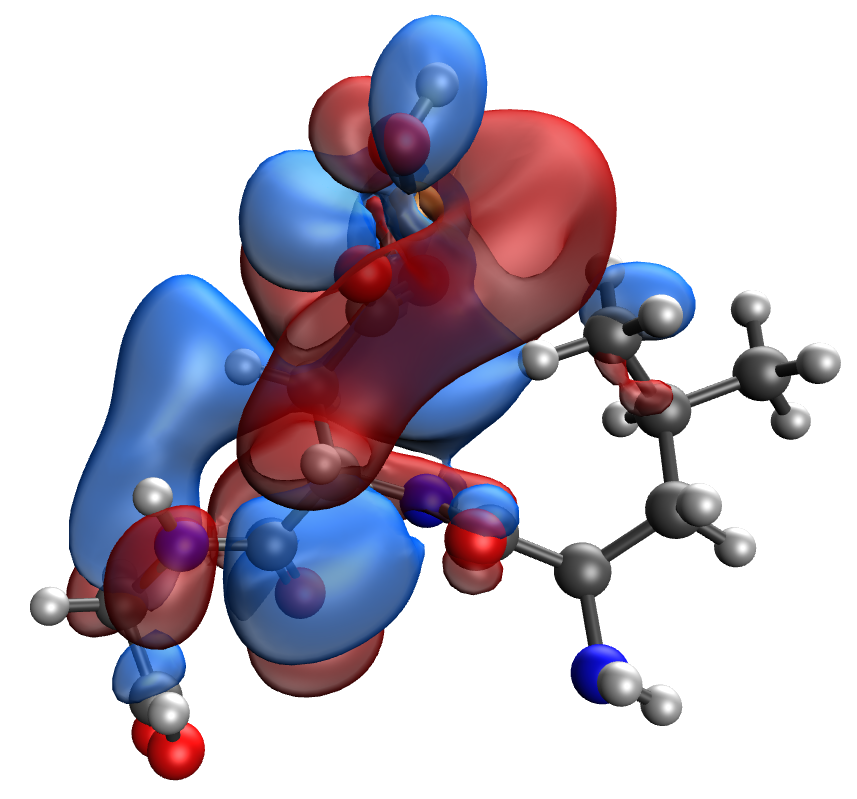 |
| d | e |

Fig. S14. Results of the quantum-chemical simulation of the *Leu-Asp-Gly* gelatin segment, bonded with CuO molecule: a model of the molecular complex (a), the distribution of electron density (b), electronic density distribution gradient (c), HOMO (d), LUMO (e)

| 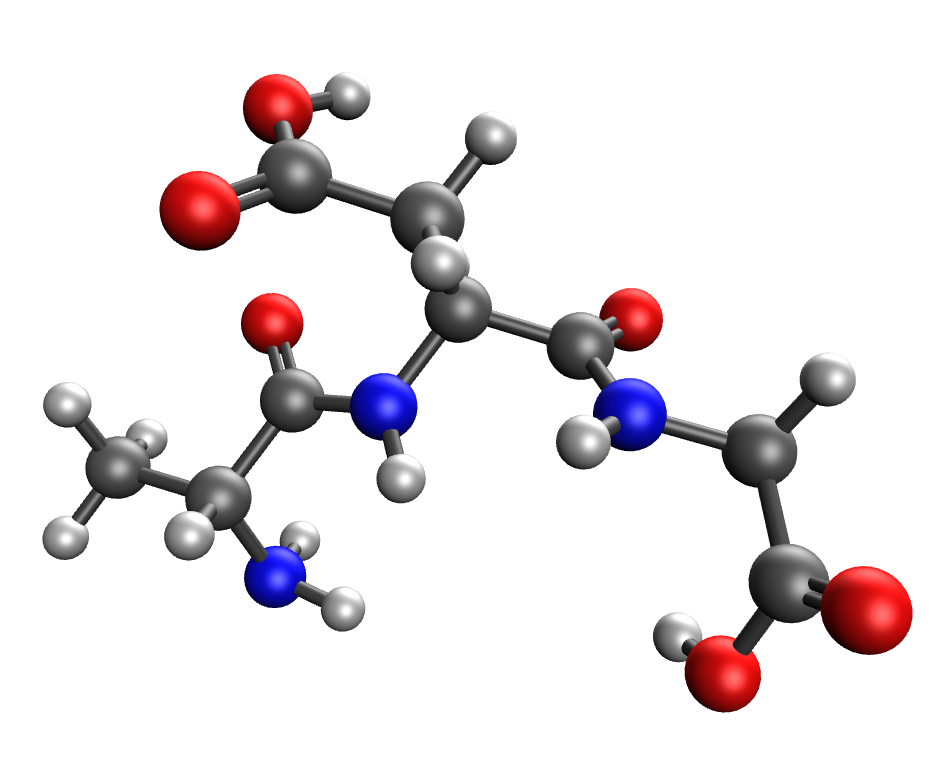 | 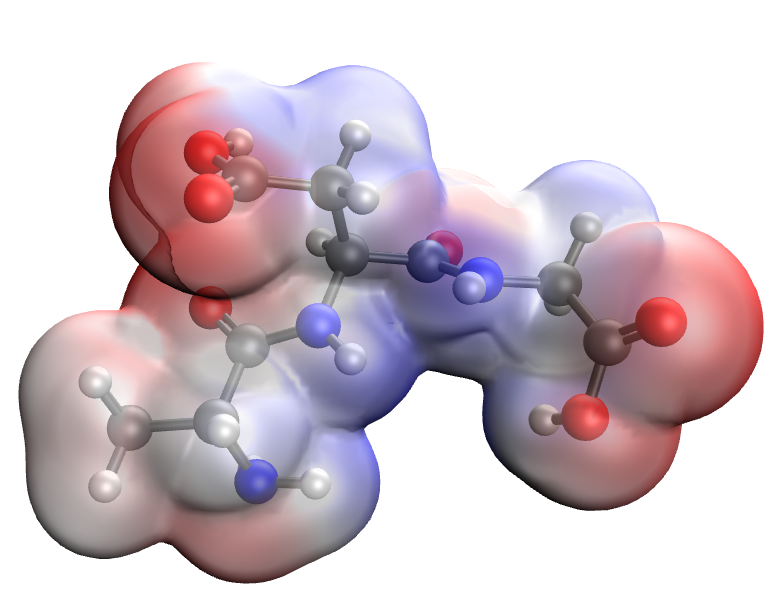 |
| --- | --- |
| a | b |
| 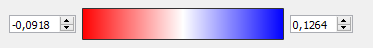 | |
| c | |
| 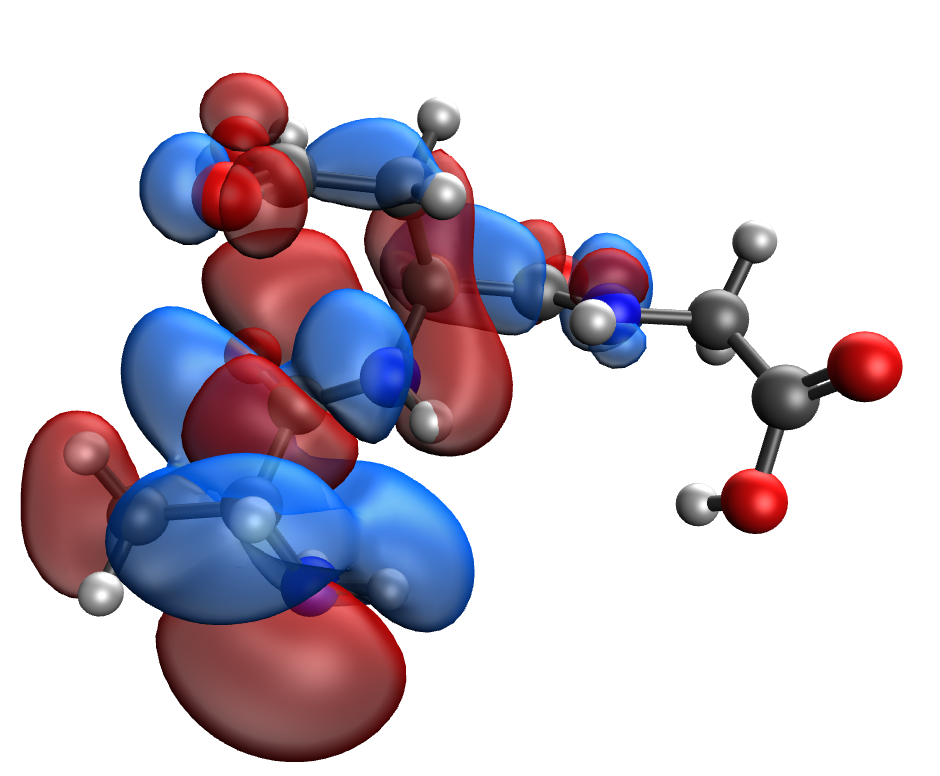 | 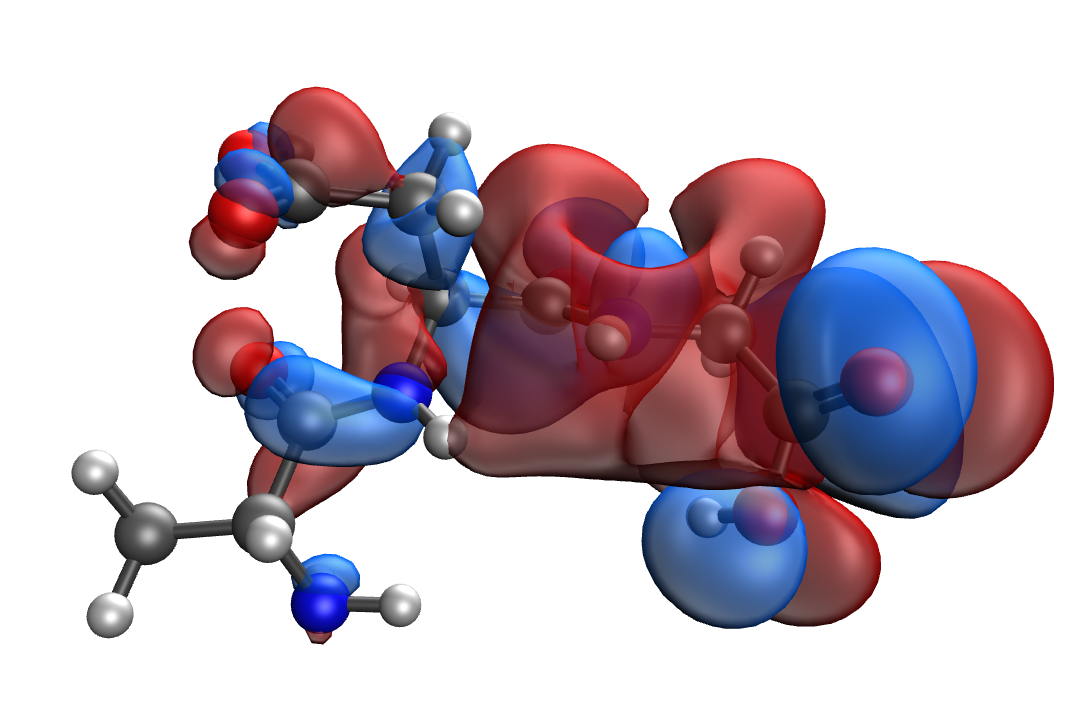 |
| d | e |

Fig. S15. Results of the quantum-chemical simulation of the Ala*-Asp-Gly* gelatin segment: a model of the molecular complex (a), the distribution of electron density (b), electronic density distribution gradient (c), HOMO (d), LUMO (e)

| 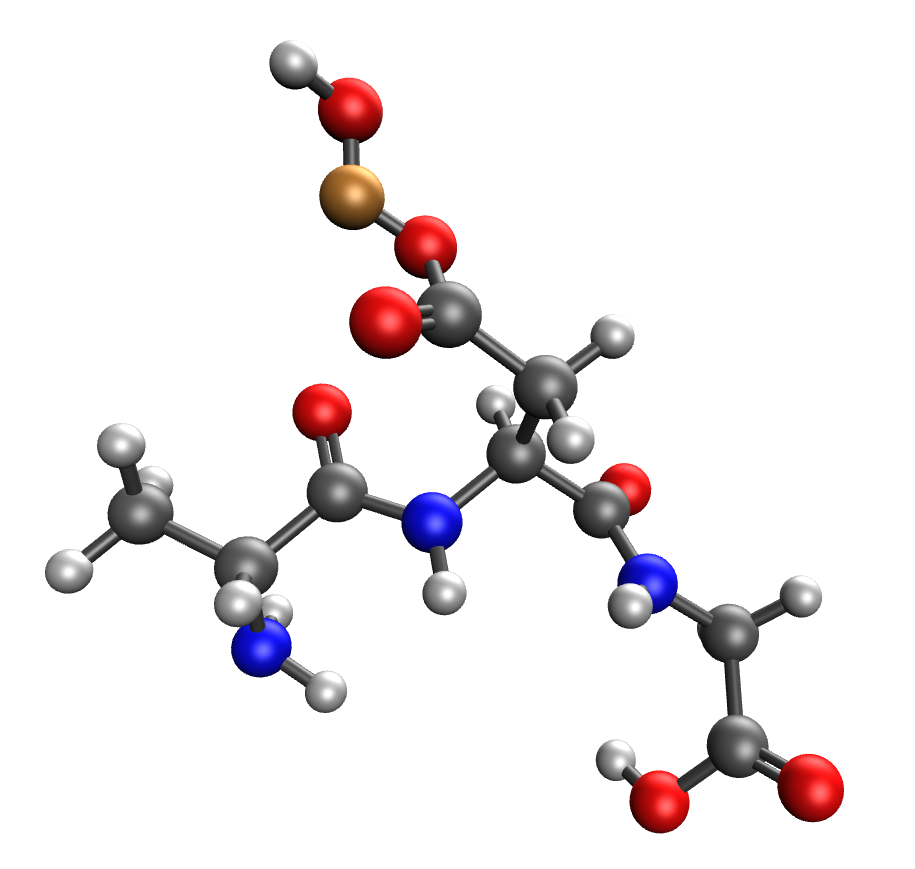 | 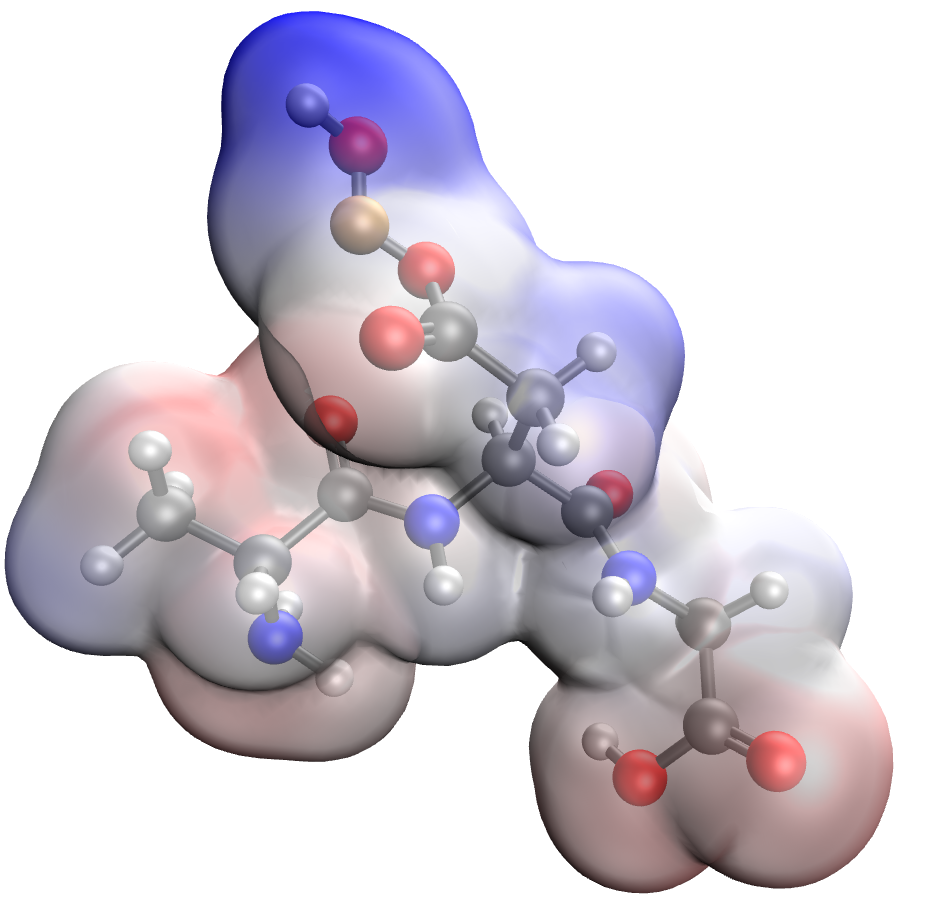 |
| --- | --- |
| a | b |
| 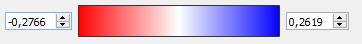 | |
| c | |
| 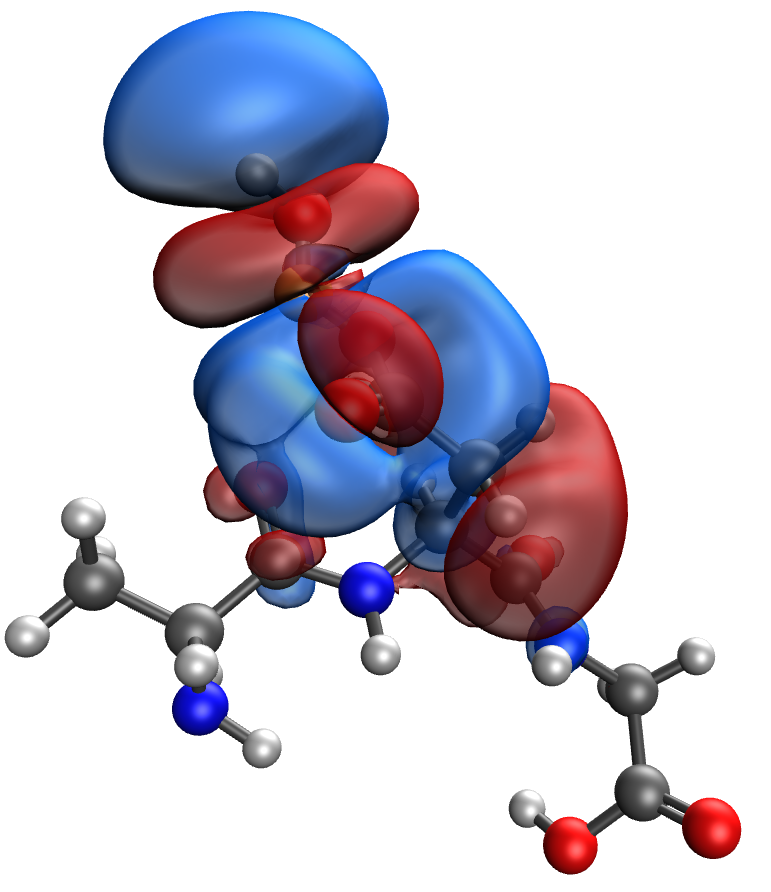 | 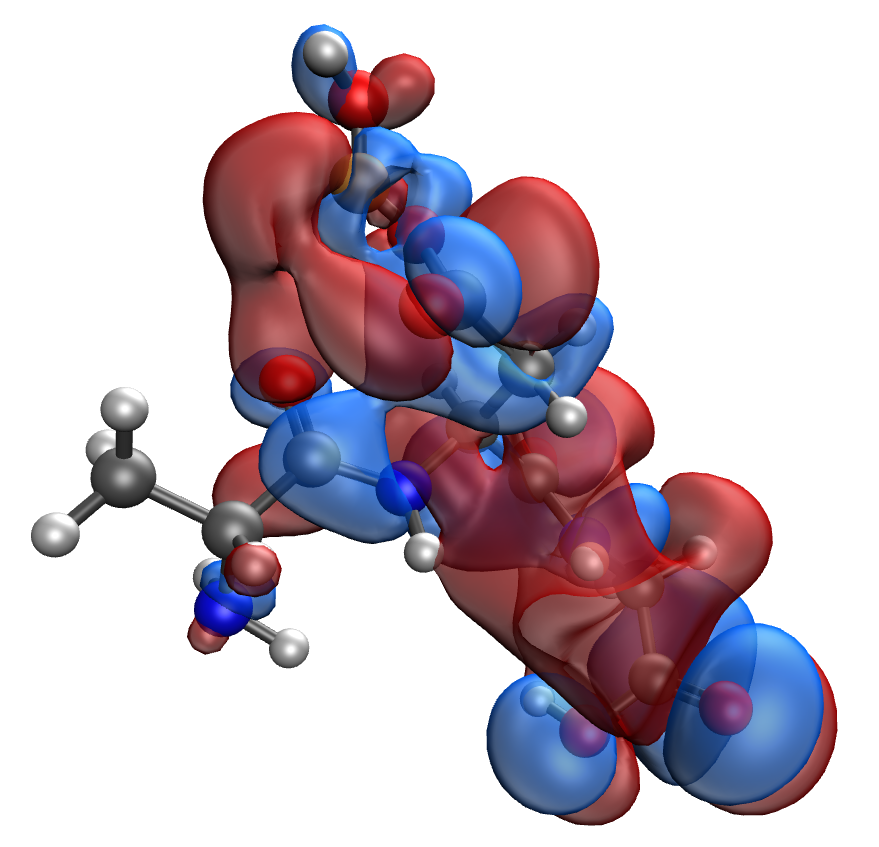 |
| d | e |

Fig. S16. Results of the quantum-chemical simulation of the *Ala-Asp-Gly* gelatin segment, bonded with CuO molecule: a model of the molecular complex (a), the distribution of electron density (b), electronic density distribution gradient (c), HOMO (d), LUMO (e)

| 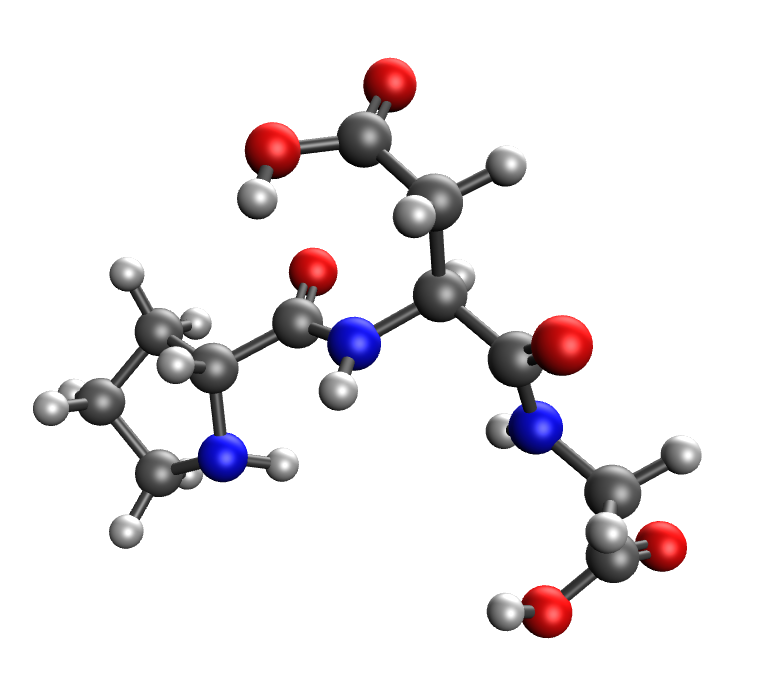 | 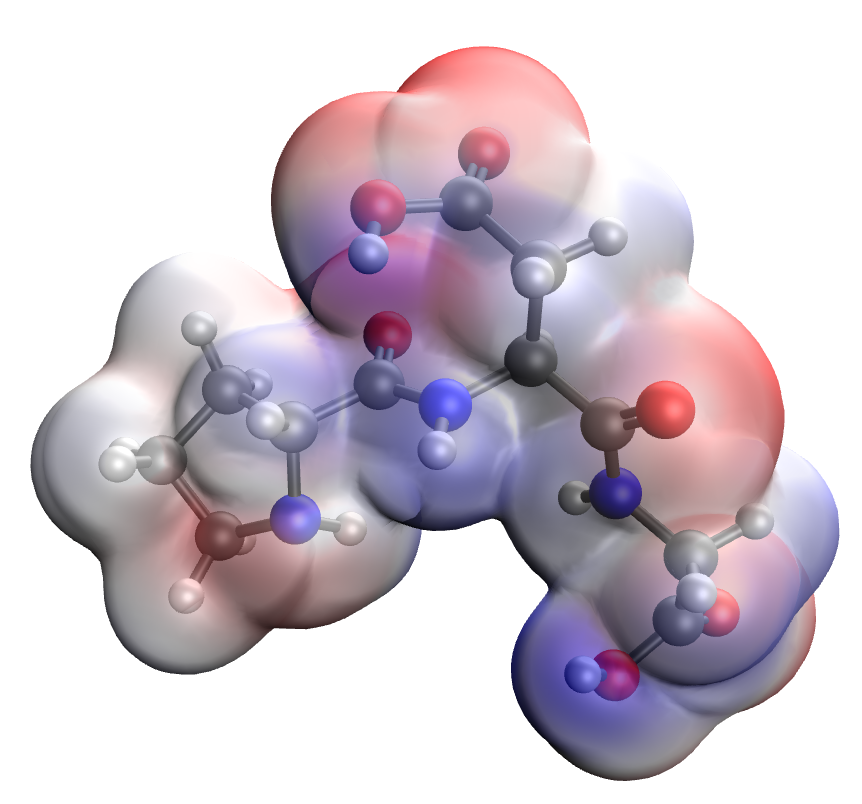 |
| --- | --- |
| a | b |
| 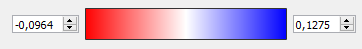 | |
| c | |
| 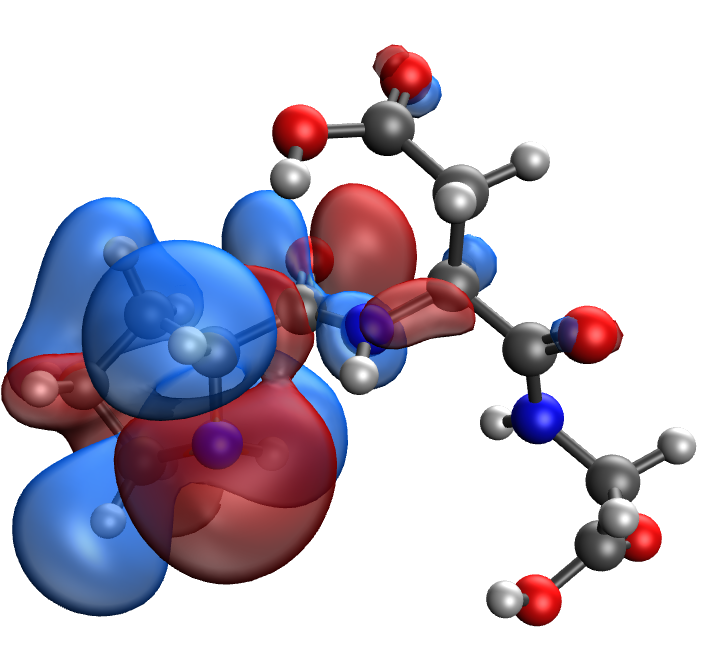 | 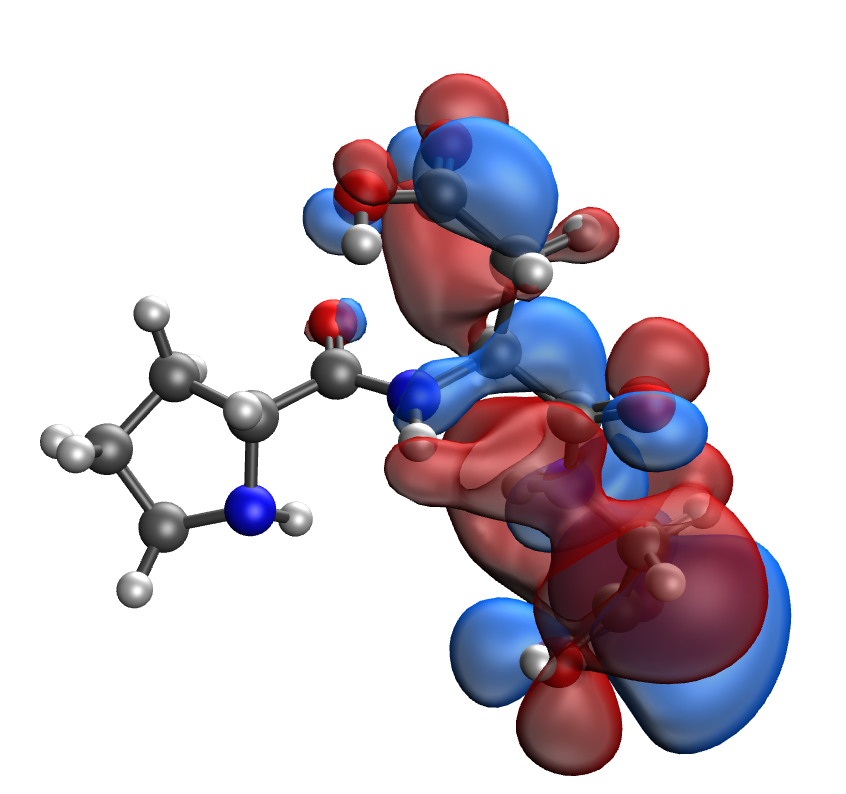 |
| d | e |

Fig. S17. Results of the quantum-chemical simulation of the Pro*-Asp-Gly* gelatin segment: a model of the molecular complex (a), the distribution of electron density (b), electronic density distribution gradient (c), HOMO (d), LUMO (e)

| 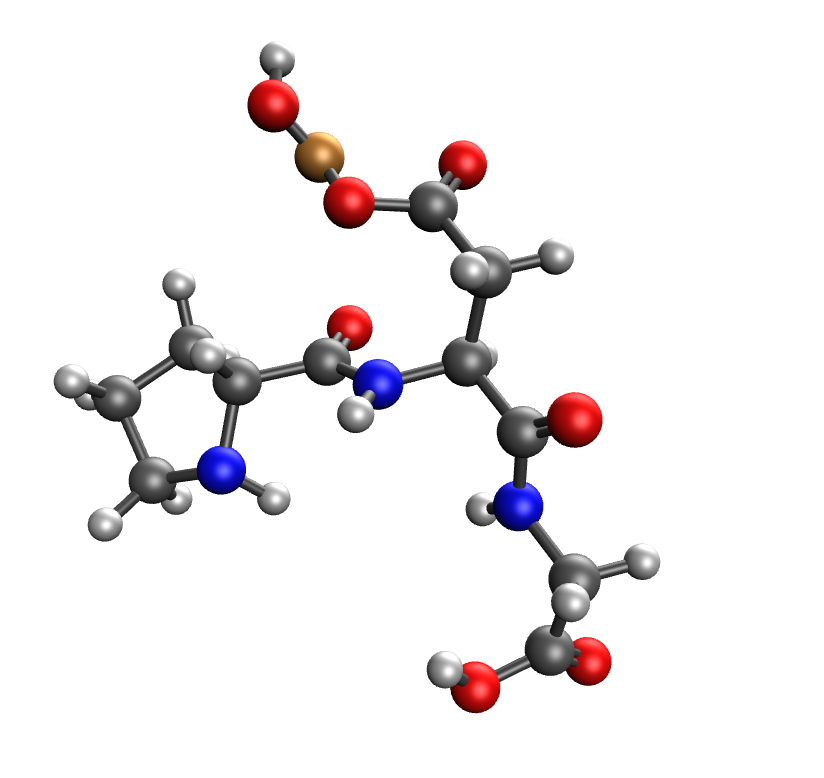 | 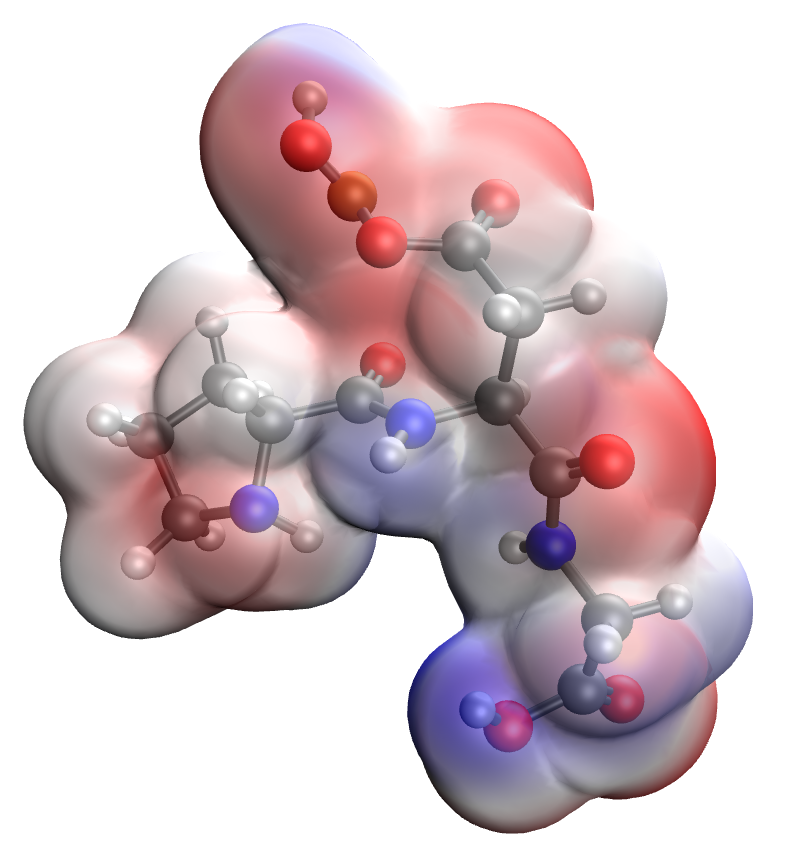 |
| --- | --- |
| a | b |
| 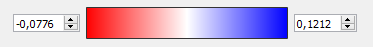 | |
| c | |
| 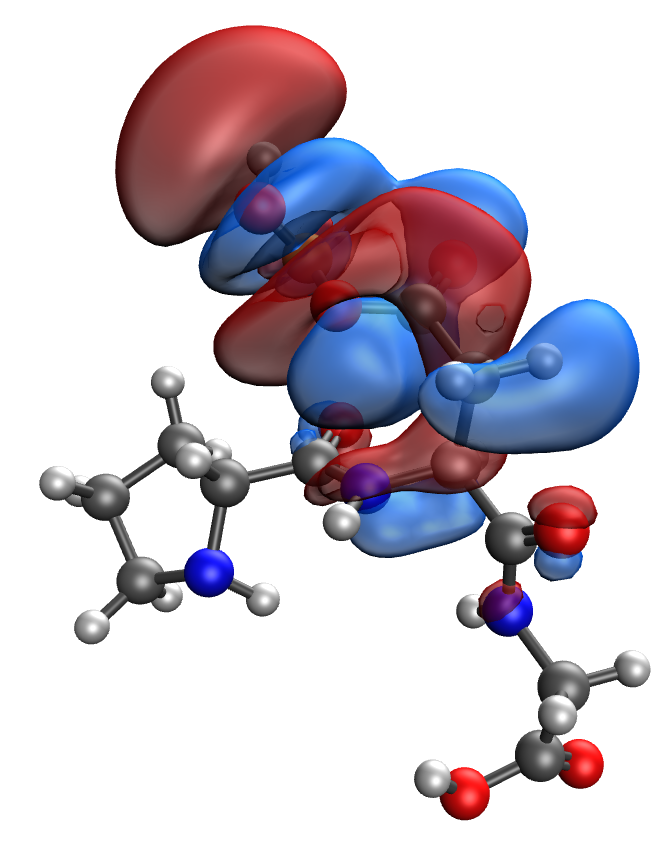 | 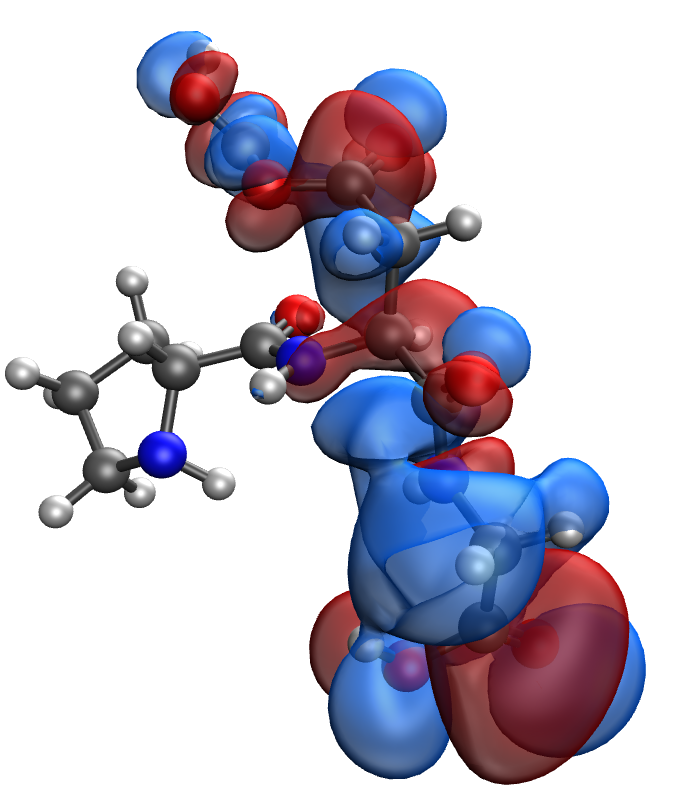 |
| d | e |

Fig. S18. Results of the quantum-chemical simulation of the *Pro-Asp-Gly* gelatin segment, bonded with CuO molecule: a model of the molecular complex (a), the distribution of electron density (b), electronic density distribution gradient (c), HOMO (d), LUMO (e)

| 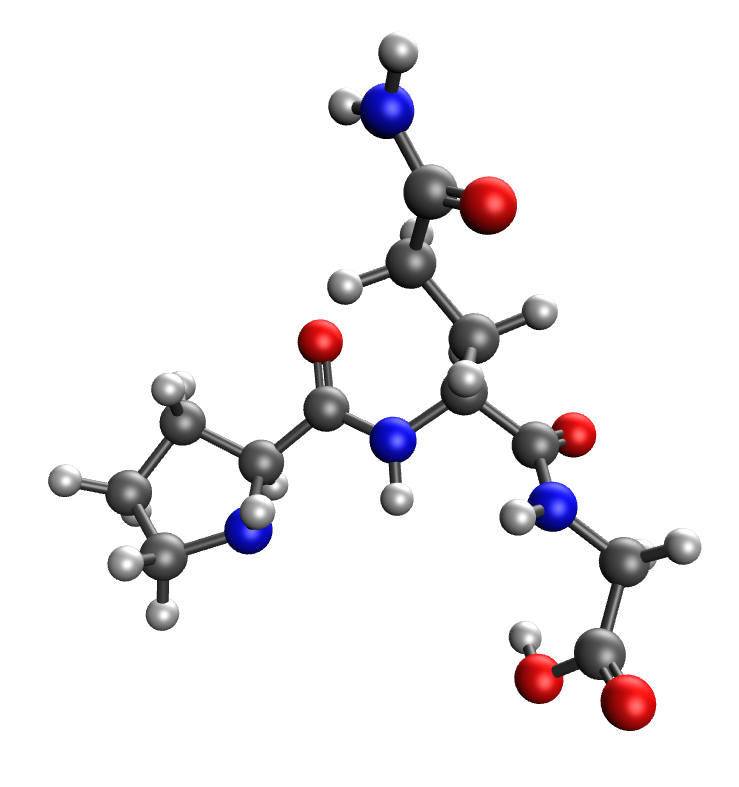 | 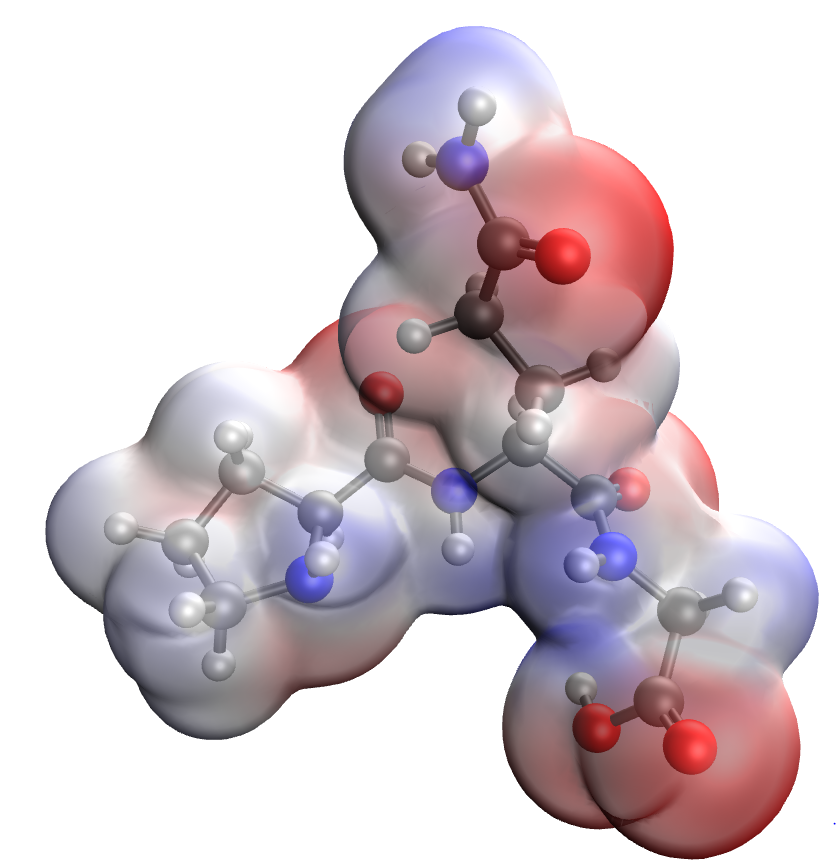 |
| --- | --- |
| a | b |
| 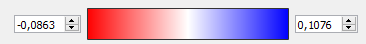 | |
| c | |
| 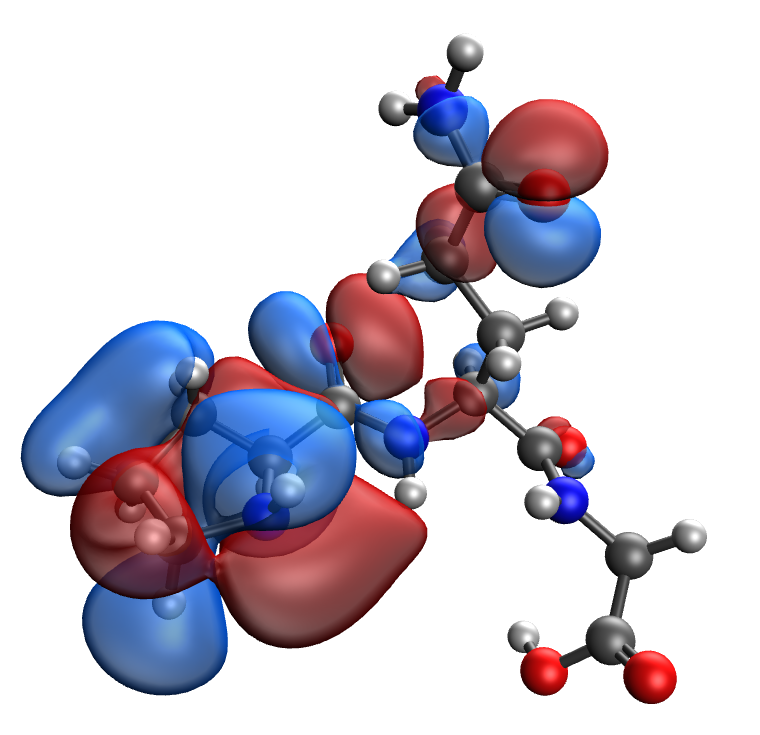 | 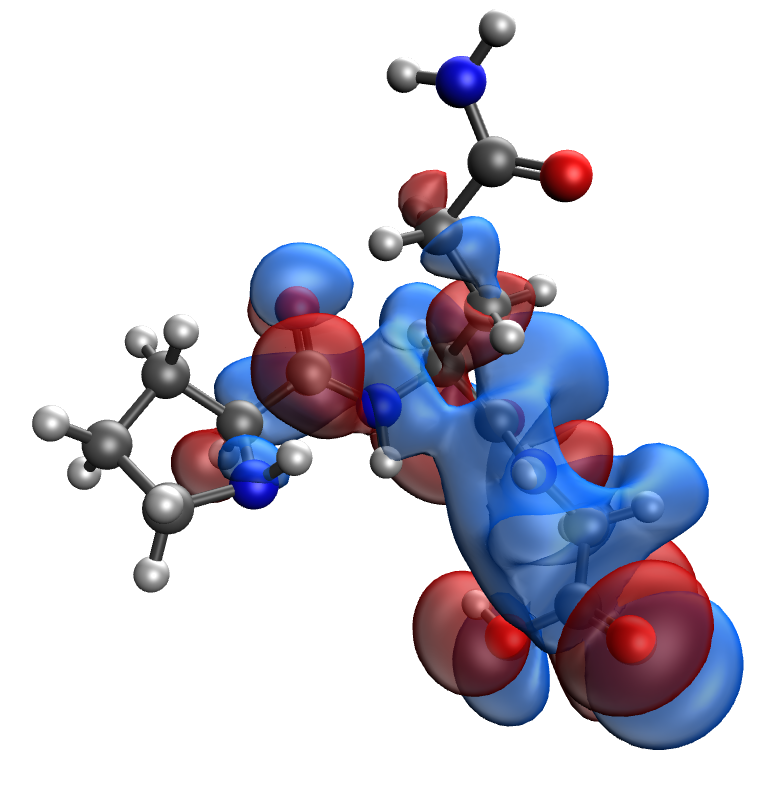 |
| d | e |

Fig. S19. Results of the quantum-chemical simulation of the Pro*-Gln-Gly* gelatin segment: a model of the molecular complex (a), the distribution of electron density (b), electronic density distribution gradient (c), HOMO (d), LUMO (e)

| 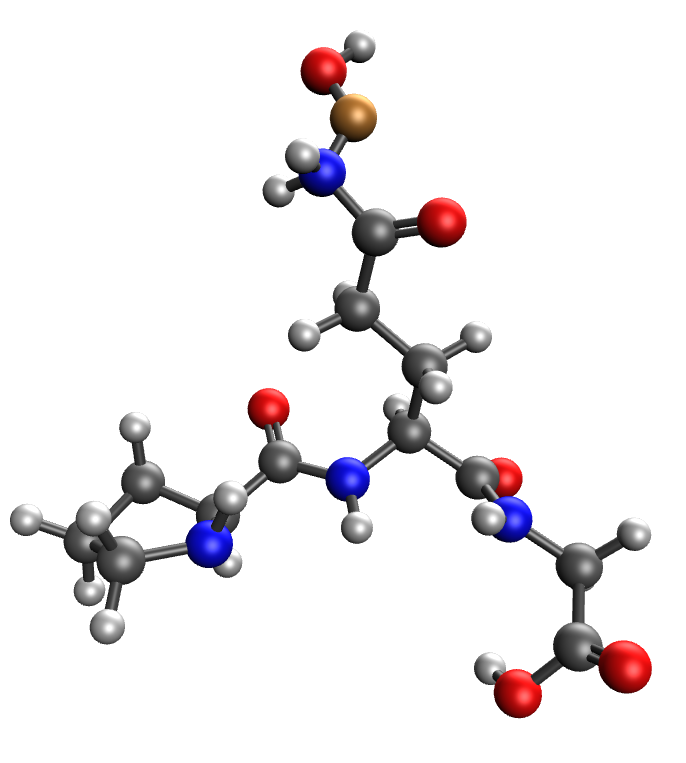 | 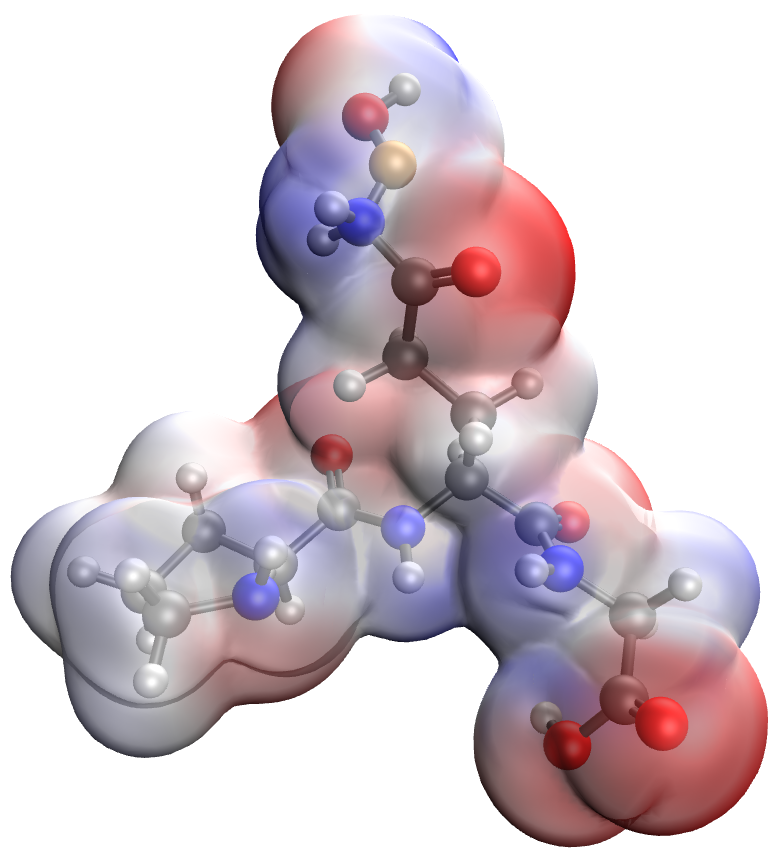 |
| --- | --- |
| a | b |
| 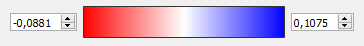 | |
| c | |
| 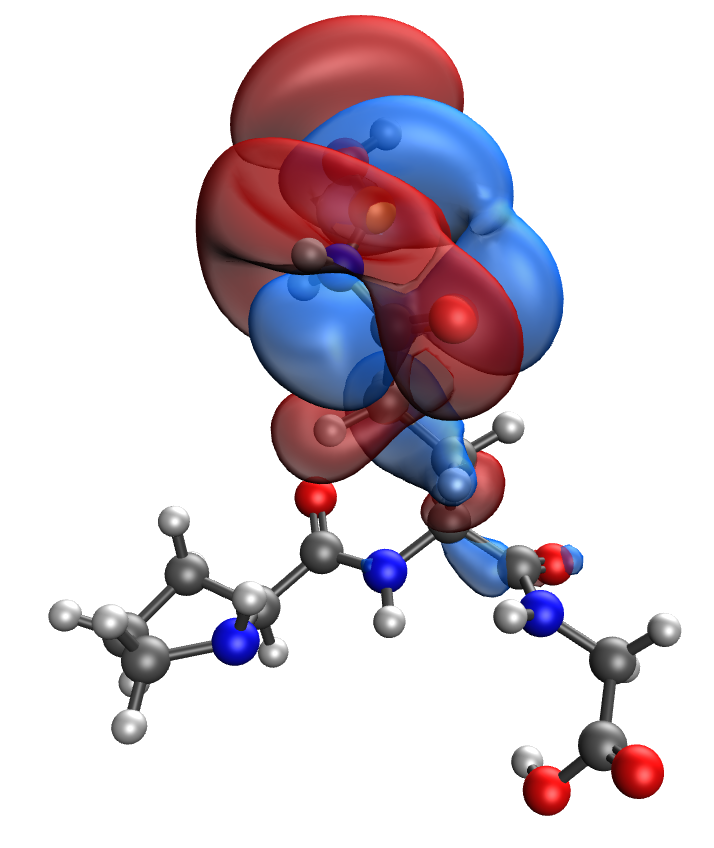 | 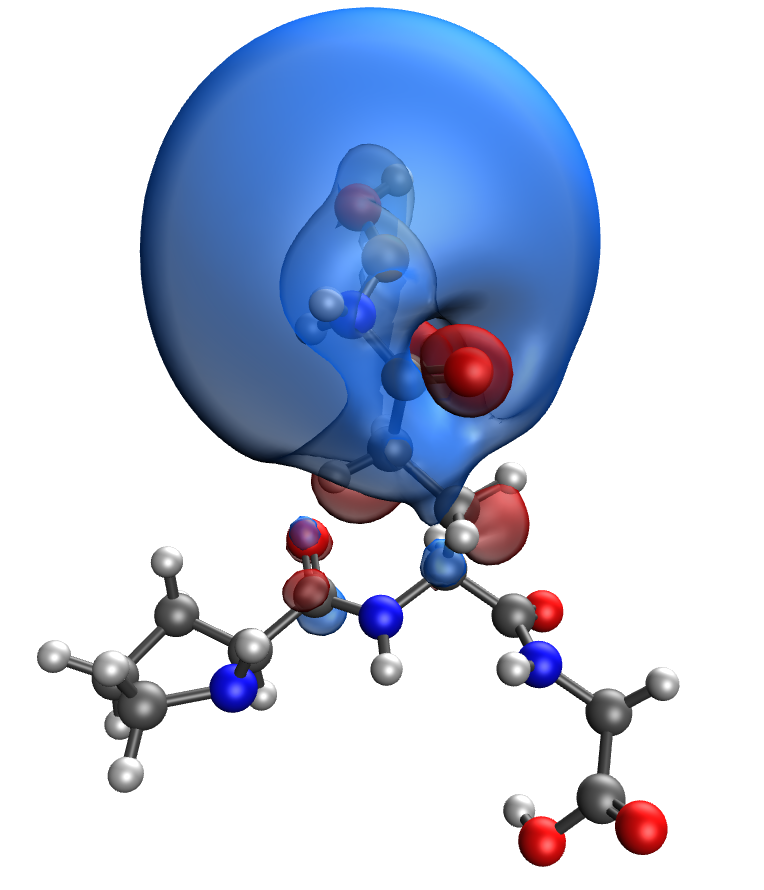 |
| d | e |

Fig. S20. Results of the quantum-chemical simulation of the *Pro-Gln-Gly* gelatin segment, bonded with CuO molecule: a model of the molecular complex (a), the distribution of electron density (b), electronic density distribution gradient (c), HOMO (d), LUMO (e)

|  |  |
| --- | --- |
| a | b |
|  | |
| c | |
|  |  |
| d | e |

Fig. S21. Results of the quantum-chemical simulation of the Phe*-Gln-Gly* gelatin segment: a model of the molecular complex (a), the distribution of electron density (b), electronic density distribution gradient (c), HOMO (d), LUMO (e)

|  |  |
| --- | --- |
| a | b |
|  | |
| c | |
|  |  |
| d | e |

Fig. S22. Results of the quantum-chemical simulation of the *Phe-Gln-Gly* gelatin segment, bonded with CuO molecule: a model of the molecular complex (a), the distribution of electron density (b), electronic density distribution gradient (c), HOMO (d), LUMO (e)

|  |  |
| --- | --- |
| a | b |
|  | |
| c | |
|  |  |
| d | e |

Fig. S23. Results of the quantum-chemical simulation of the Gly*-Gln-Met* gelatin segment: a model of the molecular complex (a), the distribution of electron density (b), electronic density distribution gradient (c), HOMO (d), LUMO (e)

|  |  |
| --- | --- |
| a | b |
|  | |
| c | |
|  |  |
| d | e |

Fig. S24. Results of the quantum-chemical simulation of the *Gly-Gln-Met* gelatin segment, bonded with CuO molecule: a model of the molecular complex (a), the distribution of electron density (b), electronic density distribution gradient (c), HOMO (d), LUMO (e)

|  |  |
| --- | --- |
| a | b |
|  | |
| c | |
|  |  |
| d | e |

Fig. S25. Results of the quantum-chemical simulation of the Val*-Gln-Gly* gelatin segment: a model of the molecular complex (a), the distribution of electron density (b), electronic density distribution gradient (c), HOMO (d), LUMO (e)

|  |  |
| --- | --- |
| a | b |
|  | |
| c | |
|  |  |
| d | e |

Fig. S26. Results of the quantum-chemical simulation of the *Val-Gln-Gly* gelatin segment, bonded with CuO molecule: a model of the molecular complex (a), the distribution of electron density (b), electronic density distribution gradient (c), HOMO (d), LUMO (e)

|  |  |
| --- | --- |
| a | b |
|  | |
| c | |
|  |  |
| d | e |

Fig. S27. Results of the quantum-chemical simulation of the Ala*-Gln-Gly* gelatin segment: a model of the molecular complex (a), the distribution of electron density (b), electronic density distribution gradient (c), HOMO (d), LUMO (e)

|  |  |
| --- | --- |
| a | b |
|  | |
| c | |
|  |  |
| d | e |

Fig. S28. Results of the quantum-chemical simulation of the *Ala-Gln-Gly* gelatin segment, bonded with CuO molecule: a model of the molecular complex (a), the distribution of electron density (b), electronic density distribution gradient (c), HOMO (d), LUMO (e)

|  |  |
| --- | --- |
| a | b |
|  | |
| c | |
|  |  |
| d | e |

Fig. S29. Results of the quantum-chemical simulation of the Leu*-Gln-Gly* gelatin segment: a model of the molecular complex (a), the distribution of electron density (b), electronic density distribution gradient (c), HOMO (d), LUMO (e)

|  |  |
| --- | --- |
| a | b |
|  | |
| c | |
|  |  |
| d | e |

Fig. S30. Results of the quantum-chemical simulation of the *Leu-Gln-Gly* gelatin segment, bonded with CuO molecule: a model of the molecular complex (a), the distribution of electron density (b), electronic density distribution gradient (c), HOMO (d), LUMO (e)

|  |  |
| --- | --- |
| a | b |
|  | |
| c | |
|  |  |
| d | e |

Fig. S31. Results of the quantum-chemical simulation of the Gly*-Glu-Ala* gelatin segment: a model of the molecular complex (a), the distribution of electron density (b), electronic density distribution gradient (c), HOMO (d), LUMO (e)

|  |  |
| --- | --- |
| a | b |
|  | |
| c | |
|  |  |
| d | e |

Fig. S32. Results of the quantum-chemical simulation of the *Gly-Glu-Ala* gelatin segment, bonded with CuO molecule: a model of the molecular complex (a), the distribution of electron density (b), electronic density distribution gradient (c), HOMO (d), LUMO (e)

|  |  |
| --- | --- |
| a | b |
|  | |
| c | |
|  |  |
| d | e |

Fig. S33. Results of the quantum-chemical simulation of the Val*-Glu-Gly* gelatin segment: a model of the molecular complex (a), the distribution of electron density (b), electronic density distribution gradient (c), HOMO (d), LUMO (e)

|  |  |
| --- | --- |
| a | b |
|  | |
| c | |
|  |  |
| d | e |

Fig. S34. Results of the quantum-chemical simulation of the *Val-Glu-Gly* gelatin segment, bonded with CuO molecule: a model of the molecular complex (a), the distribution of electron density (b), electronic density distribution gradient (c), HOMO (d), LUMO (e)

|  |  |
| --- | --- |
| a | b |
|  | |
| c | |
|  |  |
| d | e |

Fig. S35. Results of the quantum-chemical simulation of the Met*-Hyl-Gly* gelatin segment: a model of the molecular complex (a), the distribution of electron density (b), electronic density distribution gradient (c), HOMO (d), LUMO (e)

|  |  |
| --- | --- |
| a | b |
|  | |
| c | |
|  |  |
| d | e |

Fig. S36. Results of the quantum-chemical simulation of the *Met-Hyl-Gly* gelatin segment, bonded with CuO molecule: a model of the molecular complex (a), the distribution of electron density (b), electronic density distribution gradient (c), HOMO (d), LUMO (e)

|  |  |
| --- | --- |
| a | b |
|  | |
| c | |
|  |  |
| d | e |

Fig. S37. Results of the quantum-chemical simulation of the *Met-Hyl-Gly* gelatin segment: a model of the molecular complex (a), the distribution of electron density (b), electronic density distribution gradient (c), HOMO (d), LUMO (e)

|  |  |
| --- | --- |
| a | b |
|  | |
| c | |
|  |  |
| d | e |

Fig. S38. Results of the quantum-chemical simulation of the Ile*-Hyl-Gly* gelatin segment, bonded with CuO molecule: a model of the molecular complex (a), the distribution of electron density (b), electronic density distribution gradient (c), HOMO (d), LUMO (e)

|  |  |
| --- | --- |
| a | b |
|  | |
| c | |
|  |  |
| d | e |

Fig. S39. Results of the quantum-chemical simulation of the *Ile-Hyl-Gly* gelatin segment: a model of the molecular complex (a), the distribution of electron density (b), electronic density distribution gradient (c), HOMO (d), LUMO (e)

|  |  |
| --- | --- |
| a | b |
|  | |
| c | |
|  |  |
| d | e |

Fig. S40. Results of the quantum-chemical simulation of the *Met-Hyl-Gly* gelatin segment, bonded with CuO molecule: a model of the molecular complex (a), the distribution of electron density (b), electronic density distribution gradient (c), HOMO (d), LUMO (e)

|  |  |
| --- | --- |
| a | b |
|  | |
| c | |
|  |  |
| d | e |

Fig. S41. Results of the quantum-chemical simulation of the Leu*-Hyp-Gly* gelatin segment: a model of the molecular complex (a), the distribution of electron density (b), electronic density distribution gradient (c), HOMO (d), LUMO (e)

|  |  |
| --- | --- |
| a | b |
|  | |
| c | |
|  |  |
| d | e |

Fig. S42. Results of the quantum-chemical simulation of the *Leu-Hyp-Gly* gelatin segment, bonded with CuO molecule: a model of the molecular complex (a), the distribution of electron density (b), electronic density distribution gradient (c), HOMO (d), LUMO (e)

|  |  |
| --- | --- |
| a | b |
|  | |
| c | |
|  |  |
| d | e |

Fig. S43. Results of the quantum-chemical simulation of the Pro*-Hyp-Gly* gelatin segment: a model of the molecular complex (a), the distribution of electron density (b), electronic density distribution gradient (c), HOMO (d), LUMO (e)

|  |  |
| --- | --- |
| a | b |
|  | |
| c | |
|  |  |
| d | e |

Fig. S44. Results of the quantum-chemical simulation of the *Pro-Hyp-Gly* gelatin segment, bonded with CuO molecule: a model of the molecular complex (a), the distribution of electron density (b), electronic density distribution gradient (c), HOMO (d), LUMO (e)

|  |  |
| --- | --- |
| a | b |
|  | |
| c | |
|  |  |
| d | e |

Fig. S45. Results of the quantum-chemical simulation of the Ala*-Hyp-Gly* gelatin segment: a model of the molecular complex (a), the distribution of electron density (b), electronic density distribution gradient (c), HOMO (d), LUMO (e)

|  |  |
| --- | --- |
| a | b |
|  | |
| c | |
|  |  |
| d | e |

Fig. S46. Results of the quantum-chemical simulation of the *Ala-Hyp-Gly* gelatin segment, bonded with CuO molecule: a model of the molecular complex (a), the distribution of electron density (b), electronic density distribution gradient (c), HOMO (d), LUMO (e)

|  |  |
| --- | --- |
| a | b |
|  | |
| c | |
|  |  |
| d | e |

Fig. S47. Results of the quantum-chemical simulation of the Phe*-Hyp-Gly* gelatin segment: a model of the molecular complex (a), the distribution of electron density (b), electronic density distribution gradient (c), HOMO (d), LUMO (e)

|  |  |
| --- | --- |
| a | b |
|  | |
| c | |
|  |  |
| d | e |

Fig. S48. Results of the quantum-chemical simulation of the *Phe-Hyp-Gly* gelatin segment, bonded with CuO molecule: a model of the molecular complex (a), the distribution of electron density (b), electronic density distribution gradient (c), HOMO (d), LUMO (e)

|  |  |
| --- | --- |
| a | b |
|  | |
| c | |
|  |  |
| d | e |

Fig. S49. Results of the quantum-chemical simulation of the Val*-Hyp-Gly* gelatin segment: a model of the molecular complex (a), the distribution of electron density (b), electronic density distribution gradient (c), HOMO (d), LUMO (e)

|  |  |
| --- | --- |
| a | b |
|  | |
| c | |
|  |  |
| d | e |

Fig. S50. Results of the quantum-chemical simulation of the *Val-Hyp-Gly* gelatin segment, bonded with CuO molecule: a model of the molecular complex (a), the distribution of electron density (b), electronic density distribution gradient (c), HOMO (d), LUMO (e)

|  |  |
| --- | --- |
| a | b |
|  | |
| c | |
|  |  |
| d | e |

Fig. S51. Results of the quantum-chemical simulation of the Met*-Hyp-Gly* gelatin segment: a model of the molecular complex (a), the distribution of electron density (b), electronic density distribution gradient (c), HOMO (d), LUMO (e)

|  |  |
| --- | --- |
| a | b |
|  | |
| c | |
|  |  |
| d | e |

Fig. S52. Results of the quantum-chemical simulation of the *Met-Hyp-Gly* gelatin segment, bonded with CuO molecule: a model of the molecular complex (a), the distribution of electron density (b), electronic density distribution gradient (c), HOMO (d), LUMO (e)

|  |  |
| --- | --- |
| a | b |
|  | |
| c | |
|  |  |
| d | e |

Fig. S53. Results of the quantum-chemical simulation of the Ala*-Lys-Gly* gelatin segment: a model of the molecular complex (a), the distribution of electron density (b), electronic density distribution gradient (c), HOMO (d), LUMO (e)

|  |  |
| --- | --- |
| a | b |
|  | |
| c | |
|  |  |
| d | e |

Fig. S54. Results of the quantum-chemical simulation of the *Ala-Lys-Gly* gelatin segment, bonded with CuO molecule: a model of the molecular complex (a), the distribution of electron density (b), electronic density distribution gradient (c), HOMO (d), LUMO (e)

|  |  |
| --- | --- |
| a | b |
|  | |
| c | |
|  |  |
| d | e |

Fig. S55. Results of the quantum-chemical simulation of the Pro*-Lys-Gly* gelatin segment: a model of the molecular complex (a), the distribution of electron density (b), electronic density distribution gradient (c), HOMO (d), LUMO (e)

|  |  |
| --- | --- |
| a | b |
|  | |
| c | |
|  |  |
| d | e |

Fig. S56. Results of the quantum-chemical simulation of the *Pro-Lys-Gly* gelatin segment, bonded with CuO molecule: a model of the molecular complex (a), the distribution of electron density (b), electronic density distribution gradient (c), HOMO (d), LUMO (e)

|  |  |
| --- | --- |
| a | b |
|  | |
| c | |
|  |  |
| d | e |

Fig. S57. Results of the quantum-chemical simulation of the Pro*-Ser-Gly* gelatin segment: a model of the molecular complex (a), the distribution of electron density (b), electronic density distribution gradient (c), HOMO (d), LUMO (e)

|  |  |
| --- | --- |
| a | b |
|  | |
| c | |
|  |  |
| d | e |

Fig. S58. Results of the quantum-chemical simulation of the *Pro-Ser-Gly* gelatin segment, bonded with CuO molecule: a model of the molecular complex (a), the distribution of electron density (b), electronic density distribution gradient (c), HOMO (d), LUMO (e)

|  |  |
| --- | --- |
| a | b |
|  | |
| c | |
|  |  |
| d | e |

Fig. S59. Results of the quantum-chemical simulation of the Ala*-Ser-Gly* gelatin segment: a model of the molecular complex (a), the distribution of electron density (b), electronic density distribution gradient (c), HOMO (d), LUMO (e)

|  |  |
| --- | --- |
| a | b |
|  | |
| c | |
|  |  |
| d | e |

Fig. S60. Results of the quantum-chemical simulation of the *Ala-Ser-Gly* gelatin segment, bonded with CuO molecule: a model of the molecular complex (a), the distribution of electron density (b), electronic density distribution gradient (c), HOMO (d), LUMO (e)

|  |  |
| --- | --- |
| a | b |
|  | |
| c | |
|  |  |
| d | e |

Fig. S61. Results of the quantum-chemical simulation of the Phe*-Ser-Gly* gelatin segment: a model of the molecular complex (a), the distribution of electron density (b), electronic density distribution gradient (c), HOMO (d), LUMO (e)

|  |  |
| --- | --- |
| a | b |
|  | |
| c | |
|  |  |
| d | e |

Fig. S62. Results of the quantum-chemical simulation of the *Phe-Ser-Gly* gelatin segment, bonded with CuO molecule: a model of the molecular complex (a), the distribution of electron density (b), electronic density distribution gradient (c), HOMO (d), LUMO (e)

|  |  |
| --- | --- |
| a | b |
|  | |
| c | |
|  |  |
| d | e |

Fig. S63. Results of the quantum-chemical simulation of the Pro*-Thr-Gly* gelatin segment: a model of the molecular complex (a), the distribution of electron density (b), electronic density distribution gradient (c), HOMO (d), LUMO (e)

|  |  |
| --- | --- |
| a | b |
|  | |
| c | |
|  |  |
| d | e |

Fig. S64. Results of the quantum-chemical simulation of the *Pro-Thr-Gly* gelatin segment, bonded with CuO molecule: a model of the molecular complex (a), the distribution of electron density (b), electronic density distribution gradient (c), HOMO (d), LUMO (e)

|  |  |
| --- | --- |
| a | b |
|  | |
| c | |
|  |  |
| d | e |

Fig. S65. Results of the quantum-chemical simulation of the Leu*-Thr-Gly* gelatin segment: a model of the molecular complex (a), the distribution of electron density (b), electronic density distribution gradient (c), HOMO (d), LUMO (e)

|  |  |
| --- | --- |
| a | b |
|  | |
| c | |
|  |  |
| d | e |

Fig. S66. Results of the quantum-chemical simulation of the *Leu-Thr-Gly* gelatin segment, bonded with CuO molecule: a model of the molecular complex (a), the distribution of electron density (b), electronic density distribution gradient (c), HOMO (d), LUMO (e)

|  |  |
| --- | --- |
| a | b |
|  | |
| c | |
|  |  |
| d | e |

Fig. S67. Results of the quantum-chemical simulation of the Ala*-Thr-Gly* gelatin segment: a model of the molecular complex (a), the distribution of electron density (b), electronic density distribution gradient (c), HOMO (d), LUMO (e)

|  |  |
| --- | --- |
| a | b |
|  | |
| c | |
|  |  |
| d | e |

Fig. S68. Results of the quantum-chemical simulation of the *Ala-Thr-Gly* gelatin segment, bonded with CuO molecule: a model of the molecular complex (a), the distribution of electron density (b), electronic density distribution gradient (c), HOMO (d), LUMO (e)

Fig. S69. Energy-dispersive spectrum of methylcellulose film (control)

Fig. S70. Energy-dispersive spectrum of methylcellulose film modified with 0.2% CuO nanoparticles

Fig. S71. Energy-dispersive spectrum of methylcellulose film modified with 0.4% CuO nanoparticles
